# Effective and Reversible Carbon Dioxide Insertion into Cerium Pyrazolates

**DOI:** 10.1002/anie.201916483

**Published:** 2020-01-30

**Authors:** Uwe Bayer, Daniel Werner, Cäcilia Maichle‐Mössmer, Reiner Anwander

**Affiliations:** ^1^ Institut für Anorganische Chemie Eberhard Karls Universität Tübingen Auf der Morgenstelle 18 72076 Tübingen Germany

**Keywords:** carbon dioxide, cerium, cycloaddition, epoxides, pyrazolates

## Abstract

The homoleptic pyrazolate complexes [Ce^III^_4_(Me_2_pz)_12_] and [Ce^IV^(Me_2_pz)_4_]_2_ quantitatively insert CO_2_ to give [Ce^III^_4_(Me_2_pz⋅CO_2_)_12_] and [Ce^IV^(Me_2_pz⋅CO_2_)_4_], respectively (Me_2_pz=3,5‐dimethylpyrazolato). This process is reversible for both complexes, as observed by in situ IR and NMR spectroscopy in solution and by TGA in the solid state. By adjusting the molar ratio, one molecule of CO_2_ per [Ce^IV^(Me_2_pz)_4_] complex could be inserted to give trimetallic [Ce_3_(Me_2_pz)_9_(Me_2_pz⋅CO_2_)_3_(thf)]. Both the cerous and ceric insertion products catalyze the formation of cyclic carbonates from epoxides and CO_2_ under mild conditions. In the absence of epoxide, the ceric catalyst is prone to reduction by the co‐catalyst tetra‐*n*‐butylammonium bromide (TBAB).

## Graphical abstract

**CO_2_ sponges**: Cerous and ceric pyrazolates have been demonstrated to insert carbon dioxide and afford the complexes [Ce^III^_4_(Me_2_pz⋅CO_2_)_12_] and [Ce^IV^(Me_2_pz⋅CO_2_)_4_]. The insertion is reversible and can be exploited for the catalytic formation of cyclic carbonates from epoxides. The potentially reducing behavior of tetra‐*n*‐butylammonium bromide (TBAB) as a co‐catalyst is also revealed.

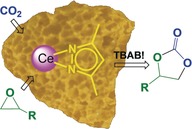

## Introduction

Inexorably rising CO_2_ levels in the earth's atmosphere—and their consequential environmental impact—have spurred much interest in combating CO_2_ build‐up.^1^ Capture technologies, such as carbon dioxide capture and storage (CCS) and direct air capture (DAC),^2^ and CO_2_ conversion into fuels or chemical feedstocks^3^ appear promising. However, such tactics suffer from either a lack of appropriate storage and transportation of CO_2_, or overcoming the high activation barrier of CO_2_.^4^ To date, the most effective sorbents for CCS/DAC are alkali‐metal/alkaline‐earth metal hydroxide solutions, inorganic salts (e.g. alkali‐metal carbonates),^1^, ^2^ or high‐surface supported polyamines (max. CO_2_ sorption capacity ca. 3 mmol g^−1^ at 1 bar)^5^ and magnesium‐based metal‐organic frameworks (ca. 35 wt % or 8 mmol CO_2_ g^−1^ at 1 bar).^6^

**Scheme 1:**
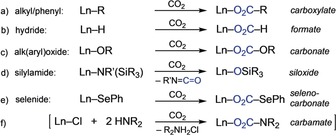
Irreversible reaction of carbon dioxide with archetypal organo‐rare‐earth‐metal complexes, with the exception of (e) as shown for (C_5_Me_5_)_2_Sm(SePh)(thf).^22^

Like alkaline‐earth metals, rare‐earth metals (Ln) feature a high affinity for carbon dioxide (cf. bastnaesite is the most important Ln^III^ deposit in the Earth's crust). Thus, metal‐organic derivatives easily react with or insert CO_2_, as initially demonstrated by Bochkarev et al. for homoleptic silylamides Ln[N(SiMe_3_)_2_]_3_ 7, 8 and alkoxides [Ln(O*n*Bu)_3_] (Scheme 1 c,d).^9^, ^10^

Similar archetypes (including Ln^III^ aryloxides) can also be used for chemical transformations, such as the catalytic conversion of a CO_2_/epoxide mixture into cyclic carbonates^11^ or copolymers.10a, 12, 13 However, highly reactive organo‐rare‐earth‐metal complexes such as alkyl^14^ and hydride10a, 15 (Scheme 1 a,b) or divalent derivatives^16^ display irreversible CO_2_ insertion or favor additional transformations through CO_2_ post‐activation (e.g. formation of CO, CO_3_^2−^, C_2_O_4_^2−^).^16^ Recently, cerium, the most abundant rare‐earth element, has gained attention for CO_2_ activation.^8^, ^17^, ^18^, ^19^ For example, while the hydrogen‐bonded Ce^IV^ oxo complex [(L_OEt_)_2_Ce=O(H_2_O)]⋅MeC(O)NH_2_ (L_OEt_^−^=[Co(η^5^‐C_5_H_5_){P(O)(OEt)_2_}_3_]^−^) was shown to form the tetravalent carbonate species [(L_OEt_)_2_Ce(CO_3_)],^17^ *ortho*‐NHC‐substituted aryloxide Ce^III^ complexes (NHC=N‐heterocyclic carbene) insert CO_2_ into the Ce−C_NHC_ bond in a semireversible manner, and catalytically form propylene carbonate from propylene oxide.^18^, ^19^

Bulky cyclopentadienyl (Cp) derivatives (e.g. Ln(C_5_Me_5_)_3_) were shown to accommodate CO_2_ insertion in a unidirectional manner, thereby forming very stable carboxylato moieties through a η^5^‐to‐η^1^ switch in the C_5_Me_5_ coordination (cf. Scheme 1 a).^20^ Pyrazolates (pz), on the other hand, are dinitrogen‐derived Cp counterparts, where the putative N−CO_2_ bond may tolerate a more reversible insertion process, as seen for other CO_2_‐heteroatom bonds (Scheme 1 e).^21^, ^22^ As the tetravalent [Ce(Me_2_pz)_4_]_2_ complex was recently shown to undergo reversible insertion of ketones into the Ce−N bond,^23^ we extended the study toward CO_2_. Quantitative insertion of CO_2_ into the Ce−N(Me_2_pz) bond was observed for both tetravalent and trivalent cerium Me_2_pz complexes, and intriguingly the insertion process was found to be fully reversible.

## Results and Discussion

**Scheme 2:**
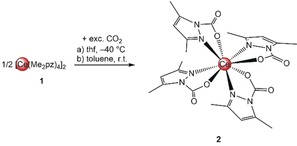
Insertion of CO_2_ into the Ce−N(Me_2_pz) bond of [Ce(Me_2_pz)_4_]_2_ at: a) −40 °C in thf or b) ambient temperature in toluene.

*Carbon Dioxide Insertion into a Ceric Pyrazolate*: Treatment of [Ce(Me_2_pz)_4_]_2_ (**1**) with excess CO_2_ in either toluene or thf (under 1 bar CO_2_ pressure) led to a color change from dark red to orange within 5 minutes (Scheme 2). Crystallization from concentrated toluene or thf solutions at −40 °C gave orange crystals of [Ce(Me_2_pz⋅CO_2_)_4_] with either toluene (**2⋅toluene**, 54 %) or thf (**2⋅thf**, 64 %) within the lattice. Discounting the lattice solvent, this accounts for about 25 wt % CO_2_ or 5.7 mmol CO_2_ per gram.

**Figure 1:**
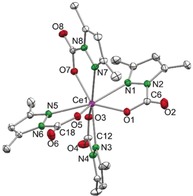
Crystal structure of [Ce(Me_2_pz⋅CO_2_)_4_] (**2⋅toluene**). Ellipsoids are shown at the 50 % probability level. Hydrogen atoms and lattice toluene are omitted for clarity. Selected bond lengths/angles are listed in the Supporting Information.

The molecular structure of **2⋅toluene** revealed an 8‐coordinate cerium(IV) center with four κ^2^(*N*,*O*)‐coordinating Me_2_pz⋅CO_2_ ligands (Figure 1), in contrast to the κ^2^(*O*,*O*) modes in carboxylates and related carbamates. The Ce−N and Ce−O bond lengths average 2.528 Å and 2.255 Å, respectively, thus matching the values found in the benzophenone‐inserted product [Ce(Me_2_pz)_2_(pdpm)_2_] (Ce1−N1 2.564 Å, Ce1−O1 2.173 Å; pdpm=(3,5‐dimethylpyrazol‐1‐yl)diphenylmethanolate).^23^ Other homoleptic Ce^IV^ complexes, [Ce(L)_4_] (with L as a donor‐functionalized alkoxy ligand engaged in a 5‐membered chelate to cerium), also have similar Ce−O bond lengths as those in **2**, thus highlighting a common chelating coordination motif.^24^, ^25^ Seemingly, no delocalization occurs across the O=C−O fragment, which exhibits average C−O bond lengths of 1.207 Å (terminal) and 1.291 Å (bridging). Support for the localization of the C−O double bond came from DRIFTS measurements of **2⋅toluene** and **2⋅thf**, which showed the presence of a strong absorption band at $\tilde{\nu }$ =1732 and 1718 cm^−1^, respectively, for the CO stretching of the C−O double bond as well as a strong absorption band at $\tilde{\nu }$ =1336 cm^−1^ for the CO stretching of the C−O single bond.

The structure was also supported by NMR spectroscopic measurements. The ^1^H NMR spectrum recorded in [D_8_]toluene at ambient temperature revealed two distinct methyl group environments for all the pyrazolato ligands, indicative of ligand asymmetry and complete consumption of [Ce(Me_2_pz)_4_]_2_. The ^13^C signal of the inserted CO_2_ was detected at *δ*=149.9 ppm, a region where pyrazolate N‐CO_2_R signals are expected.^26^ ^1^H NMR measurements on **2⋅thf** in [D_8_]THF at ambient temperature showed a mixture of products, which could not be assigned. Cooling the solution to −40 °C under 1 bar CO_2_ pressure led to a color change from red to orange and both the ^1^H and ^13^C NMR spectra recorded at −40 °C showed similar signals as **2⋅toluene** in [D_8_]toluene.

**Scheme 3:**
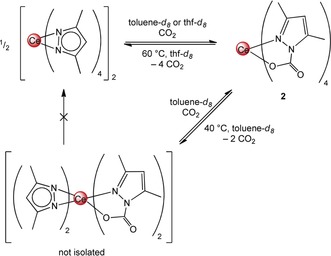
Reversible insertion of CO_2_ into the Ce−N(Me_2_pz) bond. The process is completely reversible in [D_8_]thf and partially reversible in [D_8_]toluene. The product arising from the elimination of CO_2_ in [D_8_]toluene has not been isolated; the structure is based on ^1^H NMR spectroscopic analysis at 40 °C.

Variable‐temperature (VT) NMR studies of **2⋅toluene** and **2⋅thf** in [D_8_]toluene and [D_8_]THF were conducted to investigate the reversibility of CO_2_ insertion (Scheme 3). In [D_8_]toluene, the formation of a new species at 40 °C was revealed and no further liberation of CO_2_ was observed even after heating above 60 °C (see Figure S6 in the Supporting Information). The ^1^H NMR spectrum recorded at 40 °C shows two sets of signals for different Me_2_pz moieties in a 1:1 ratio, which suggests the formation of putative compound [Ce(Me_2_pz)_2_(Me_2_pz⋅CO_2_)_2_].

**Figure 2:**
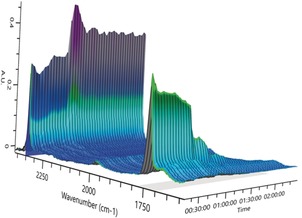
In situ IR spectroscopy of **2⋅thf** at 60 °C in the range of $\tilde{\nu }$ =1700 to 2350 cm^−1^. Normalized intensities are shown. Spectra were recorded every minute.

Recooling the solution did not reform **2⋅toluene** quantitatively, likely because some of the liberated CO_2_ was no longer within the reaction medium, but the addition of fresh CO_2_ quantitatively reformed **2⋅toluene**. The [D_8_]THF VT NMR experiment of compound **2⋅thf** showed a different CO_2_‐deinsertion behavior (see Figure S10). As a consequence of competitive thf coordination, displacement of CO_2_ starts at 10 °C and is complete at 60 °C, with formation of [Ce(Me_2_pz)_4_(thf)]. As seen in the experiment in [D_8_]toluene, this reaction is fully reversible by recooling the sample and subsequently introducing CO_2_. Additionally, in situ IR measurements were performed at 60 °C, which showed complete loss of inserted CO_2_ and formation of free CO_2_ (Figure 2 and see also Figure S58).

In the solid state, **2⋅toluene** is stable for several weeks at −40 °C, but at ambient temperature it partially loses CO_2_ over a few days or when it is exposed to vacuum, as indicated by a color change from orange to dark red. A thermogravimetric analysis (TGA), performed under a flow of Ar and heating the sample slowly from 28 °C to 250 °C, indicated an initial loss of mostly lattice toluene. The liberation of CO_2_ and small amounts of lattice toluene was dominant between 55 and 95 °C, as revealed by a step of 21.92 % (theoretical proportion of CO_2_ in **2⋅toluene** 19.98 %). At 250 °C, only nonvolatile parts of **2⋅toluene** remain, leaving a mass of 49.79 % of the initial weight (theoretical value 51.61 %; see Figure S59). Although the deinsertion of carbon dioxide was achieved in the solid state, bulk compound **1** did not insert any carbon dioxide when stored under 1 bar CO_2_ pressure for three days. Moreover, compound **1** was hydrolyzed upon exposure to air within one hour (DRIFT spectrum, see Figure S57).

**Figure 3:**
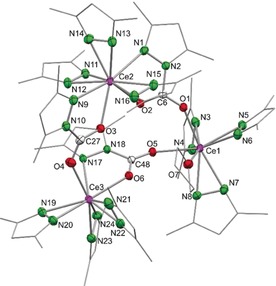
Crystal structure of [Ce_3_(Me_2_pz)_9_(Me_2_pz⋅CO_2_)_3_(thf)] (**3**). Ellipsoids are shown at the 50 % probability level. Hydrogen atoms and lattice *n*‐hexane are omitted for clarity. Selected bond lengths/angles are listed in the Supporting Information.

**Scheme 4:**
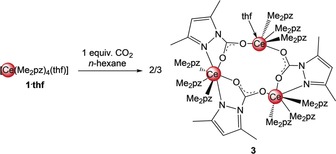
Insertion of one equivalent of CO_2_ into the Ce−N(Me_2_pz) bond of [Ce(Me_2_pz)_4_(thf)].

[Ce(Me_2_pz)_4_(thf)] (**1‐thf**) was treated with stoichiometric amounts of CO_2_ to generate the putative [Ce(Me_2_pz)_2_(Me_2_pz⋅CO_2_)_2_] (Scheme 3). Although this species could not be isolated, it was possible to generate monoinserted [Ce_3_(Me_2_pz)_9_(Me_2_pz⋅CO_2_)_3_(thf)] (**3**) in moderate yields of 46 % (Scheme 4). The crystal structure of ceric **3** shows a ring motif with two distinct 9‐coordinate and one 10‐coordinate cerium atoms (Figure 3). Although all the cerium centers are coordinated by three Me_2_pz ligands in an η^2^(*N*,*N*′) fashion, Ce1 connects further to two oxygen atoms of neighboring Me_2_pz⋅CO_2_ ligands as well as an additional thf molecule, 10‐coordinate Ce2 is surrounded by two κ^2^:(*N*,*O*)‐chelating Me_2_pz⋅CO_2_ ligands, and Ce3 exhibits additional contacts to one κ^2^:(*N*,*O*)‐Me_2_pz⋅CO_2_ ligand and an oxygen atom of a neighboring Me_2_pz⋅CO_2_ ligand.

Each Me_2_pz⋅CO_2_ ligand bridges between two cerium atoms. In contrast to homoleptic **2**, all the oxygen atoms are engaged in cerium bonding, which implies delocalized O−C−O bonds (av. C−O, 1.247 Å). The η^2^(*N*,*N*′)‐Ce−N(Me_2_pz) bond lengths are in the expected range.^24^, ^27^ According to VT NMR studies carried out in [D_8_]toluene, the trimetallic entity **3** is retained in solution at low temperatures, with every dimethylpyrazolato ligand showing a distinct signal set in the proton NMR spectrum at −80 °C (see Figure S12). The signals for the protons of the bridging Me_2_pz⋅CO_2_ ligands are shifted upfield compared to those of the terminal η^2^(*N*,*N′*)‐Me_2_pz ligands. As a consequence of the equilibrium between potential alternative oligomers formed in the presence of only one equivalent of CO_2_ per cerium, the interpretation of the ambient‐temperature NMR spectrum was difficult. This was already experienced for the insertion of benzophenone into the Ce−N(Me_2_pz) bond.^23^ Upon heating to 90 °C, CO_2_ was liberated and [Ce(Me_2_pz)_4_] re‐formed (see Figure S14). After cooling to ambient temperature, a partial reinsertion of CO_2_ was observed, as was found in the VT NMR experiments on **2⋅toluene** and **2⋅thf**.

**Figure 4:**
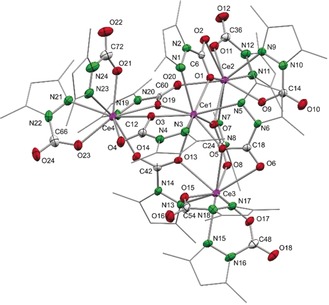
Crystal structure of [Ce_4_(Me_2_pz⋅CO_2_)_12_] (**5**). Ellipsoids are shown at the 50 % probability level. Hydrogen atoms and lattice toluene (ten molecules) are omitted for clarity. Cutouts of the crystal structure of **5** and a schematic view of different Me_2_Pz⋅CO_2_ binding modes, as well as selected bond lengths/angles are shown in the Supporting Information.

**Scheme 5:**
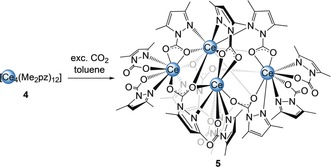
Insertion of of CO_2_ into the Ce−N(Me_2_pz) bonds of cerous [Ce_4_(Me_2_pz)_12_].

*Carbon Dioxide Insertion into a Cerous Pyrazolate*: To examine the role of the oxidation state of cerium (Ce^IV^ versus Ce^III^) and, therefore, the impact of its Lewis acidity, cerous donor‐free [Ce_4_(Me_2_pz)_12_]^28^ (**4**) was used as a precursor for CO_2_ insertion (Scheme 5). Remarkably, while retaining the Ce_4_ nuclearity, all of the Ce−N(Me_2_pz) moieties engaged in CO_2_ insertion, leading to the complex [Ce_4_(Me_2_pz⋅CO_2_)_12_] (**5**; Figure 4). Compared to the six different coordination modes of the pyrazolato moieties in starting material **4**,^28^ the crystal structure of **5** revealed only three. Ce2, Ce3, and Ce4 form a nearly equilateral triangle bridged by μ_3_‐1κ^2^(*N*,*O*):2κ(*O*):3κ(*O*′)‐Me_2_pz⋅CO_2_. Each of the 9‐coordinate cerium centers (Ce2, Ce3, and Ce4) is also surrounded by two terminal Me_2_pz⋅CO_2_ groups in a κ^2^(*N*,*O*) coordination mode and two oxygen atoms in a κ^2^(*O*,*O*′) fashion (Figure S66). This triangle is capped by 9‐coordinate Ce1, which is coordinated to three Me_2_pz⋅CO_2_ ligands in a κ^2^(*N*,*O*) manner (Ce1−N_avg_ 2.710 Å, Ce1−O_avg_ 2.395 Å) and to three bridging oxygen atoms. The Ce−O bond lengths are similar to those found in [Ce(L^R^⋅CO_2_)_3_] (L^R^=2‐*O*‐3,5‐*t*Bu_2_‐C_6_H_2_(1‐C{N(CH)_2_N(R)}) and R=*i*Pr and Mes) reported by Arnold et al. with Ce−O bond lengths of 2.466(6)–2.482(6) Å.18b The O−C−O bond lengths indicate delocalization of the bridging Me_2_pz⋅CO_2_ ligands, with two C−O bond lengths in the same region (1.235(9)–1.264(9) Å) and rather localized C−O single (1.26(1)–1.301(9) Å) and C−O double bonds (1.21(1)–1.229(9) Å) for the capping and terminal Me_2_pz⋅CO_2_ ligands. For further comparison, the cerous carbamate [Ce_4_(O_2_CN*i*Pr_2_)_12_] features a lozenged arrangement of one 8‐coordinate and three 7‐coordinate Ce^III^ centers with Ce−O bond lengths in the range 2.322(7)–2.746(7)  Å (no Ce‐N interaction).^29^ The latter complex was obtained from the reaction of CeCl_3_(DME) with HN*i*Pr_2_ and CO_2_ (Scheme 1f). In accordance with the crystal structure of cluster **5**, DRIFTS measurements show strong absorption bands for both the C−O single bonds ($\tilde{\nu }$ =1250–1350 cm^−1^) and C−O double bonds ($\tilde{\nu }$ =1600–1750 cm^−1^). ^1^H DOSY NMR measurements on **5** in [D_8_]toluene, [D_8_]THF, or a [D_8_]toluene/3,3‐dimethyl‐1,2‐butylene oxide mixture revealed distinct diffusion coefficients (see Figures S17–S20) for the solvents employed and only one additional peak corresponding to a much larger species but correlating with every other signal in the proton NMR spectra. Calculation of the molar mass of this compound ([D_8_]toluene: *M_r_*=1989 g mol^−1^; [D_8_]THF: *M_r_*=1643 g mol^−1^; [D_8_]toluene + 3,3‐dimethyl‐1,2‐butyleneoxide: *M_r_*=2357 g mol^−1^) as a compact sphere‐like molecule^30^ suggests it exists as a tetrametallic (*M_r_*=2242 g mol^−1^) or a non‐monometallic species in solution. Treating [Ce(Me_2_pz)_3_(thf)]_2_ with CO_2_ in [D_8_]THF gave the same NMR spectrum as that of **5⋅toluene**, thus indicating the formation of a multimetallic compound also in donor solvents (see Figure S16). TGA of **5⋅toluene** also showed an initial loss of toluene (cf. **2⋅toluene**), followed by a pronounced step (21.39 % weight loss) in the range from 52 to 90 °C, consistent with the release of CO_2_ (theoretical value: 16.75 %) and some lattice toluene (Figure S60). At 250 °C, only the nonvolatile parts of **5⋅toluene** remain and a total loss of 46.82 wt % compared to the starting material fits well with the theoretical value of 45.99 % for 10 molecules of toluene and 12 molecules of CO_2_ eliminated from [Ce_4_(Me_2_pz⋅CO_2_)_12_]⋅10 toluene (**5⋅toluene**).

*Catalytic Formation of Cyclic Carbonates from CO_2_ and Oxiranes*: Having established the efficiency and reversibility of CO_2_ insertion into Ce−N(Me_2_pz) bonds, we were interested in any catalytic utilization. Accordingly, pyrazolate complexes **1** and **4** were probed as catalysts for the generation of cyclic carbonates from CO_2_ and oxiranes. In the absence of CO_2_, compound **1** interacts with epoxides, as indicated by a noticeable shift in the ^1^H NMR spectrum upon addition of one equivalent of 3,3‐dimethyl‐1,2‐butylene oxide (see Figure S24). This is most likely due to formation of a donor adduct, which is considered a crucial step in Lewis‐acid‐catalyzed cycloaddition reactions. Even though such donor adducts were shown to be isolable (e.g. Tp^*t*Bu^Ca(O‐2,6‐*i*Pr_2_C_6_H_3_)⋅(PO); Tp^*t*Bu^=tris(3‐*t*Bu‐pyrazolyl)borato, PO=propylene oxide^31^), the putative [Ce(Me_2_pz)_4_(PO)] (**1‐PO**) could not be isolated. Tetra‐*n*‐butylammonium bromide (TBAB) was employed as a co‐catalyst, since it was shown to promote the highest activities in such cycloaddition reactions.11b, 32 The reaction was optimized for propylene oxide, which gave almost quantitative conversion after 24 h under mild conditions. Using 0.5 mol % **1** or 0.25 mol % **4** and 1 mol % TBAB without solvent at ambient temperature and 1 bar CO_2_ pressure gave 93 % conversion for the tetravalent catalyst **1** and 98 % for its trivalent counterpart (Table 1, entries 1 and 3). The CO_2_‐insertion complexes **2** and **5** displayed similar catalytic activity (entries 2 and 4). The conversion dropped drastically on increasing the steric bulk of the substituent on the epoxides. As a result, styrene oxide, and 3,3‐dimethyl‐1,2‐butylene oxide showed only very low conversions (entry 21) for ceric **1** and almost no conversion in the case of trivalent catalyst **4** (entry 22). Moderate conversion was observed for 1,2‐*n*‐hexylene oxide with the tetravalent catalyst **1** (entry 13). Conducting the catalysis at higher temperature increased the TONs with both catalysts **1** and **4**, and resulted in nearly quantitative conversion for both systems (entries 15 and 16). Without co‐catalyst TBAB, **1** showed moderate catalytic activity at 90 °C (entry 17). In almost all cases, tetravalent **1** showed higher catalytic activity than cerous **4**, which most likely results from the higher Lewis acidity of Ce^IV^ versus Ce^III^. To further evaluate the catalytic reaction with catalyst **1** and propylene oxide, the TOFs at different stages of the catalysis were determined (see Table S1). After a short induction period, most likely corresponding to the insertion of CO_2_ into **1**, the TOF reached a maximum of 11 h^−1^ within the first 3 h. For comparison, a TOF of 155 h^−1^ was reported by Yao and co‐workers when performing the reaction under 10 bar CO_2_ pressure.11a Increasing the CO_2_ pressure did not significantly affect the catalytic activity of compound **1** (entries  13 vs. 18 and 21 vs. 23). However, a simultaneous increase of the temperature to 90 °C and the CO_2_ pressure to 10 bar led to a marked improvement in the catalytic activity, resulting in TONs of up to 300 for the sterically demanding 3,3‐dimethyl‐1,2‐butylene oxide (entries 24 and 26). The latter conditions were also applicable for the cycloaddition of CO_2_ and cyclohexene oxide, an internal epoxide (entries 27–29). Having optimized the reaction conditions, we determined the initial turnover frequencies for the different epoxides (entries 5, 12, 20, and 25). As expected, the TOFs increased as the steric of the substituents bulk decreased, ranging from 24 to 196 h^−1^ and giving almost quantitative conversion of propylene oxide after a reaction time of one hour (entry 5). Compared to the other catalyst systems based on rare‐earth metals reported by Yao and co‐workers (TOFs up to 440 h^−1^) or by Otero and co‐workers (3167 h^−1^), our system shows only moderate catalytic activity under comparable conditions.11a, 11b

**Table 1:** Catalytic formation of cyclic carbonates from epoxides and CO_2_.^[a]^

Entry	Catalyst	Substrate	Product	Conversion [%]	TON/Ce
1	**1**	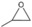	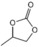	93	93
2	**2**			93	93
3	**4**			98	98
4	**5**			96	96
5	**1** ^[i]^			98	196
					
6	**1**	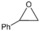	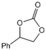	20	20
7	**1** ^[b]^			4	8
8	**4**			13	13
9	**4** ^[c]^			6	12
10	**4⋅thf**			13	13
11	**4⋅thf** ^[c]^			7	14
12	**1** ^[i]^			12	24
					
13	**1**	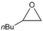	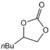	61	61
14	**4**			25	25
15	**1** ^[d]^			98	98
16	**4** ^[e]^			96	96
17	**1** ^[f]^			25	25
18	**1** ^[g]^			76	76
19	**6**			3	3
20	**1** ^[i]^			49	98
					
21	**1**	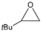	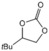	12	12
22	**4**			3	3
23	**1** ^[g]^			9	9
24	**1** ^[h]^			84	168
25	**1** ^[i]^			14	28
26	**1** ^[j]^			60	300
					
27	**1**	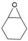	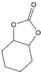	2	2
28	**1** ^[h]^			77	154
29	**1** ^[j]^			35	175

[a] Reaction conditions if not otherwise noted: 1 bar CO_2_ pressure and 0.5 mol % catalyst (1 mol % for **2** and **6**; 0.25 mol % for **4** and **5**) and 1 mol % co‐catalyst for 24 h at ambient temperature in neat epoxide. [b] 0.25 mol % catalyst **1** and 0.5 mol % TBAB, 24 h. [c] 0.125 mol % catalyst **4** and 0.5 mol % TBAB, 24 h. [d] 0.5 mol % catalyst **1** and 1 mol % TBAB, 24 h, 90 °C. [e] 0.25 mol % catalyst **4** and 1 mol % TBAB, 24 h, 90 °C. [f] At 90 °C without TBAB as a co‐catalyst. [g] 0.5 mol % catalyst **1** and 1 mol % TBAB, 24 h, 10 bar CO_2_ pressure. [h] 0.25 mol % catalyst **1** and 0.5 mol % TBAB, 24 h, 90 °C, 10 bar CO_2_ pressure. [i] 0.25 mol % catalyst **1** and 0.5 mol % TBAB, 1 h, 90 °C, 10 bar CO_2_ pressure. [j] 0.1 mol % catalyst **1** and 0.2 mol % TBAB, 24 h, 90 °C, 10 bar CO_2_ pressure.

**Scheme 6:**
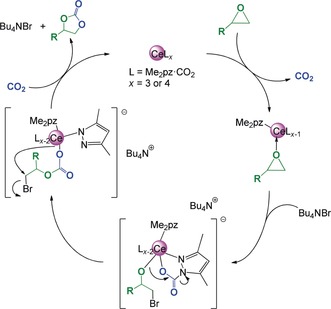
Proposed overall mechanism for the coupling of CO_2_ with epoxides.

The mechanism of the cycloaddition of CO_2_ and epoxides using tetraalkylammonium salts as co‐catalysts has been discussed in detail.^32^, ^33^ It is generally accepted that the epoxide is activated by coordination to a Lewis‐acidic metal center followed by a nucleophilic ring‐opening attack of the bromide to form a metal‐alkoxy bond (Scheme 6). Subsequently, the alkoxide reacts with CO_2_ and cyclizes to produce a cyclic carbonate. Hints that the mechanisms for the cycloaddition differ using ceric **1** or cerous **4** as the catalyst could be found when conducting the reactions with different amounts of catalyst loading (entries 7 and 9). This results in a change in the TONs for the tetravalent catalyst **1**, whereas the TONs remained the same for trivalent complex **4**. The occurrence of distinct reaction mechanisms is not surprising, as **1** is a monometallic complex while **4** is a tetrametallic compound in the solid state and in solution (for a more detailed possible mechanism see Scheme S1). Such a mechanism, involving multiple metal centers, was previously proposed for the bimetallic complex [Al(salen)]_2_O by North and co‐workers.^32^ However, any detailed information about the mechanism could not be retrieved from our catalyst system as the TOFs decreased enormously when the reactions were conducted in propylene carbonate (no significant conversion at ambient temperature after 24 h and ca. 10 % conversion after 20 h at 90 °C) or any other solvent, which makes kinetic studies unfeasible.

Complex **2** underwent a reductive side reaction in the presence of TBAB to afford cerous species [Ce(Me_2_pz⋅CO_2_)_4_][NBu_4_] (**6**). This side reaction occurred during the catalytic studies and when combining **1** and TBAB in stoichiometric amounts in [D_8_]toluene under 1 bar CO_2_ pressure. Although **2** is very stable in toluene solution under these conditions (no color change was observed after several days), it underwent reduction in the presence of TBAB within hours, as evidenced by decolorization of the orange solution. Most probably bromine is formed as an oxidation product; however, no brominated product could be detected in the reaction mixture (see Figure S23). The use of epoxides as a solvent seems to stabilize the tetravalent **2**, as no paramagnetic signals were found in the ^1^H NMR spectra of the catalytic reactions. The crystal structure of **6** revealed the same motif as seen in **2⋅toluene** and **2⋅thf** (see Figure S67). The 8‐coordinate cerium center bears four κ^2^(*N*,*O*) Me_2_pz⋅CO_2_ ligands with elongated Ce−N and Ce−O bonds compared to ceric **2⋅toluene** and **2⋅thf**, as would be expected for a cerium(III) center. Cerous **6** displays poor catalytic activity compared to tetravalent **1** (Table 1, entry 19), thus underlining that it is a side product and not the active catalyst.

## Conclusion

We have shown that carbon dioxide easily inserts into the Ce−N(Me_2_pz) bond of both ceric [Ce(Me_2_pz)_4_]_2_ and cerous [Ce_4_(Me_2_pz)_12_] at an amount equivalent to 5.7 mmol CO_2_ per gram complex and via the controlled activation of 12 molecules of CO_2_ within one complex, respectively. The insertion process is reversible both in solution and in the solid state, with CO_2_ desorption being complete at <100 °C. Both trivalent and tetravalent cerium pyrazolate complexes are active catalysts for the cycloaddition of epoxides and carbon dioxide with TBAB as a co‐catalyst under mild conditions. We are currently investigating the carbon dioxide capture performance of silica‐grafted variants of Ce‐pyrazolates,^34^ and our findings might also stimulate research in the area of cerium‐dipyrazolate‐based CO_2_‐“breathable”/expandable MOFs.^35^, ^36^

## Conflict of interest

The authors declare no conflict of interest.

## Supporting information

As a service to our authors and readers, this journal provides supporting information supplied by the authors. Such materials are peer reviewed and may be re‐organized for online delivery, but are not copy‐edited or typeset. Technical support issues arising from supporting information (other than missing files) should be addressed to the authors.

Supplementary
