# Supplementary material for: Effective and Reversible Carbon Dioxide Insertion into Cerium Pyrazolates
Source: Angew Chem Int Ed Engl. 2020 Jan 30;59(14):5830–6. doi: 10.1002/anie.201916483 (PMC7155069; doi:10.1002/anie.201916483)
Supplement: Supplementary file 1 — Supplementary [file ANIE-59-5830-s001.pdf]

Angewandte Chemie
Eine Zeitschrift der Gesellschaft Deutscher Chemiker

Supporting Information

Effective and Reversible Carbon Dioxide Insertion into Cerium Pyrazolates

*Uwe Bayer, Daniel Werner, Cäcilia Maichle-Mössmer, and Reiner Anwander**

anie_201916483_sm_miscellaneous_information.pdf

Supporting Information

Table of Contents

Experimental Section including Catalysis	**S3**
NMR Spectra	**S7**
IR Spectra	**S35**
Thermogravimetric Analysis	**S38**
Crystallographic Data	**S40**
References	**S48**

Experimental Section

General Procedures. All manipulations were performed under an inert atmosphere (Ar) using a glovebox (*MBraun* 200B; <0.1 ppm O_2, <0.1 ppm H_2O), or according to standard Schlenk techniques in oven-dried glassware. The solvents were purified with Grubbs columns (MBraun SPS, solvent purification system) and stored in a glovebox. [Ce(Me_2pz)$_4$]$_2$, [Ce(Me_2pz)$_3$(thf)]$_2$ and [Ce(Me_2pz)$_3$]$_4$ were synthesized according to published procedures.[1-3] Cyclohexane was pre-dried over NaK alloy. Benzene-*d*$_6$ (C_6D_6), toluene-*d*$_8$, and thf-*d*$_8$ were purchased from *Euriso-top*, pre-dried over NaK alloy, and filtered off prior use, thf-*d*$_8$ was re-condensed. Chloroform-*d3* was purchased from *Euriso-top* and used as received. NMR spectra were recorded at 26 °C with either a Bruker AVII+400 (^{1}H: 400.13 MHz, ^{13}C: 100.16 MHz), a Bruker DRX-250 (^{1}H: 250.00 MHz, ^{13}C: 62.86 MHz) or a Bruker-Avance II 500 (^{1}H: 500.13 MHz, ^{13}C: 125.76 MHz) using J. Young valve NMR spectroscopy tubes. ^{1}H and ^{13}C NMR shifts are referenced to a solvent resonance and reported in parts per million (ppm) relative to tetramethylsilane.[4] Analyses of NMR spectra were performed with ACD/NMR Processor Academic Edition (product version: 12.01). Infrared spectra were recorded on a *ThermoFisher Scientific* NICOLET 6700 FTIR ($\tilde{\nu}$= 4000 – 400 cm^{-1}) spectrometer using a DRIFTS chamber with dry KBr/sample mixtures and KBr windows. Elemental analysis (C, H, N) was performed on an *Elementar vario MICRO cube*. *In situ* IR spectra were recorded on a METTLER TOLEDO ReactIR 15. Thermogravimetric analyses (DTA) were performed under argon flow (60 l min^{-1}) heating from ambient temperature to 250 °C at a rate of 0.5 K min^{-1} in corundum crucibles on a Netzsch STA 449 F3 Jupiter.

Synthesis

[Ce(Me_2pz)$_4$(thf)] (1-thf). [Ce{N(SiHMe$_2$)$_2$}$_4$] (46.9 mg, 0.079 mmol) was dissolved in cyclohexane (2 mL), and added to a THF (~0.5 mL) solution of Me_2pzH (26.5 mg, 0.28 mmol). The mixture was shaken for 30 seconds before immediate exposure to vacuum, giving a bright red powder of crude [Ce(Me_2pz)$_4$(thf)] (**2a**). Crystallization from *n*-hexane yielded red blocks of **2a** (25 mg, 62%). ^{1}H NMR (26 °C, 250.00 MHz, C_6D_6): δ = 6.13 (s, 4 H, CH), 3.31 (m, 4 H, α-CH thf), 2.34 (s, 24 H, CH_3), 0.94 (m, 4 H, β-CH thf) ppm; ^{13}C NMR (26 °C, 62.86 MHz, C_6D_6): δ = 144.6 (*C*CH_3), 112.6 (CH), 70.3 (α-CH thf), 25.2 (β-CH thf), 13.5 (CH_3) ppm; IR (DRIFT): ν = 3099 (w), 3024 (w), 3013 (s), 2942 (s), 2878 (s), 2859 (m), 1517 (vs), 1474 (w), 1470 (w), 1455 (w), 1444 (m), 1432 (vs), 1417 (s), 1364 (m), 1315 (w), 1301 (w), 1106 (w), 1050 (w), 1028 (w), 1006 (m), 959 (w), 921 (w), 871 (m), 806 (w), 781 (w), 728 (w) cm^{-1}; elemental analysis calcd. (%) for $C_{24}H_{36}CeN_8O$ (592.71 g mol^{-1}, performed on crystalline **2a**): C 48.63, H 6.12, N 18.91; found: C 47.89, H 5.78, N 19.37.

[Ce(Me_2pz·CO_2)$_4$]·2 toluene (**2·toluene**). [Ce(Me_2pz)$_4$]$_2$ (**1**) (0.043 g, 0.042 mmol) was dissolved in toluene (2 mL) and stirred under 1 bar CO_2 pressure. After 5 min the dark red solution turned orange and was stored at −40 °C. After 1 day the supernatant solution was separated and the crystalline orange material was dried with a paper towel and subsequently under reduced pressure at −40 °C, leaving [Ce(Me_2pz·CO_2)$_4$]·2 toluene (**2·toluene**) as orange crystals. Yield 0.040 g (0.045 mmol, 54%). ^{1}H NMR (26 °C, 400.13 MHz, toluene-*d*$_8$) δ: 5.09 (4 H, s, CH), 2.81 (12 H, s, CH_3), 2.04 (12 H, s, C*H*$_3$) ppm. ^{13}C{^{1}H} NMR (26 °C, 100.16 MHz toluene-*d*$_8$) δ: 152.7 (*C*CH_3), 149.9 (OCO), 144.2 (*C*CH_3), 109.9 (CH), 14.0 (CH_3), 12.6 (*C*H$_3$) ppm. DRIFTS: $\tilde{\nu}$ = 3129

(vw), 3093 (vw), 2979 (vw), 2931 (vw), 1788 (vw), 1732 (vs), 1558 (m), 1496 (vw), 1457 (m), 1416 (w), 1385 (w), 1336 (s), 1292 (m), 1258 (s), 1213 (w), 1164 (w), 1156 (w), 1122 (s), 1042 (m), 983 (w), 838 (m), 817 (w), 778 (w), 769 (w), 741 (vw), 455 (m), 409 (vw) cm^{-1}. Elemental analysis calc. (%) $C_{38}H_{44}CeN_8O_8$ (880.93 g mol^{-1}) C 51.81, H 5.03, N 12.72; found C 51.85, H 4.71, N 12.79.

[Ce(Me$_2$pz·CO$_2$)$_4$]·2 thf (**2·thf**). [Ce(Me$_2$pz)$_4$]$_2$ (**1**) (0.254 g, 0.244 mmol) was dissolved in thf (2 mL) and stirred under 1 bar CO_2 pressure at 0 °C. After 5 min the dark red solution turned orange and was stored at −40 °C. After 1 day the supernatant solution was separated and the crystalline orange material was dried with a paper towel and subsequently at −40 °C under reduced pressure, leaving [Ce(Me$_2$pz·CO$_2$)$_4$]·2 thf (**2·thf**) as orange crystals. Yield 0.264 g (0.313 mmol, 64%). ^{1}H NMR (−20 °C, 500.13 MHz, thf-*d$_8$*) δ: 6.09 (4 H, s, CH), 2.63 (12 H, s, CH_3), 2.40 (12 H, s, CH_3) ppm. ^{13}C{^{1}H} NMR (−20 °C, 125.76 MHz, thf-*d$_8$*) δ: 152.4 (CCH_3), 152.4 (*C*CH3), 150.2 (OCO), 144.4 (*C*CH3), 110.2 (CH), 13.8 (CH3), 13.2 (CH_3) ppm. DRIFTS: $\tilde{\nu}$ = 3138 (vw), 2975 (w), 2865 (w), 1792 (vw), 1718 (vs), 1559 (m), 1458 (m), 1414 (m), 1379 (w), 1336 (s), 1293 (m), 1255 (m), 1168 (w), 1045 (m), 986 (w), 912 (vw), 822 (m), 777 (w), 768 (w), 628 (vw), 457 (m) cm^{-1}. Elemental analysis calc. (%) $C_{32}H_{44}CeN_8O_{10}$ (840.87 g mol^{-1}) C 45.70, H 5.27, N 13.33; found C 45.29, H 5.11, N 13.66.

[Ce$_3$(Me$_2$pz)$_9$(Me$_2$pzCO$_2$)$_3$(thf)] (**3**). [Ce(Me$_2$pz)$_4$(thf)] (0.050 g, 0.084 mmol) was stirred in *n*-hexane (3 mL), and CO_2 (2.1 mL, 0.084 mmol) was admitted. The reaction was stirred for 5 min at ambient temperature and then stored at −35 °C. After 2 days the supernatant solution was removed and the crystals were allowed to evaporate to dryness in the atmosphere of the glovebox, leaving [Ce$_3$(Me$_2$Pz)$_{9(}$Me$_2$pz·CO$_2$)$_3$(thf)] as dark red crystals. Yield: 0.023 g (0.013 mmol, 46%). ^{1}H NMR (−80 °C, 400.13 MHz, toluene-*d$_8$)* δ: 6.30 (s, 1H, C4-H Me$_2$pz), 6.29 (s, 1H, C4-H Me$_2$pz), 6.27 (s, 1H, C4-H Me$_2$pz), 6.24 (s, 1H, C4-H Me$_2$pz), 6.22 (s, 1H, C4-H Me$_2$pz), 6.20 (s, 1H, C4-H Me$_2$pz), 6.12 (s, 1H, C4-H Me$_2$pz), 6.04 (s, 1H, C4-H Me$_2$pz), 5.97 (s, 1H, C4-H Me$_2$pz), 5.26 (s, 1H, C4-H Me$_2$pzCO$_2$), 5.24 (s, 1H, C4-H Me$_2$pzCO$_2$), 5.15 (s, 1H, C4-H Me$_2$pzCO$_2$), 3.91 (bs, 2H, α-CH thf), 3.69 (bs, 2H, α-CH thf), 2.53, 2.43, 2.39, 2.33, 2.21, 2.04, 1.97, 1.91, 1.83, 1.82, 1.28, 1.25, 1.21, 1.18 ppm. Signals 1.18 – 2.53 ppm show singlets for CH_3 Me$_2$pz and β-CH thf. Elemental analysis calc. (%) for $C_{67}H_{92}Ce_3N_{24}O_7$ (1764.47 g mol^{-1}) C 45.57, H 5.25, N 19.04, found C 45.45, H 5.26, N 19.28.

[Ce$_4$(Me$_2$pz·CO$_2$)$_{12}$]·10 toluene (**5·toluene**). [Ce$_4$(Me$_2$pz)$_{12}$] (**4**) (0.285 g, 0.167 mmol) was dissolved in toluene (5 mL) and stirred under 1 bar CO_2 pressure at ambient temperature. After 5 min the light yellow solution turned colorless and was stored at −40 °C. After 3 d the supernatant solution was separated and the crystalline orange material was dried with a paper towel and subsequently at −40 °C under reduced pressure, leaving [Ce$_4$(Me$_2$pz·CO$_2$)$_{12}$]·10 toluene (**5·toluene**) as colorless crystals. Yield 0.430 g (0.136 mmol, 82 %).^{1}H NMR (26 °C, 400.13 MHz, toluene-*d$_8$*) δ: 6.42 (s), 6.30 (s), 5.83 (s), 4.16 (s), 1.84 (s), 1.61 (s), 0.50 (s), 0.27 (s), −1.32 (s), −2.39 (s), −3.00 (s), −5.03 (s), −7.15 (s), ppm. DRIFTS: $\tilde{\nu}$ = 2976 (vw), 2926 (vw), 1725 (vs), 1683 (vs), 1570 (w), 1558 (w), 1516 (vw), 1466 (m), 1416 (m), 1378 (vs), 1347 (vs), 1286 (s), 1206 (w), 1157 (vw), 1127 (m), 1037 (w), 980 (w), 862 (w), 824 (w), 791 (w), 759 (w), 731 (vw) cm^{-1}. Elemental analysis calc. (%) for $C_{142}H_{164}Ce_4N_{24}O_{24}$ (3151.48 g mol^{-1}) C 54.12, H 5.25, N 10.67 found C 52.08, H 4.82, N 11.55. Low carbon and hydrogen values as well as the high nitrogen value indicate loss of toluene.

Attempted synthesis of [Ce(Me_2pz·CO_2)$_4$][NBu_4] (6). [Ce(Me_2pz)$_4$]$_2$ (**1**) (0.096 g, 0.096 mmol) was dissolved in toluene (10 mL) and tetra-*n*-butylammonium bromide (TBAB) (61.8 mg, 0.192 mmol) was added and stored under 1 bar CO_2 pressure for 16 h. The dark red solution turned yellow and a yellow precipitate formed. The supernatant solution was separated and the yellow residue was dried at −40 °C under reduced pressure. Yield 0.103 g. Analysis of the yellow powder revealed a poorly fitting elemental anaylsis and an inconclusive ^{1}H NMR spectrum (Figure SX). Elemental analysis calc. (%) for $C_{40}H_{64}CeN_9O_8$ (939.12 g mol^{-1}) C 51.16, H 6.87, N 13.42 found C 51.73, H 6.53, N 11.22.

Catalytic studies

General procedure for the synthesis of cyclic carbonates for [Ce(Me_2pz)$_4$]$_2$ (1)

[Ce(Me_2pz)$_4$]$_2$ (**1**, 10.0 mg, 9.60 *μ*mol) and tetra-*n*-butylammonium bromide (TBAB) (6.20 mg, 19.2 μmol) were dissolved in 1.92 mmol of epoxide and stirred under 1 bar CO_2 pressure. After 24 h the mixture was dissolved in chloroform-*d* to determine the conversion of epoxide to cyclic carbonate *via* ^{1}H NMR spectroscopy.

General procedure for the synthesis of cyclic carbonates for [Ce_4(Me_2pz)$_{12}$] (4)

[Ce_4(Me_2pz)$_{12}$] (**4**, 6.1 mg, 14.3 *μ*mol) and tetra-*n*-butylammonium bromide (TBAB) (4.60 mg, 14.3 μmol) were dissolved in 1.43 mmol of epoxide and stirred under 1 bar CO_2 pressure. After 24 h the mixture was dissolved in chloroform-*d* to determine the conversion of epoxide to cyclic carbonate *via* ^{1}H NMR spectroscopy.

Table S1. TOFs for complex **1** under 1 bar CO_2 pressure[a]

Entry	Time [h]	Conversion [%]	TOF [h^{-1}]
1	1/12	0	0
2	1/2	4	8
3	1	10	10
4	2	21	10.5
5	3	33	11
6	4	38	9.5
7	12	79	6.6
8	18	86	4.8
9	24	93	3.9

[a] Reaction conditions: 1 bar CO_2 pressure and 0.5 mol% of catalyst **1** and 1 mol% of cocatalyst TBAB at ambient temperature in neat propylene oxide.

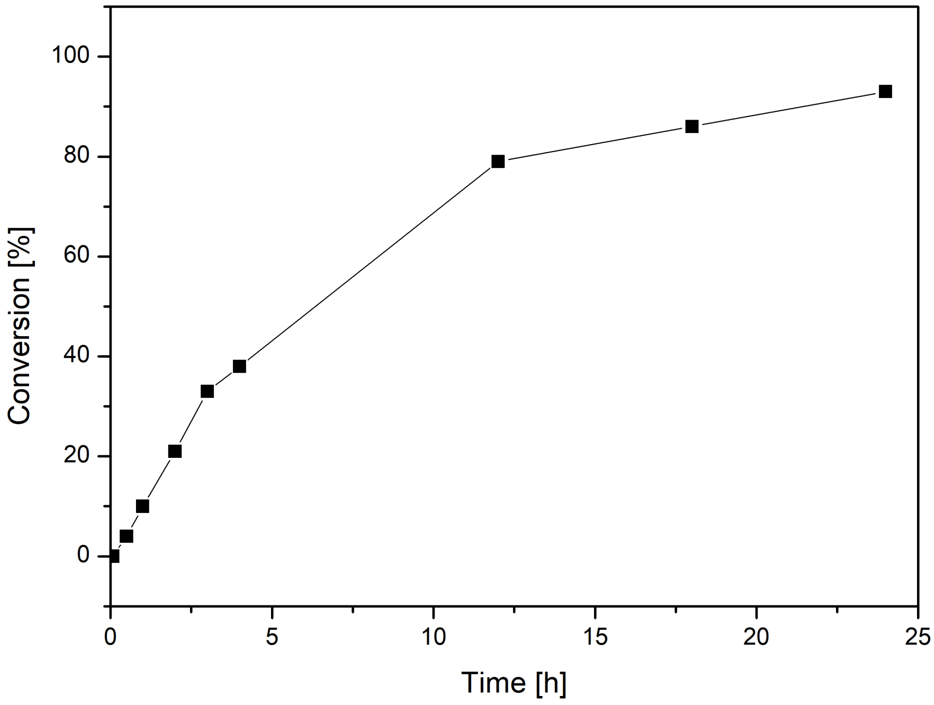

Figure S1. Conversion of propylene oxide and CO_2 catalyzed by **1** and TBAB at 1 bar CO_2 pressure plotted against the reaction time.

NMR Spectra (solvent signals are marked with *)

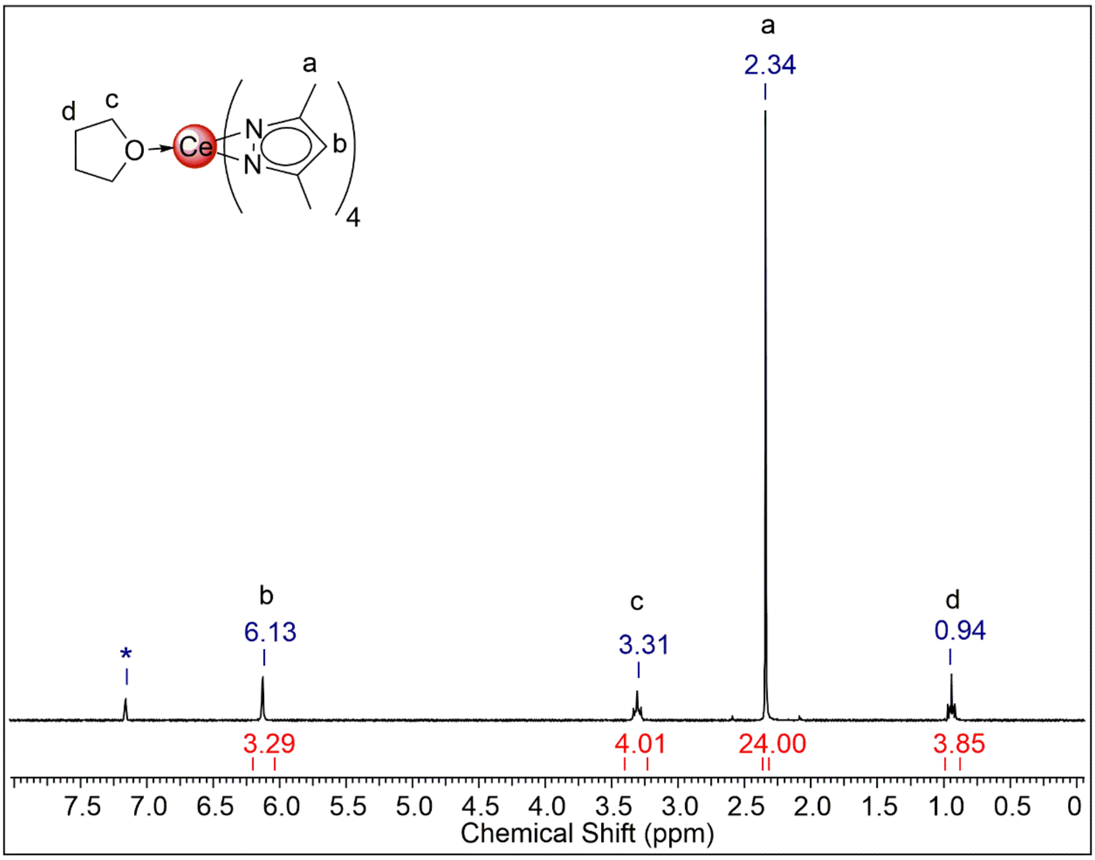

Figure S2. ^{1}H NMR spectrum (26 °C, 250.00 MHz, C_6D_6) of [Ce(Me_2pz)$_4$(thf)] (**1-thf**).

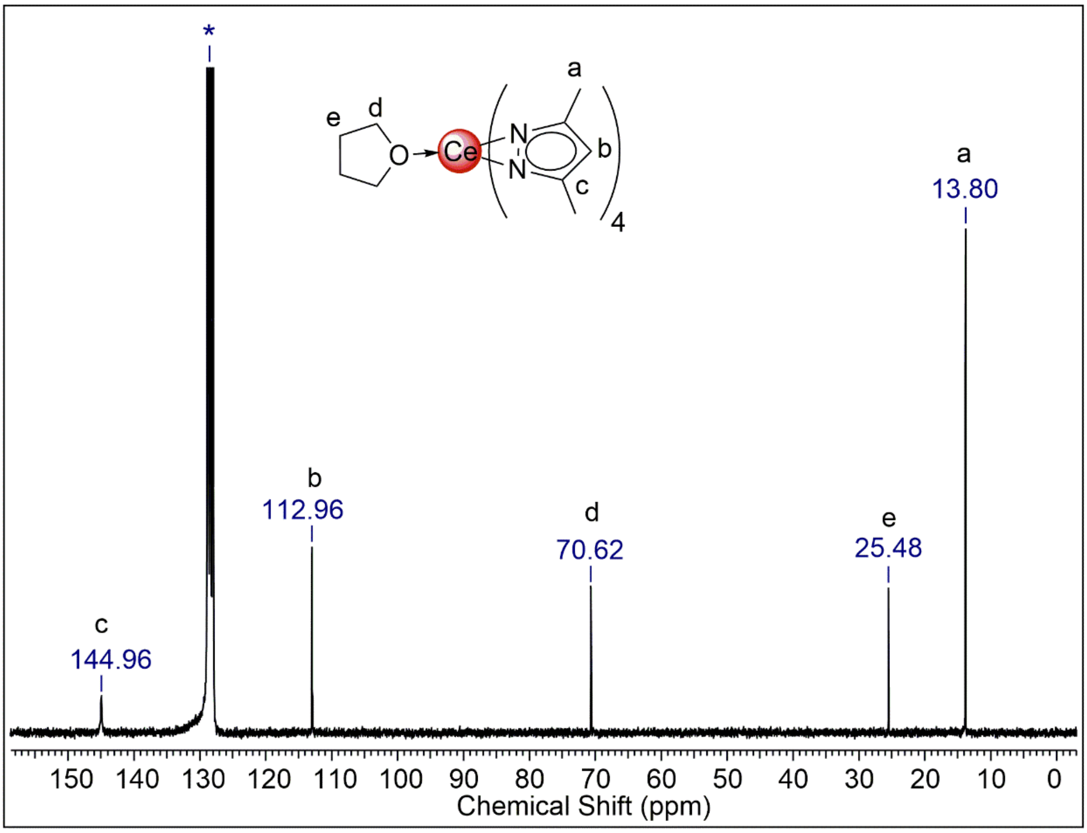

Figure S3. ^{13}C NMR spectrum (26 °C, 62.86 MHz, C_6D_6) of [Ce(Me_2pz)$_4$(thf)] (**1-thf**).

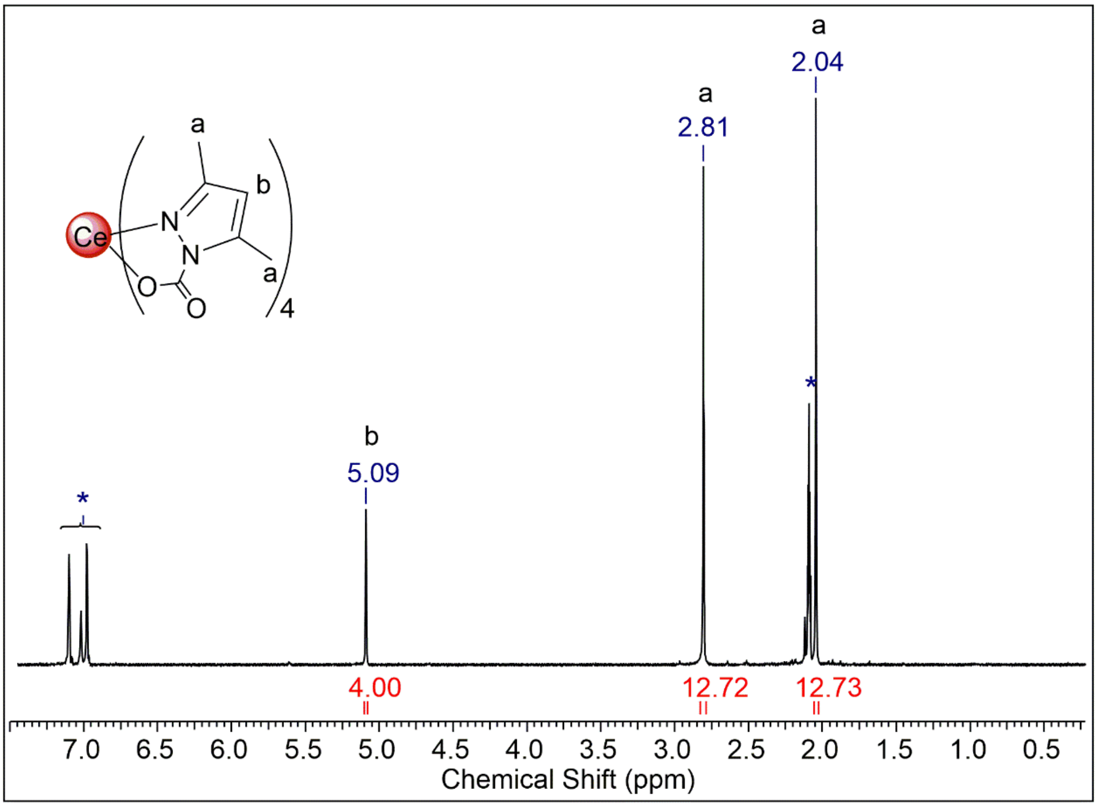

Figure S4. ^{1}H NMR spectrum (26 °C, 400.13 MHz, toluene-d_8) of [Ce(Me$_2$pz·CO$_2$)$_4$]·2 toluene (**2·toluene**).

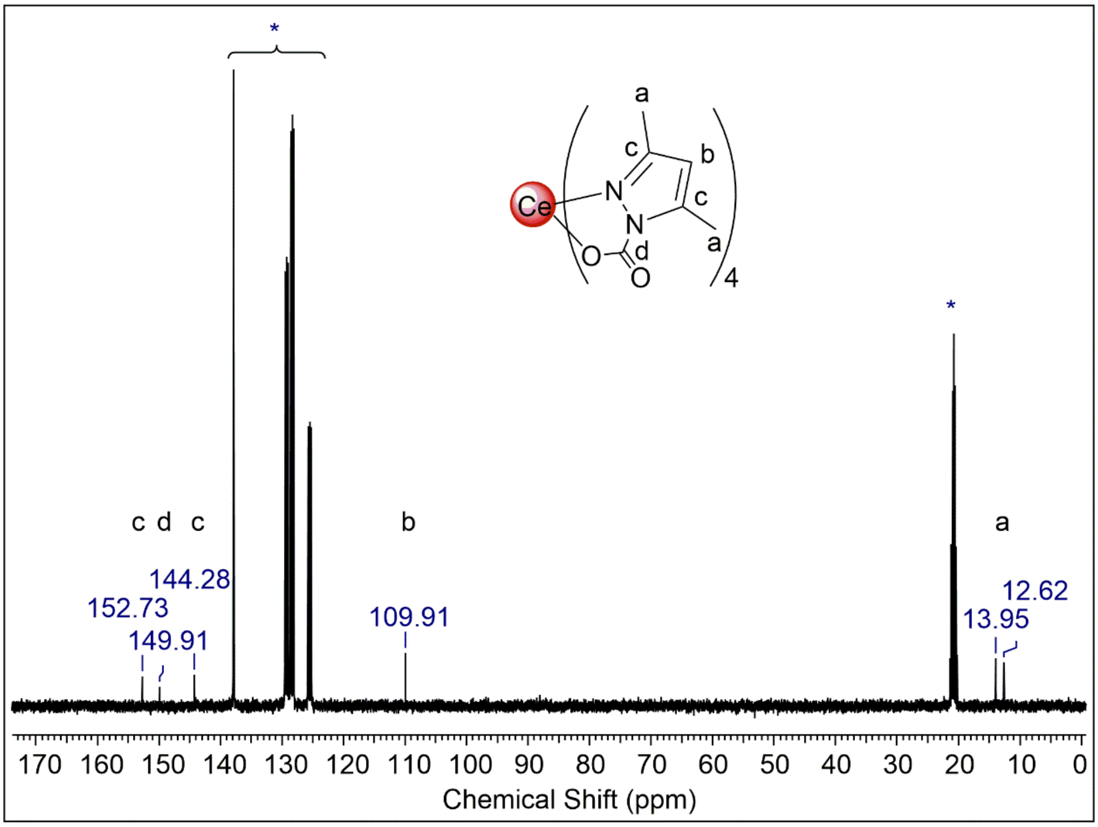

Figure S5. $^{13}C\{^{1}H\}$ NMR spectrum (26 °C, 100.16 MHz, toluene-d_8) of [Ce(Me$_2$pz·CO$_2$)$_4$]·2 toluene (**2·toluene**).

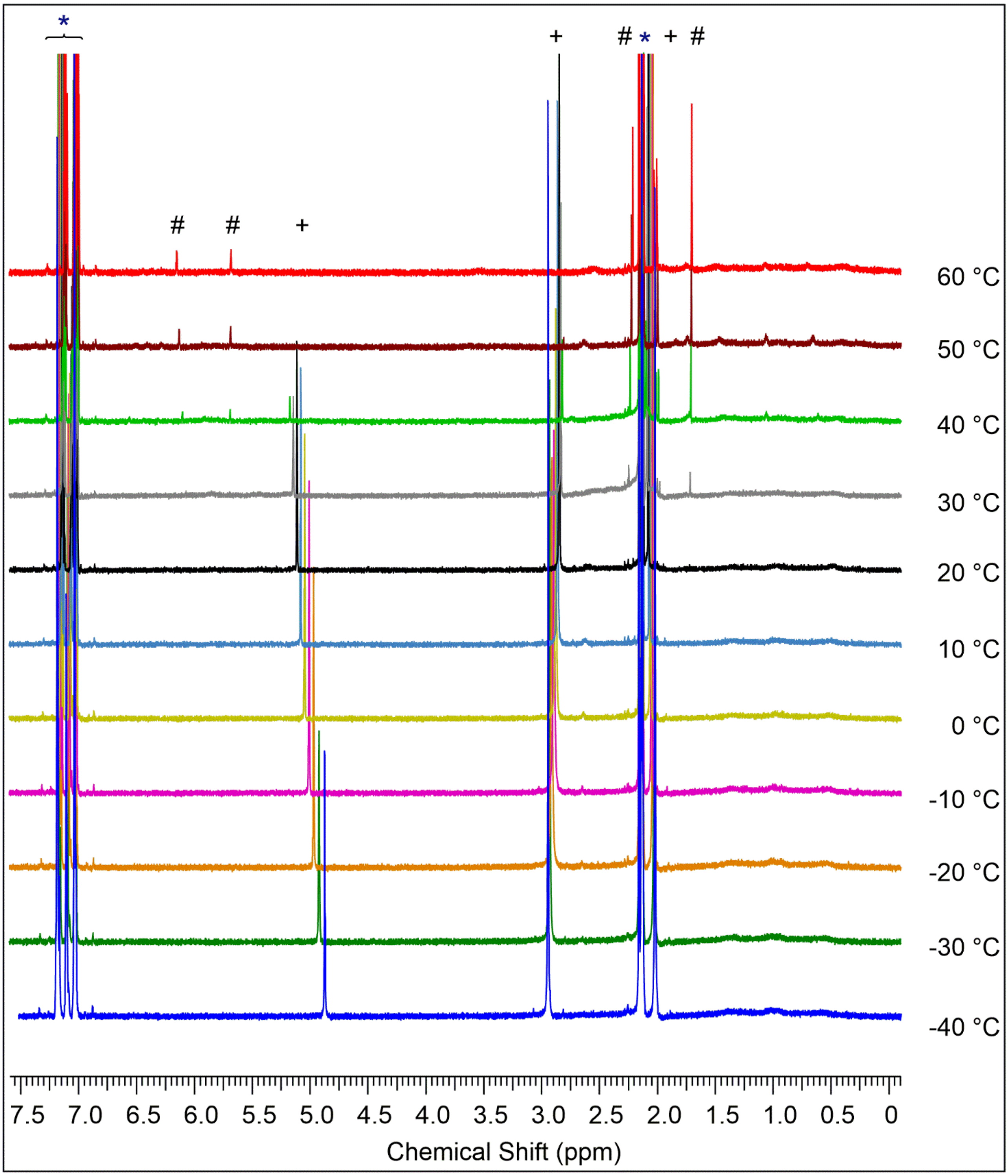

Figure S6. VT ^{1}H NMR spectra (500.13 MHz, toluene-d_8) of $[Ce(Me_2pz{\cdot}CO_2)_4]{\cdot}2$ toluene (**2·tolene**) in the range from −40 to 60 °C. Signals for **2·toluene** are marked with + and putative $[Ce(Me_2pz)_2(Me_2pz{\cdot}CO_2)_2]$ with #.

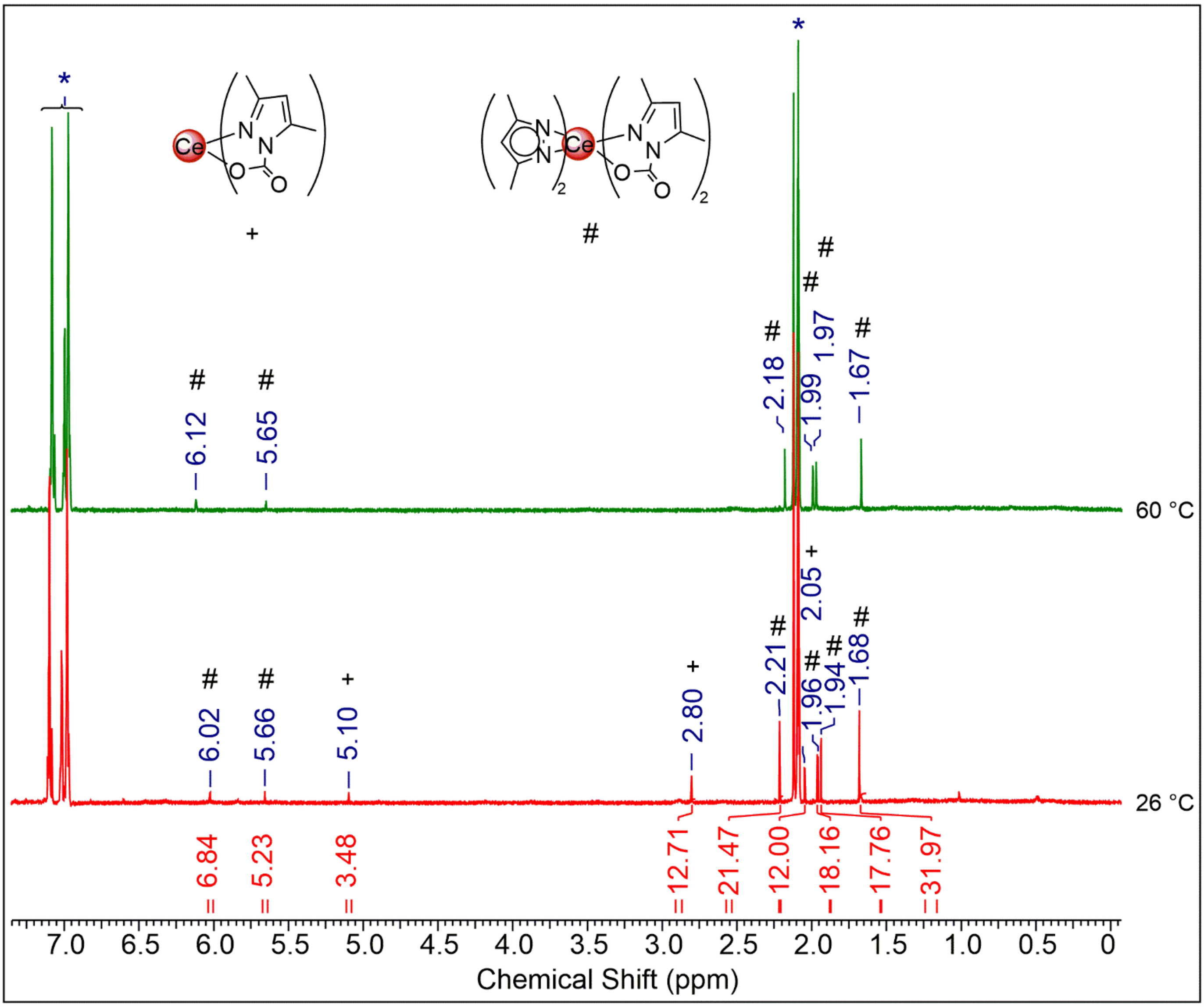

Figure S7. ^{1}H NMR spectra of $[Ce(Me_2pz{\cdot}CO_2)_4]{\cdot}2$ toluene (**2·toluene**) in toluene-d_8 at 60 °C and after cooling to 26 °C. Signals for **2·toluene** are marked with + and putative $[Ce(Me_2pz)_2(Me_2pz{\cdot}CO_2)_2]$ with #.

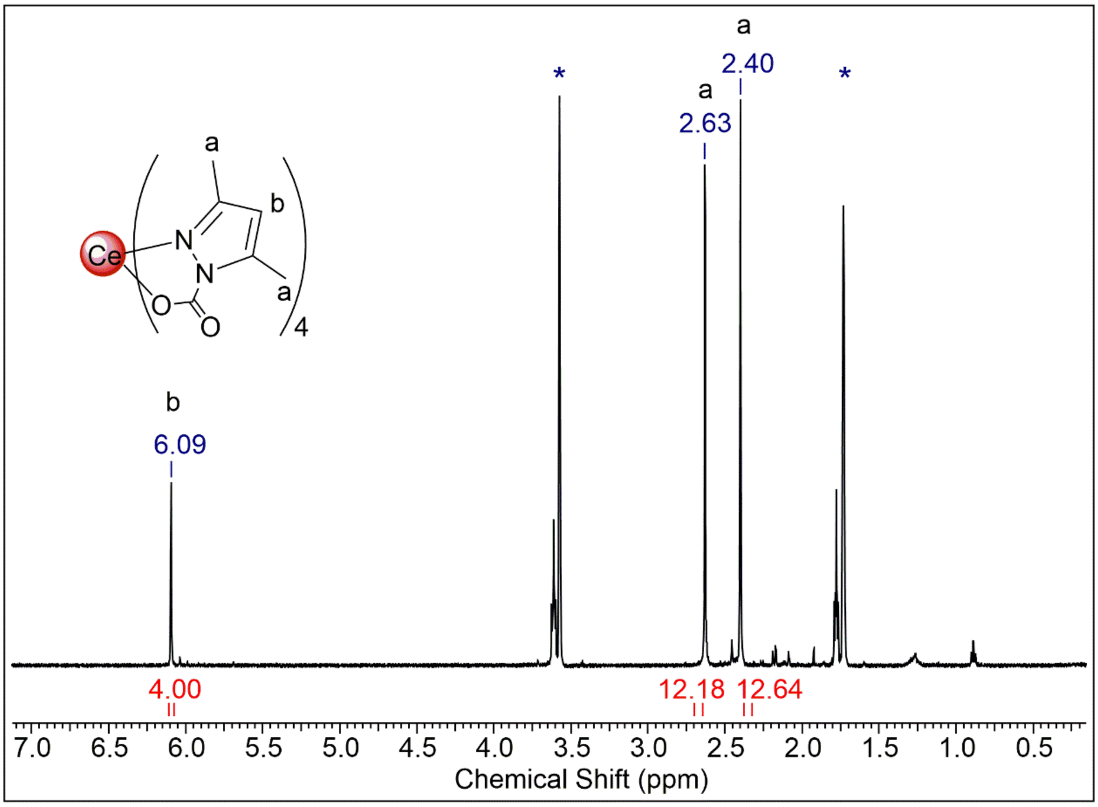

Figure S8. ^{1}H NMR spectrum (−20 °C, 500.13 MHz, thf-d_8) of [Ce($Me_2pz{\cdot}CO_2$)$_4$]·2 thf (**2·thf**).

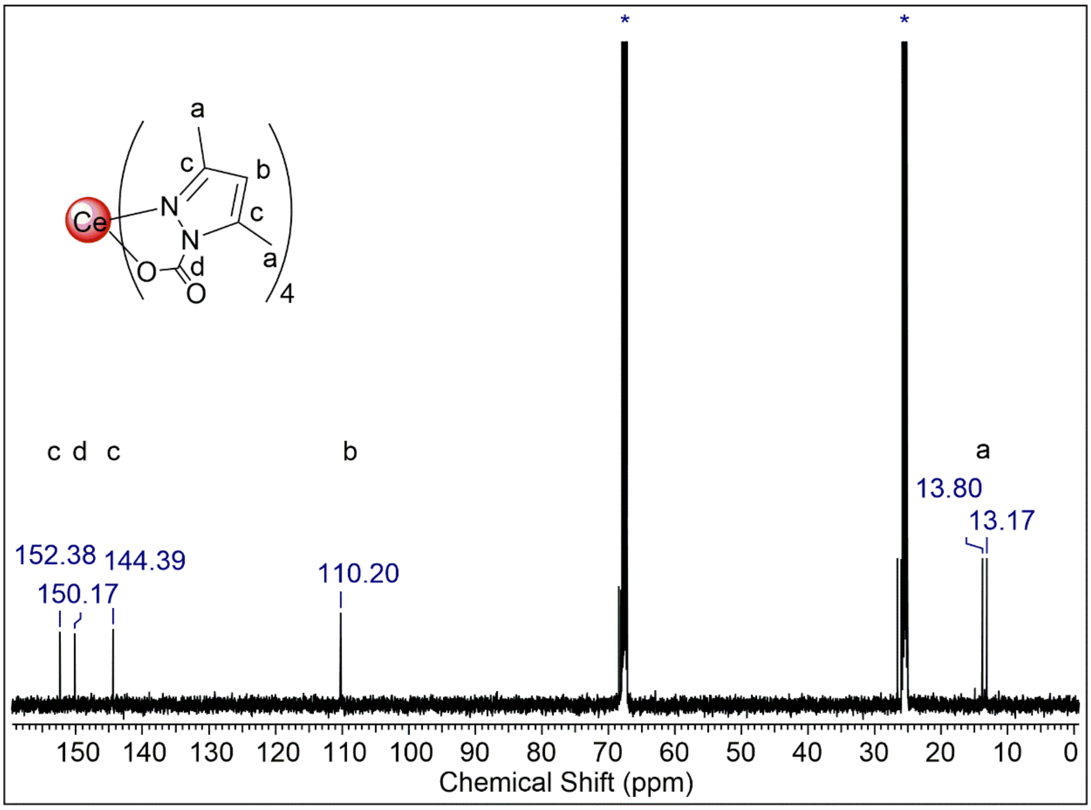

Figure S9. $^{13}C\{^{1}H\}$ NMR spectrum (−20 °C, 100.16 MHz, thf-d_8) of [Ce($Me_2pz{\cdot}CO_2$)$_4$]·2 thf (**2·thf**).

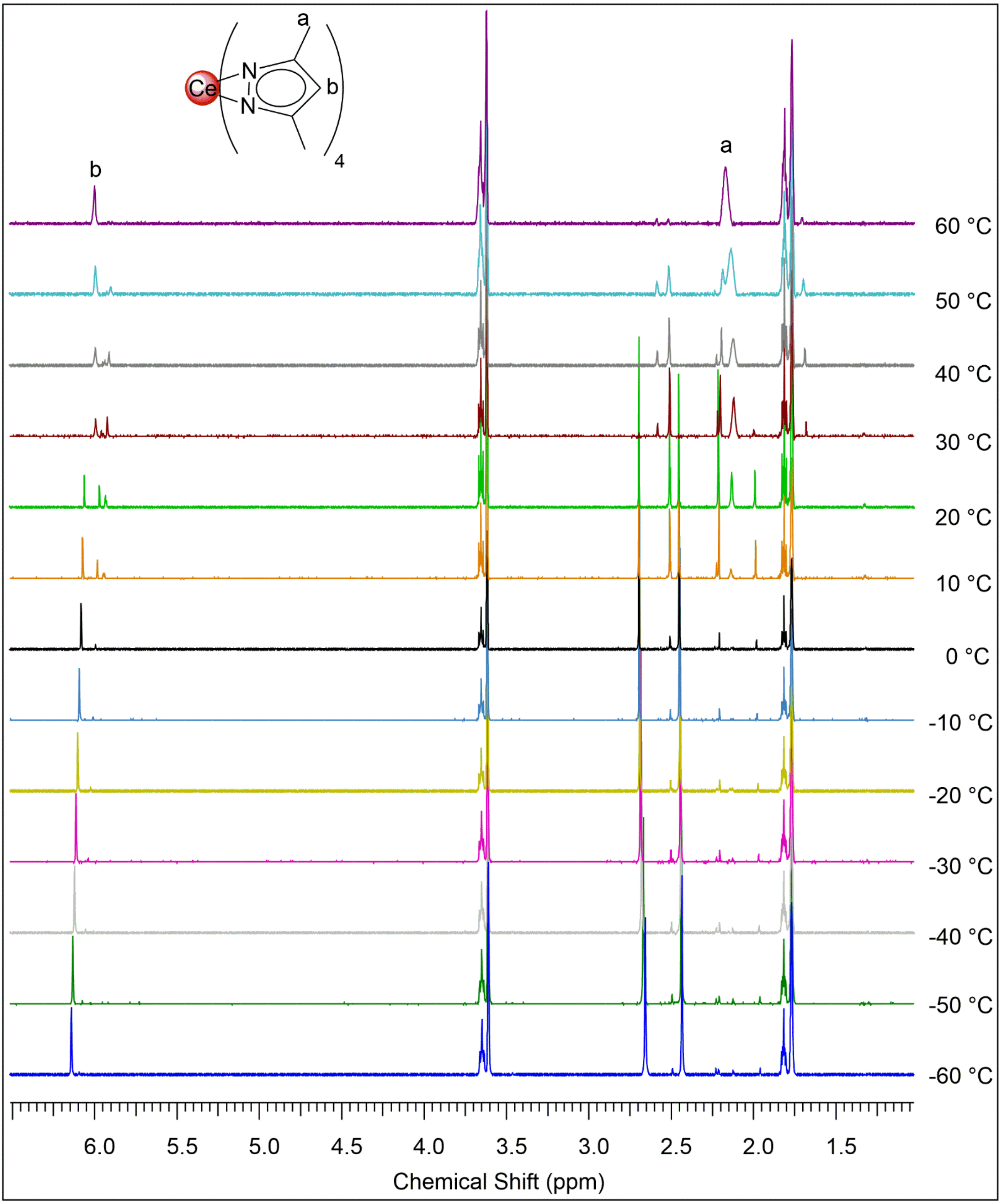

Figure S10. VT ^{1}H NMR spectra (500.13 MHz, thf-d_8) of [Ce($Me_2pz\cdot CO_2$)$_4$]·2 thf (**2·thf**) in the range from −60 to 60 °C showing the formation of [Ce(Me_2pz)$_4$]$_2$ (**1**) at 60 °C.

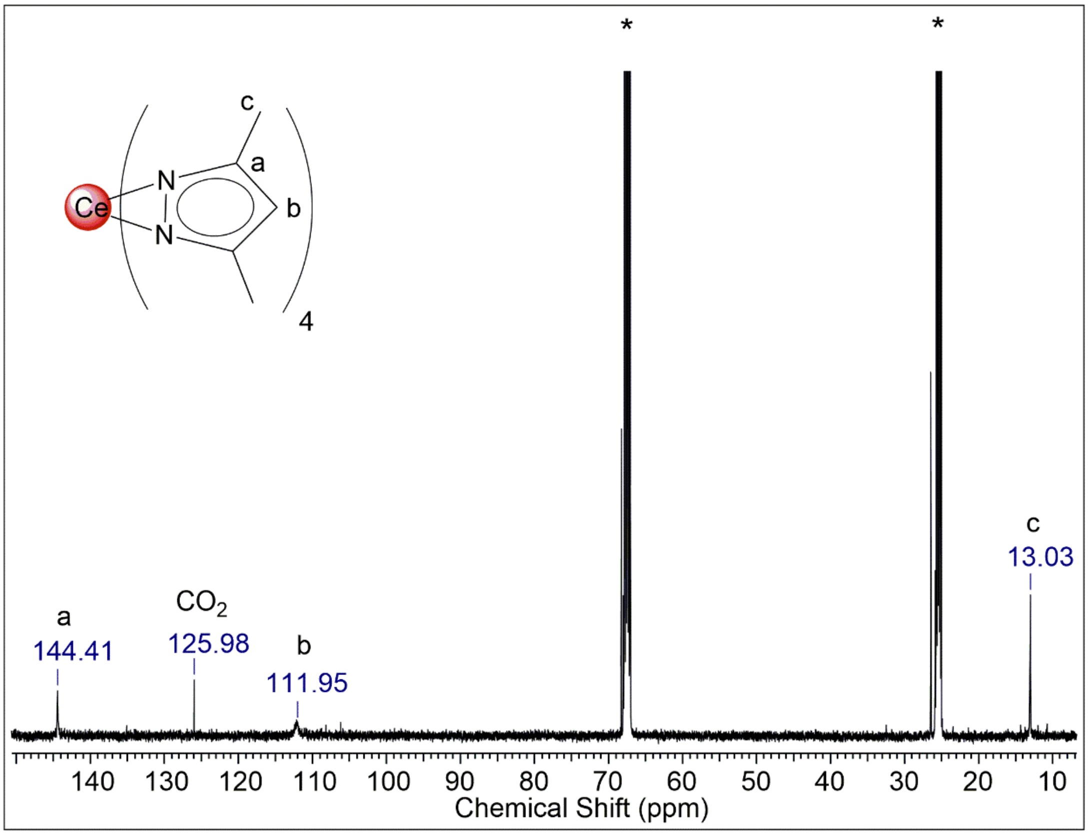

Figure S11. $^{13}C\{^{1}H\}$ NMR spectrum (60 °C, 100.16 MHz, thf-d_8) of [Ce($Me_2pz\cdot CO_2$)$_4$]·2 thf (**2·thf**).

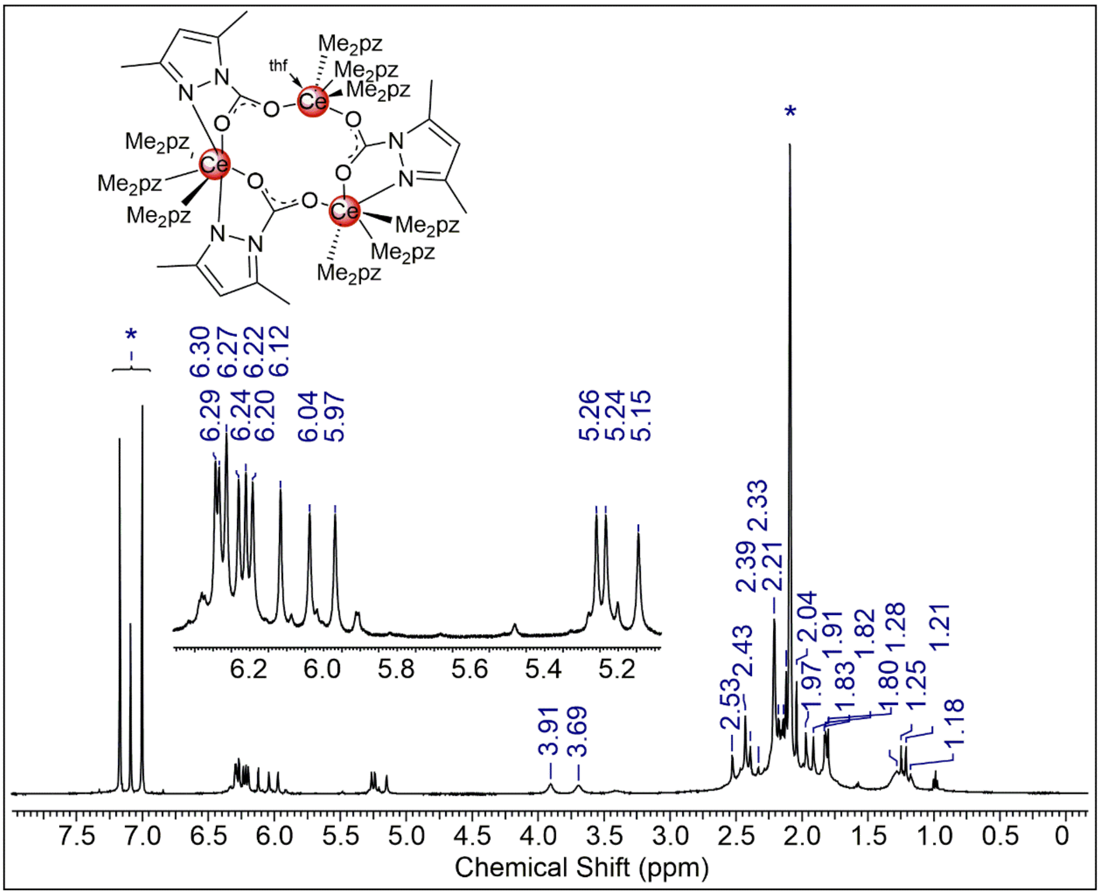

Figure S12. ^1H NMR spectrum (−80 °C, 500.13 MHz, toluene-d_8) of [$Ce_3(Me_2pz)_9(Me_2pz\cdot CO_2)_3$(thf)] (**3**).

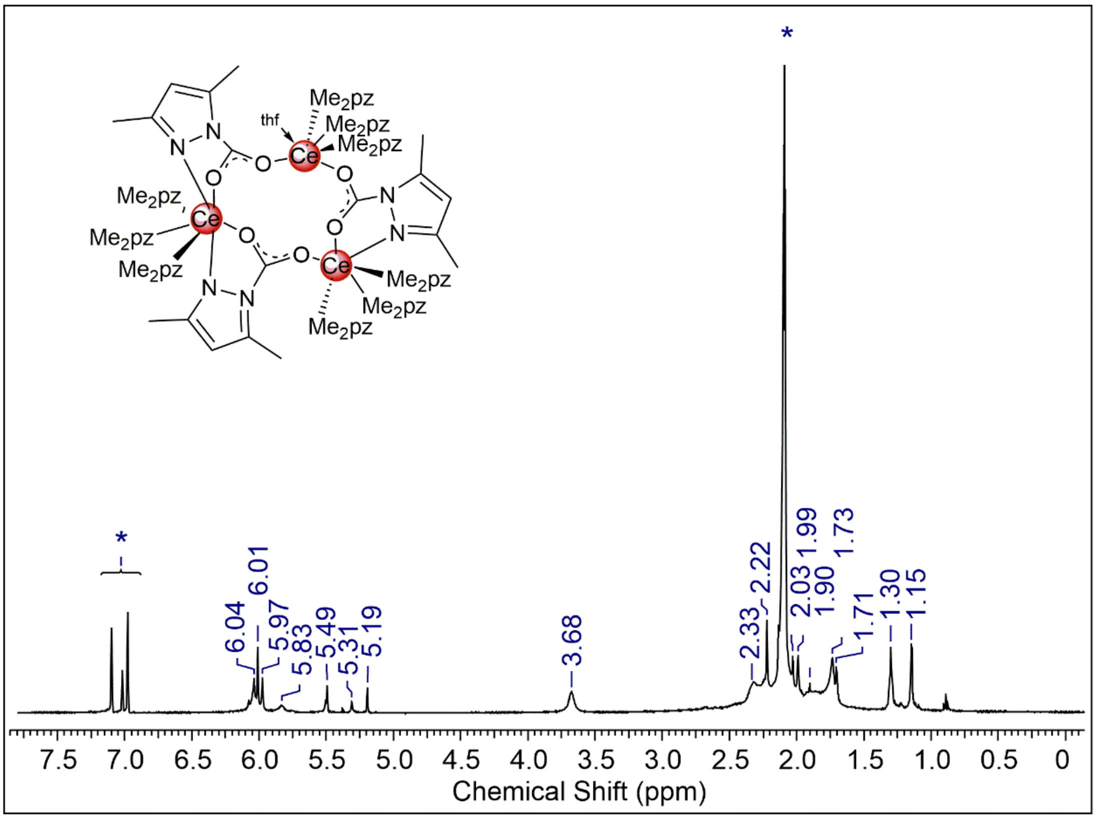

Figure S13. ^{1}H NMR spectrum (26 °C, 500.13 MHz, toluene-*d*$_8$) of [$Ce_3(Me_2pz)_9(Me_2pz{\cdot}CO_2)_3$(thf)] (**3**).

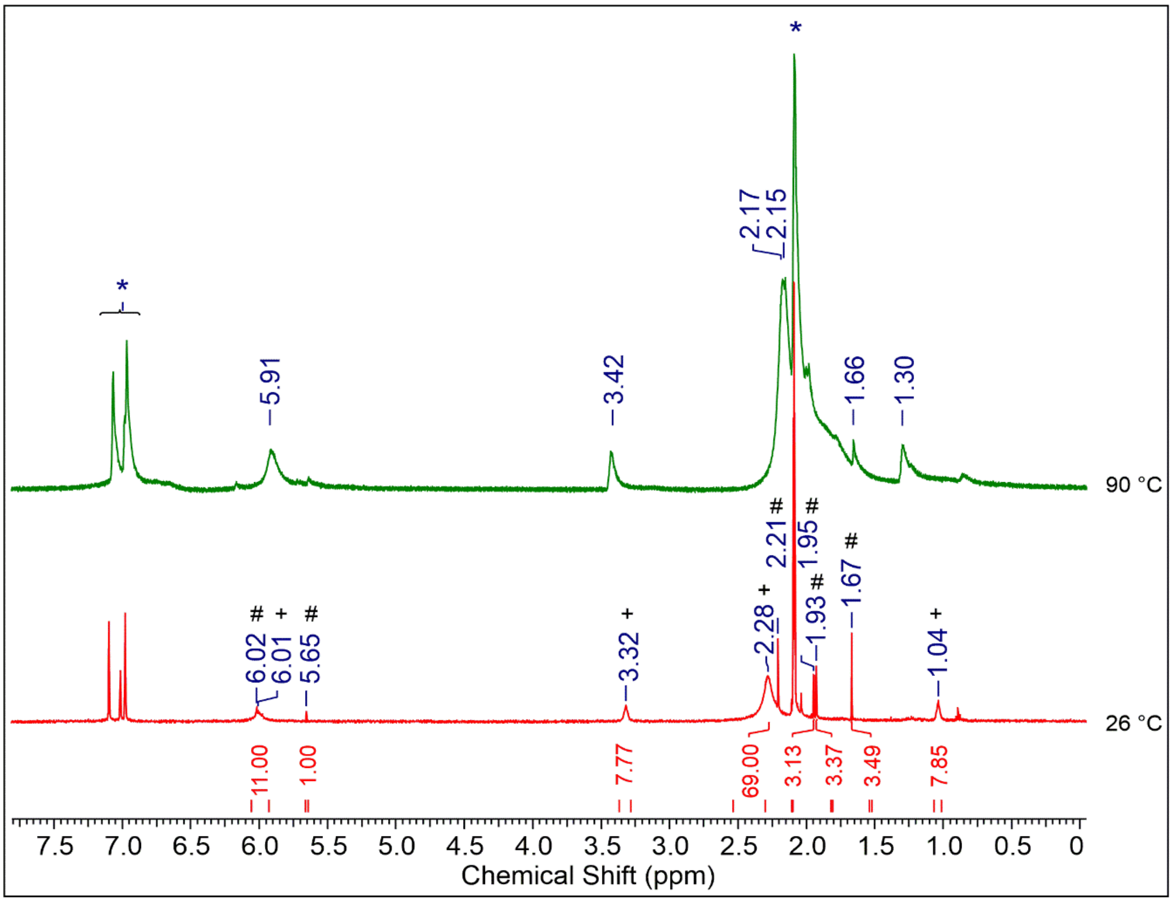

Figure S14. ^{1}H NMR spectra of [$Ce_3(Me_2pz)_9(Me_2pz{\cdot}CO_2)_3$(thf)] (**3**) in toluene-*d*$_8$ at 90 °C and after cooling to 26 °C. Signals for [$Ce(Me_2pz)_4$(thf)] are marked with + and unknown other product with #.

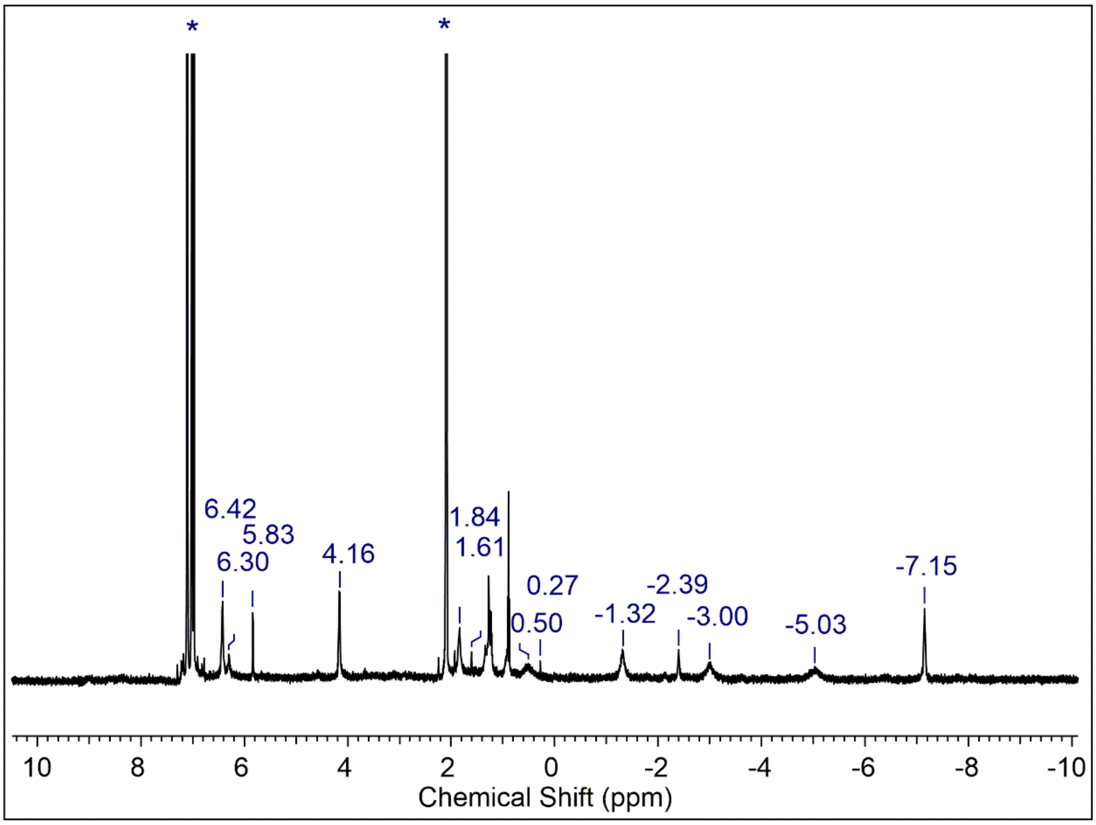

Figure S15. ^{1}H NMR spectrum (26 °C, 400.13 MHz, toluene-d_8) of $[Ce_4(Me_2pz{\cdot}CO_2)_{12}]\cdot10$ toluene (**5·toluene**).

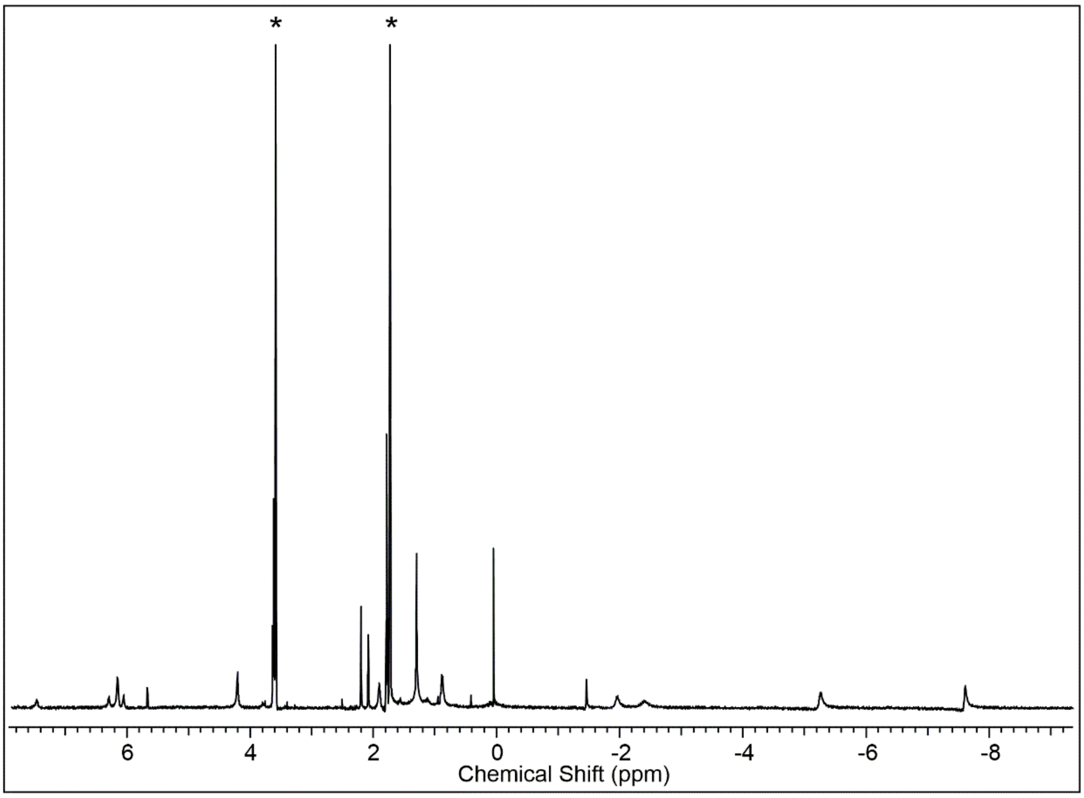

Figure S16. ^{1}H NMR spectrum (26 °C, 400.13 MHz, thf-d_8) of the reaction of $[Ce(Me_2pz)_3(thf)_2]$ with CO_2 in thf-d_8.

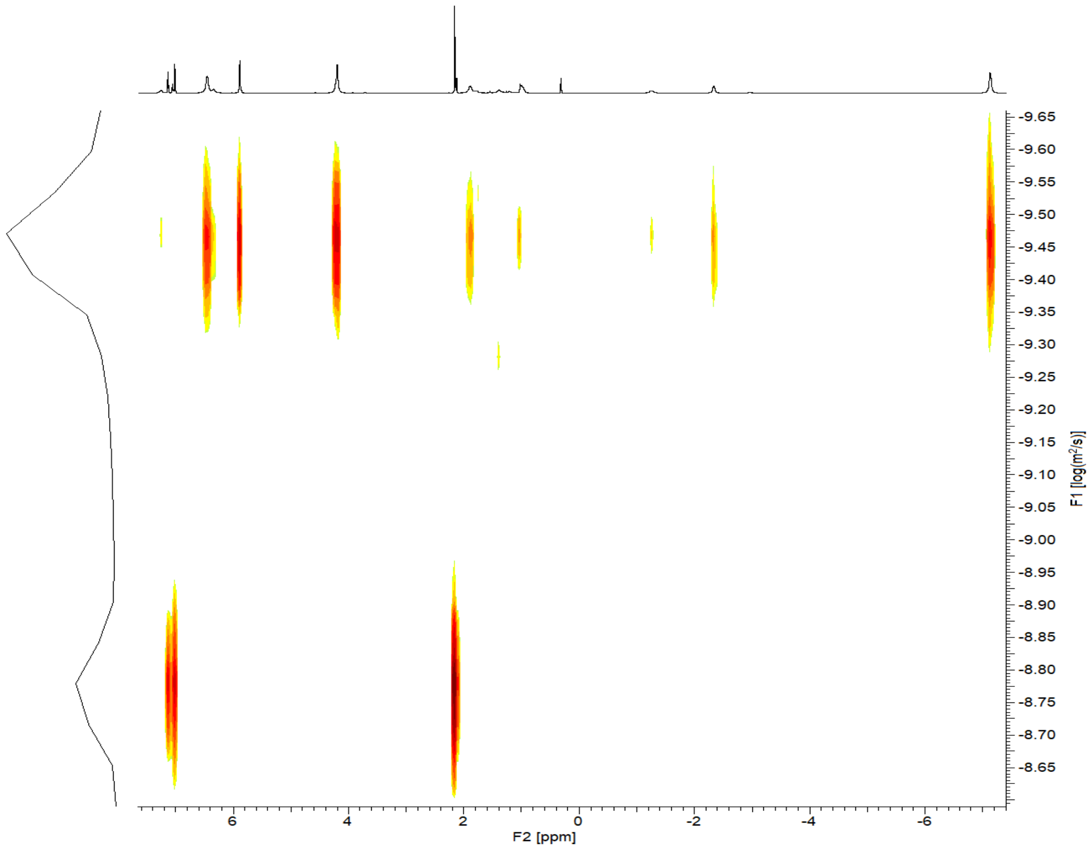

Figure S17. ^{1}H DOSY NMR spectrum (26 °C, 600.13 MHz, toluene-d_8) of $[Ce_4(Me_2pz{:}CO_2)_{12}]\cdot 10$ toluene (**5·toluene**) showing toluene at −8.78 and $[Ce_4(Me_2pz{:}CO_2)_{12}]$ at −9.47 log(m^2/s) (M = 1989 g·mol^{-1}).

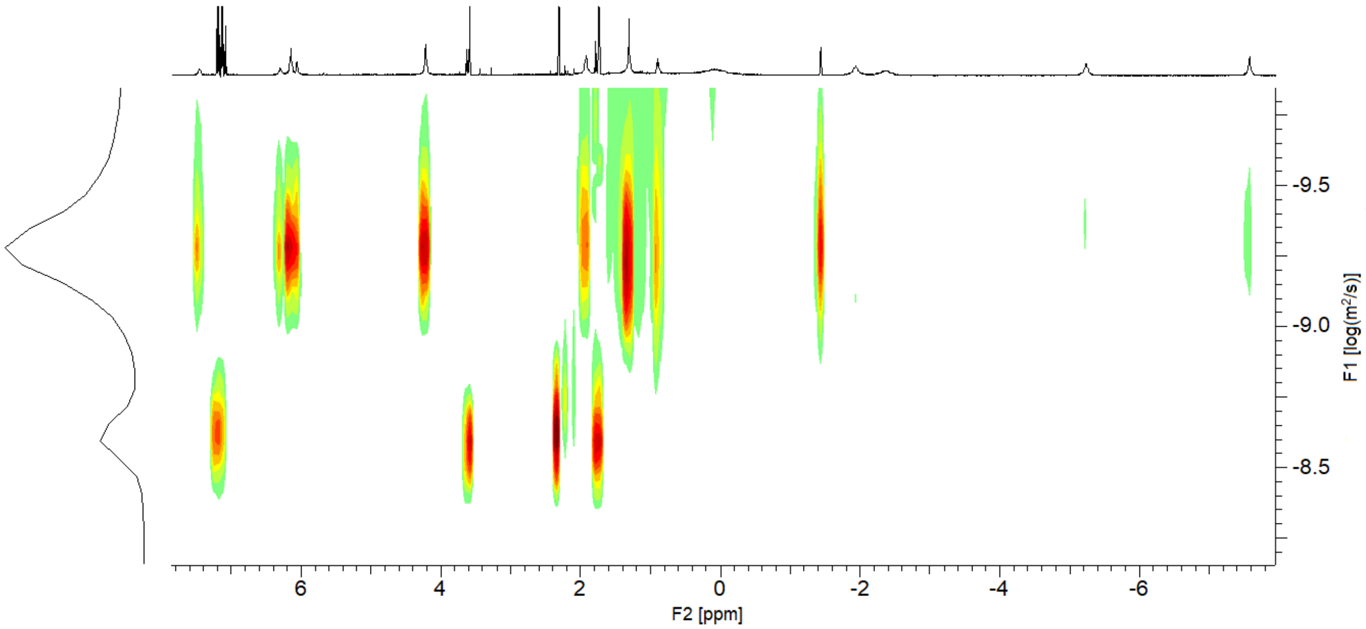

Figure S18. ^{1}H DOSY NMR spectrum (26 °C, 500.13 MHz, thf-d_8) of $[Ce_4(Me_2pz{:}CO_2)_{12}]\cdot 10$ toluene (**5·toluene**) showing thf-d_8 at −8.587, toluene at −8.620 (M = 75 g·mol^{-1}) and $[Ce_4(Me_2pz{:}CO_2)_{12}]$ at −9.282 log(m^2/s) (M = 1643 g·mol^{-1}).

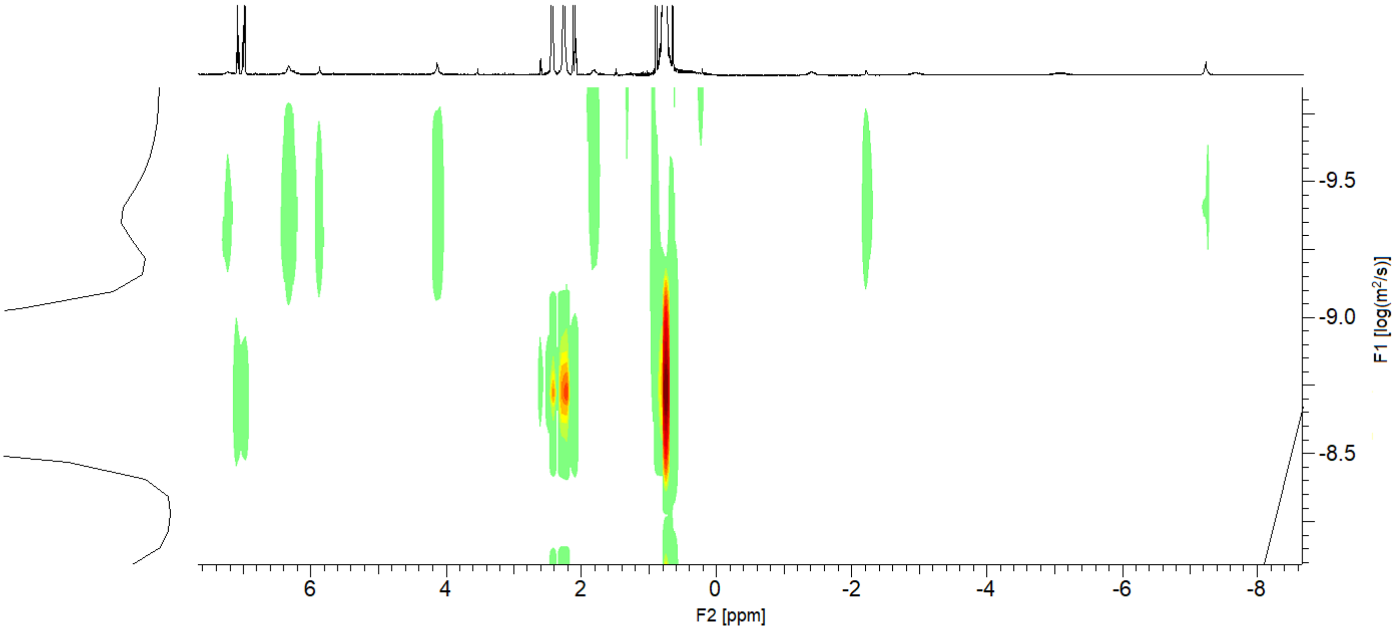

Figure S19. ^{1}H DOSY NMR spectrum (26 °C, 500.13 MHz, toluene-d_8) of $[Ce_4(Me_2pz{\cdot}CO_2)_{12}]\cdot 10$ toluene (**5·toluene**) + excess of 3,3-dimethyl-1,2-butene oxide showing toluene-d_8 at −8.658, 3,3-dimethyl-1,2-butene oxide at −8.722 (M = 114 g·mol^{-1}) and $[Ce_4(Me_2pz{\cdot}CO_2)_{12}]$ at −9.382 log(m^2/s) (M = 2357 g·mol^{-1}).

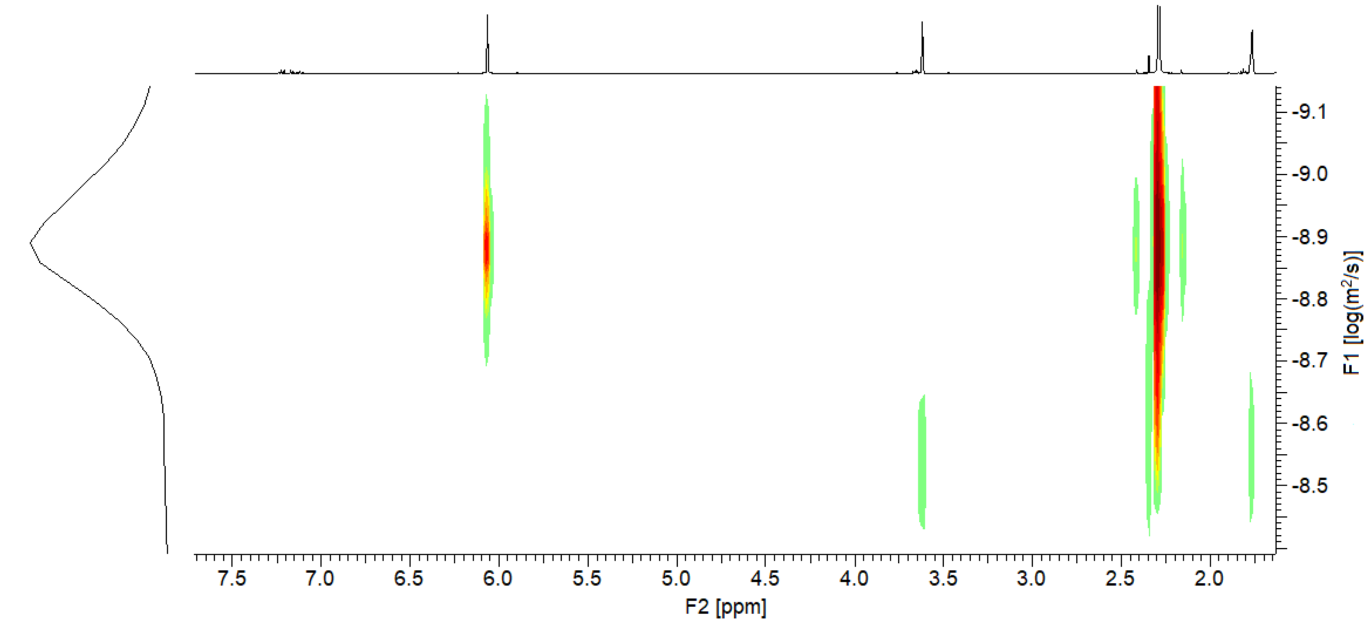

Figure S20. ^{1}H DOSY NMR spectrum (26 °C, 500.13 MHz, thf-d_8) of $[Ce(Me_2pz)_4(thf)]\cdot$(**1·thf**) showing thf-d_8 at −8.530 and $[Ce(Me_2pz)_4(thf)]$ at −8.896 log(m^2/s) (M = 354 g·mol^{-1}).

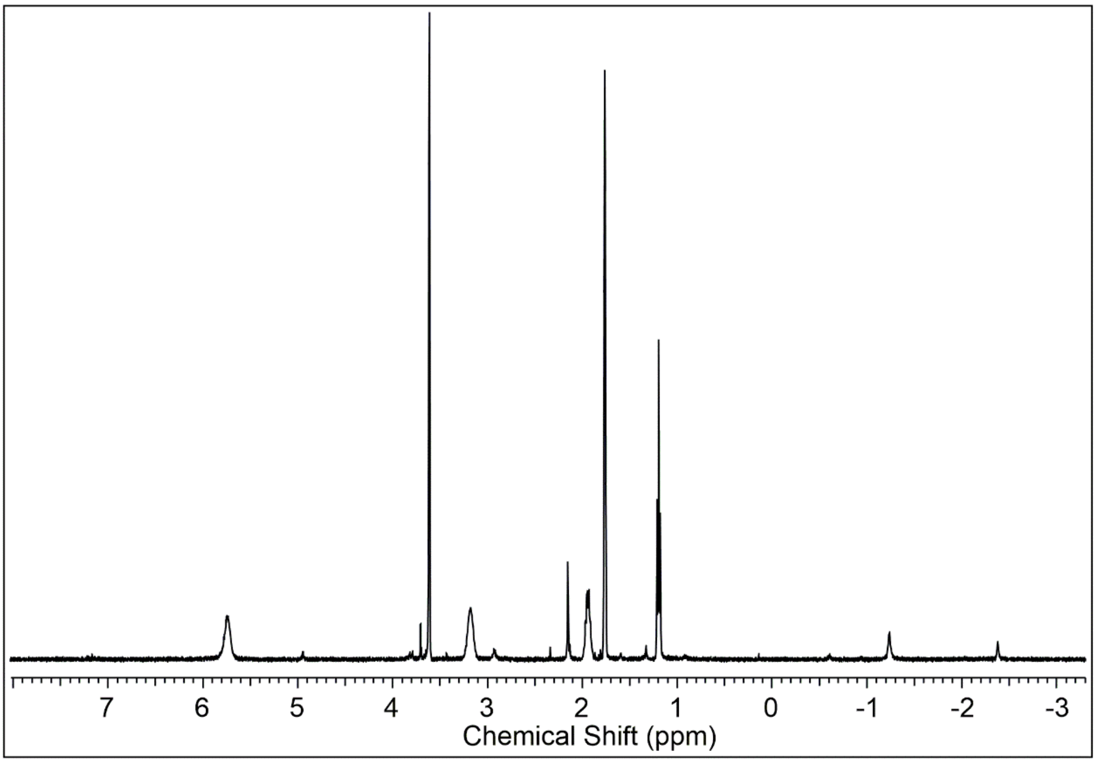

Figure S21. ^{1}H NMR spectrum (26 °C, 400.13 MHz, thf-d_8) of the reduction of [Ce(Me_2pz·CO_2)$_4$] (**2**) with TBAB.

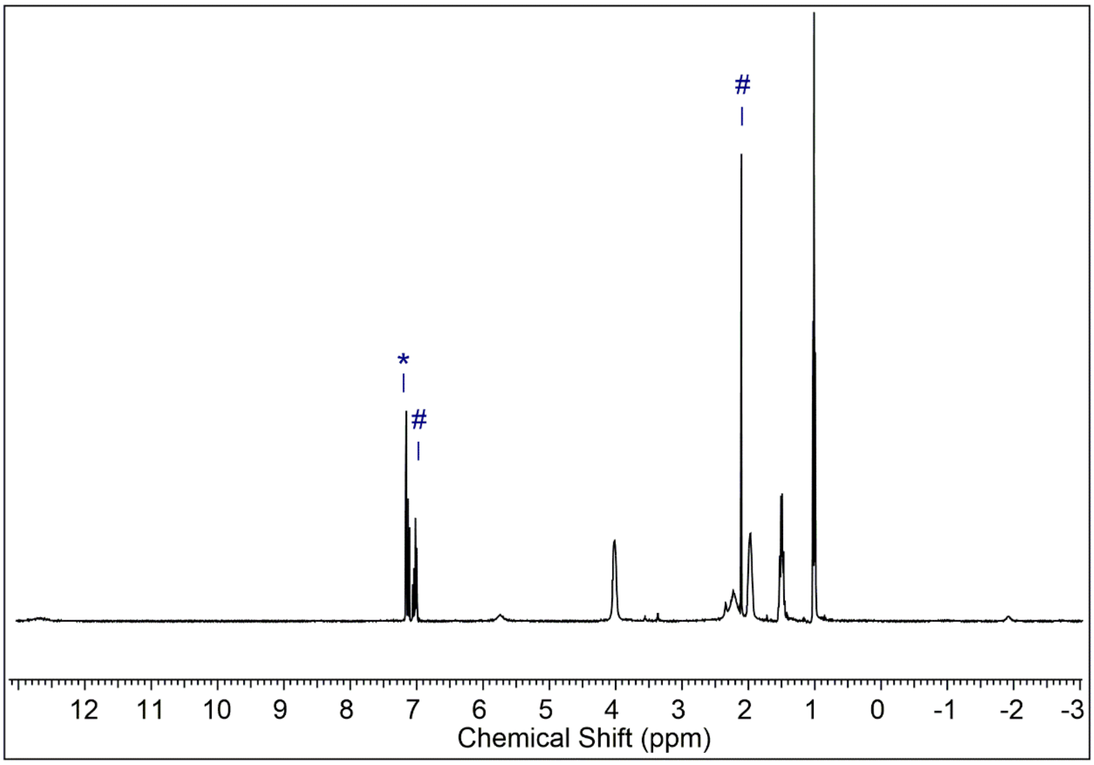

Figure S22. ^{1}H NMR spectrum (26 °C, 400.13 MHz, benzene-d_6) of the supernatant of the reduction of [Ce(Me_2pz·CO_2)$_4$] (**2**) together with TBAB. Toluene signals are marked with #.

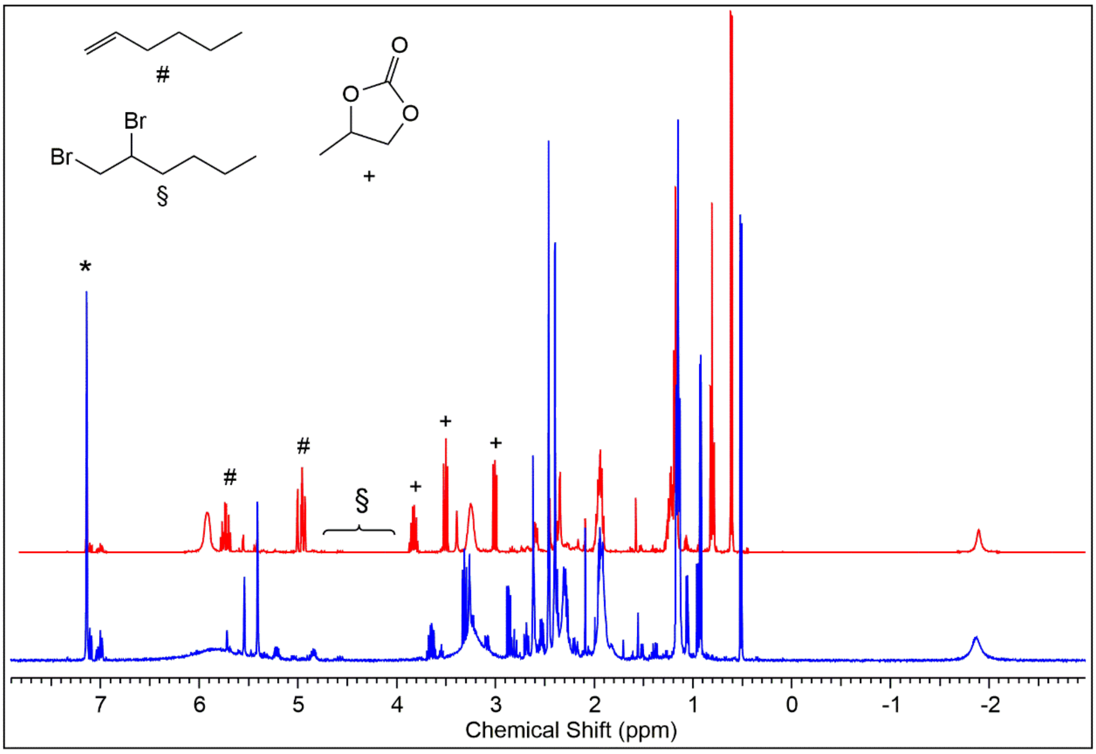

Figure S23. ^{1}H NMR spectrum (26 °C, 400.13 MHz, benzene-d_6) of the reaction of [Ce(Me$_2$pz·CO$_2$)$_4$] (**2**) with TBAB, CO_2 and propylene oxide (blue trace) and [Ce(Me$_2$pz·CO$_2$)$_4$] (**2**) with TBAB, CO_2, propylene oxide and 1-*n*-hexene (red trace). Signals for 1,2-dibromohexane would be expected in the region marked with §.

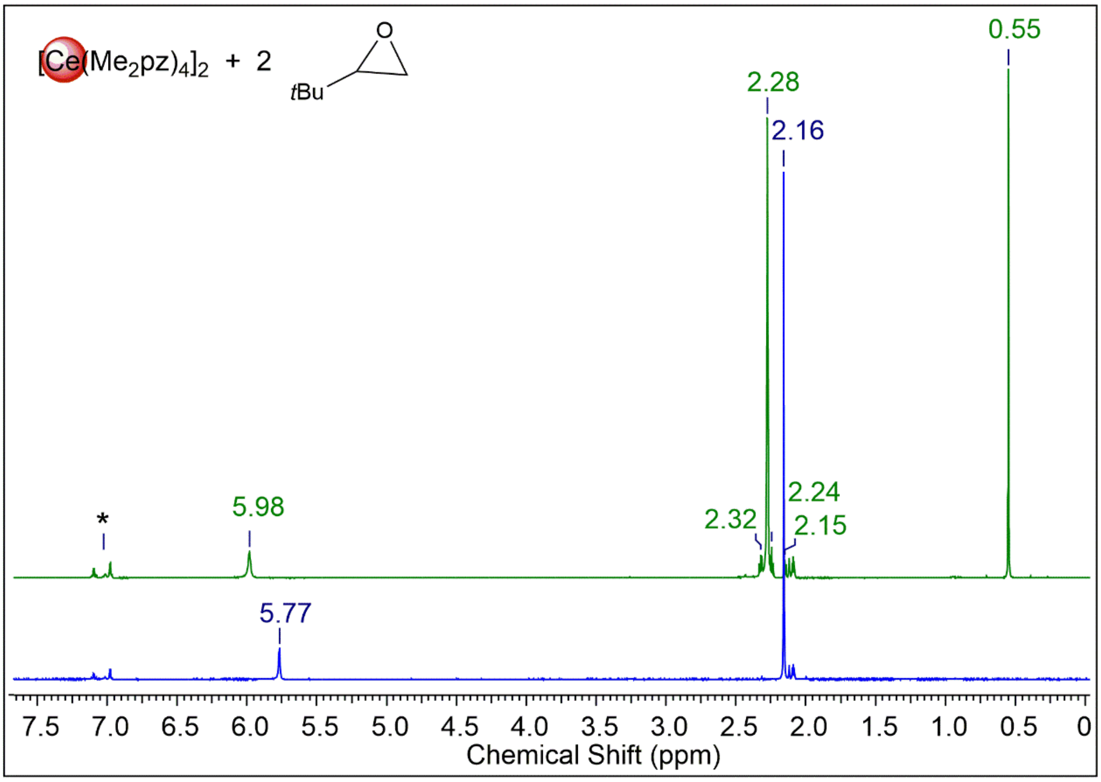

Figure S24. ^{1}H NMR spectrum (26 °C, 400.13 MHz, toluene-d_8) of [Ce(Me$_2$pz)$_4$]$_2$·(blue trace) and [Ce(Me$_2$pz)$_4$]$_2$ plus 3,3-dimethyl-1,2-butene oxide (green trace).

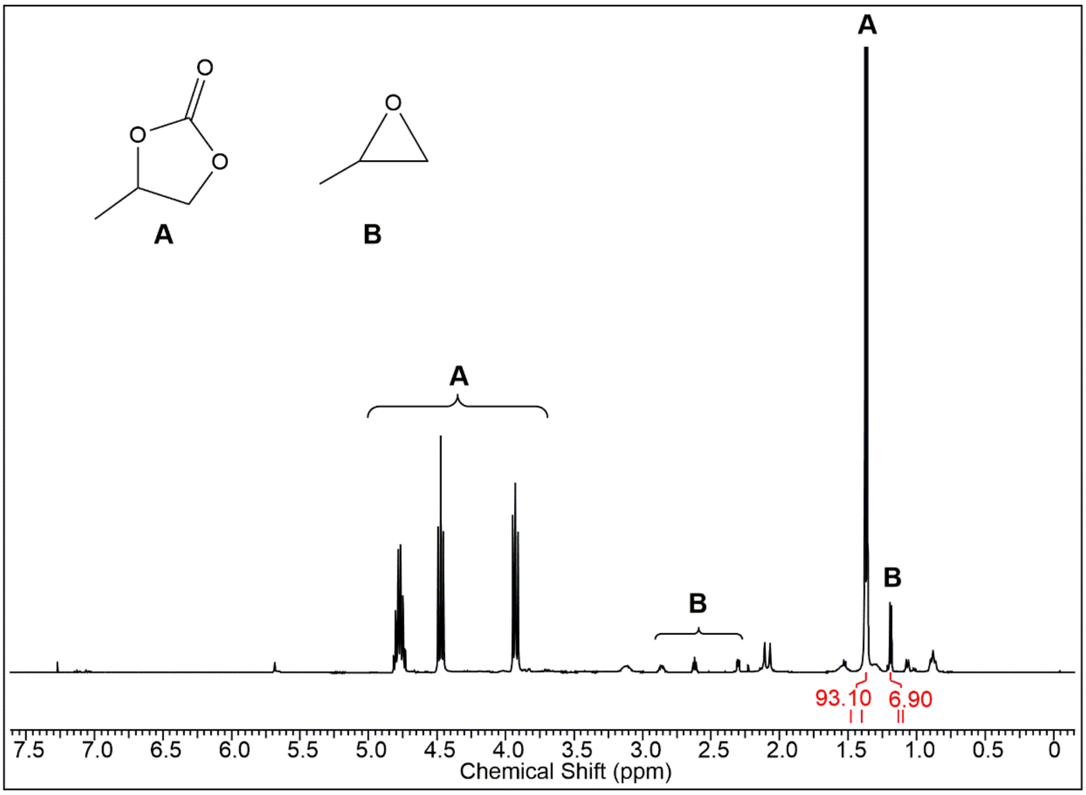

Figure S25. ^{1}H NMR (26 °C, 400.13 MHz, chloroform-*d*) of the product mixture of the catalytic formation of propylene carbonate using 0.5 mol% [Ce($Me_2pz)_4]_2$ (**1**) as a catalyst. The conversion was determined by the integral ratio of the methyl protons in propylene oxide and propylene carbonate.

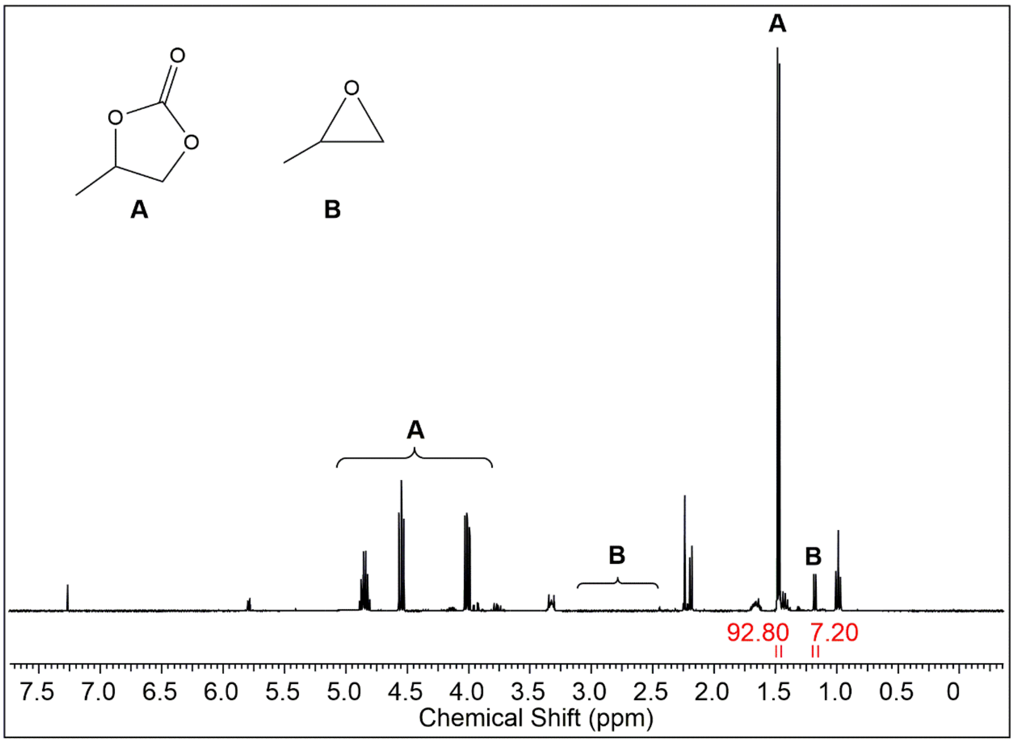

Figure S26. ^{1}H NMR (26 °C, 400.13 MHz, chloroform-*d*) of the product mixture of the catalytic formation of propylene carbonate using 1 mol% [Ce($Me_2pz \cdot CO_2)_4$]·2 thf (**2·thf**) as a catalyst. The conversion was determined by the integral ratio of the methyl protons in propylene oxide and propylene carbonate.

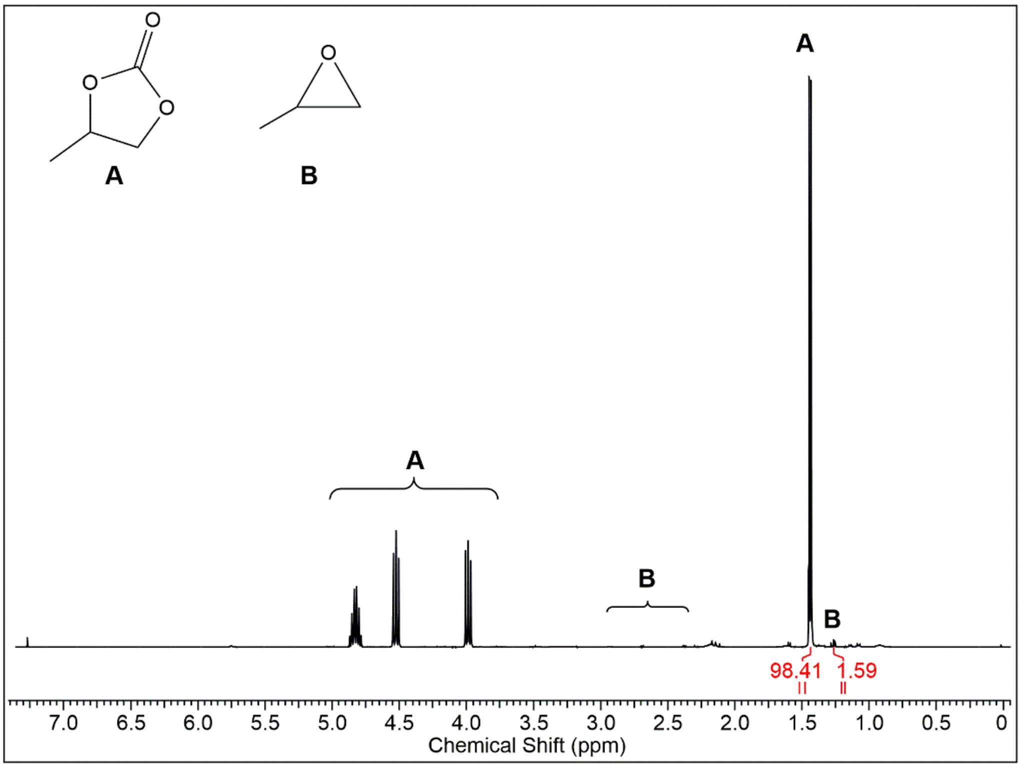

Figure S27. ^{1}H NMR (26 °C, 400.13 MHz, chloroform-*d*) of the product mixture of the catalytic formation of propylene carbonate using 0.25 mol% $[Ce(Me_2pz)_4]_2$ (**1**) as a catalyst at 90 °C and 10 bar CO_2 pressure for 1 hour. The conversion was determined by the integral ratio of the methyl protons in propylene oxide and propylene carbonate.

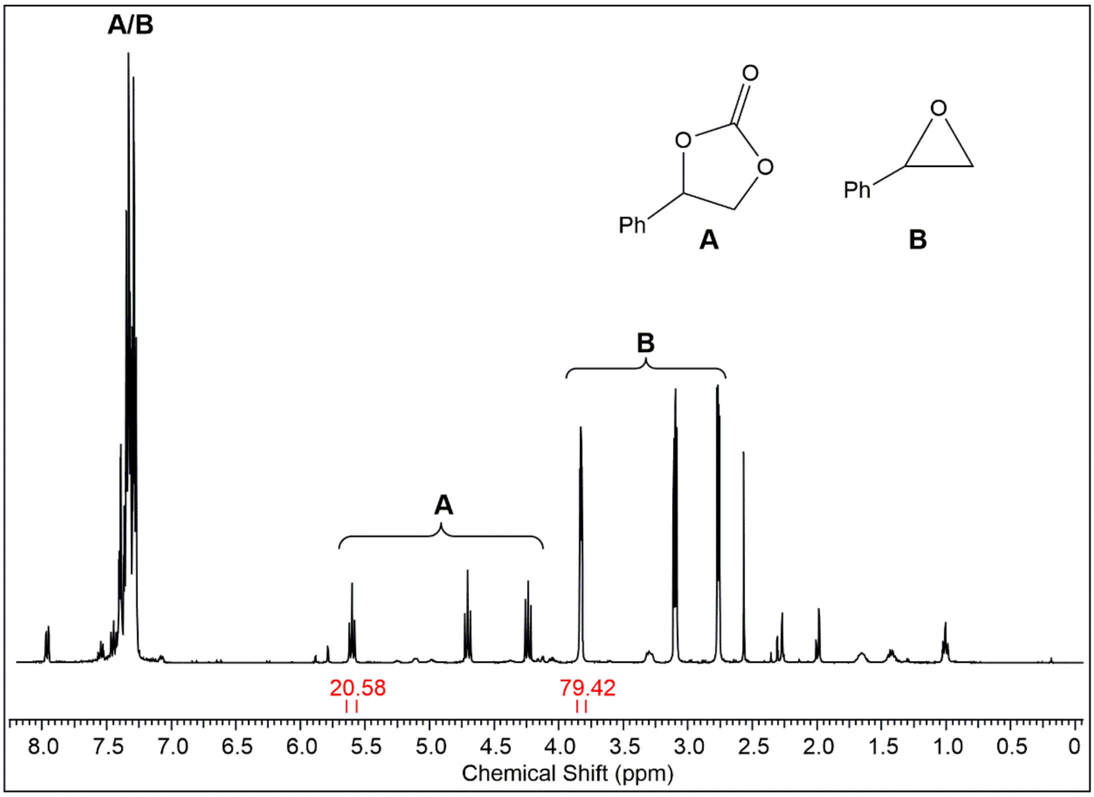

Figure S28. ^{1}H NMR (26 °C, 400.13 MHz, chloroform-*d*) of the product mixture of the catalytic formation of styrene carbonate using 0.5 mol% $[Ce(Me_2pz)_4]_2$ (**1**) as a catalyst. The conversion was determined by the integral ratio of the protons in α-position in styrene oxide and styrene carbonate.

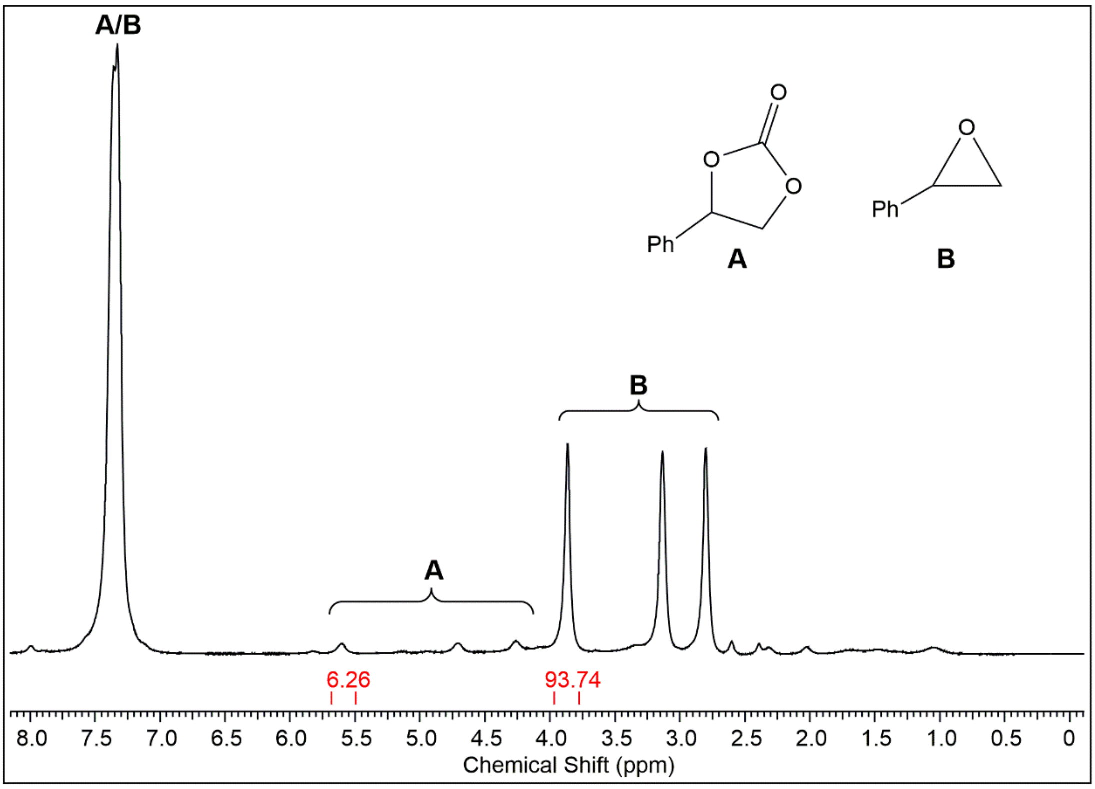

Figure S29. ^{1}H NMR (26 °C, 400.13 MHz, chloroform-*d*) of the product mixture of the catalytic formation of styrene carbonate using 0.25 mol% [Ce(Me_2pz)$_4$]$_2$ (**1**) as a catalyst. The conversion was determined by the integral ratio of the protons in α-position in styrene oxide and styrene carbonate.

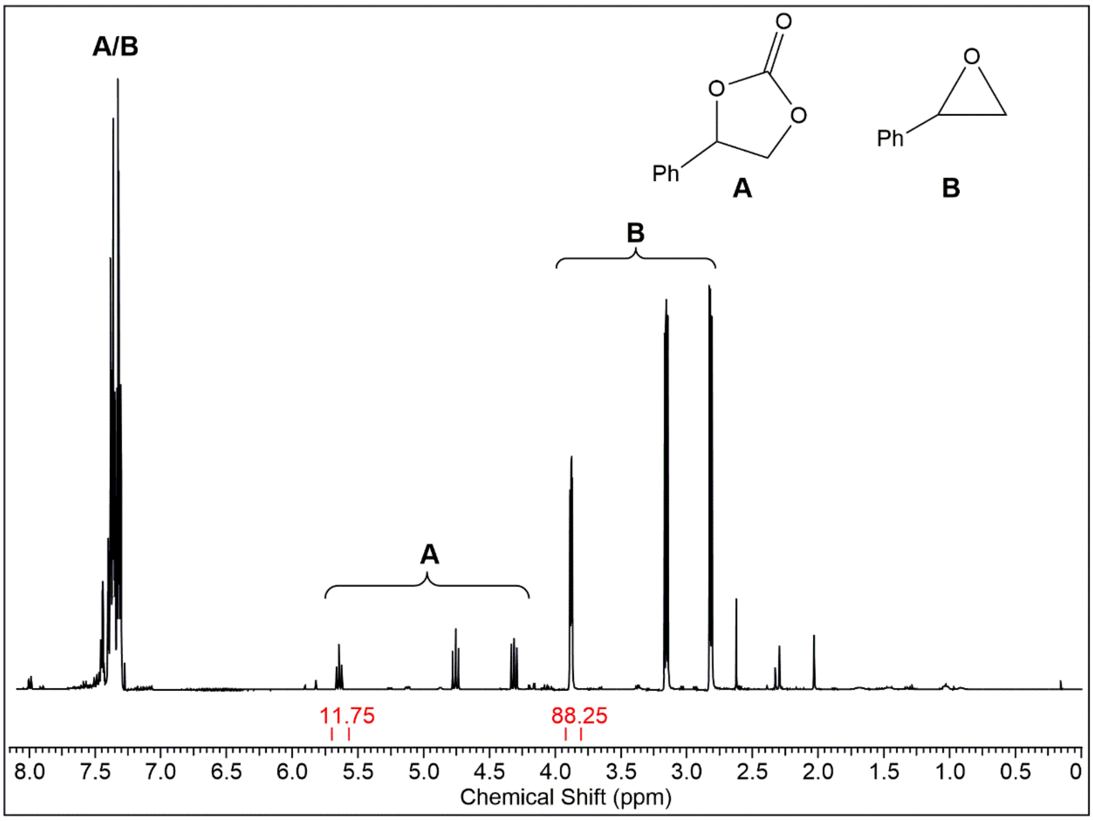

Figure S30. ^{1}H NMR (26 °C, 400.13 MHz, chloroform-*d*) of the product mixture of the catalytic formation of styrene carbonate using 0.25 mol% [Ce(Me_2pz)$_4$]$_2$ (**1**) as a catalyst at 90 °C and 10 bar CO_2 pressure. The conversion was determined by the integral ratio of the protons in α-position in styrene oxide and styrene carbonate.

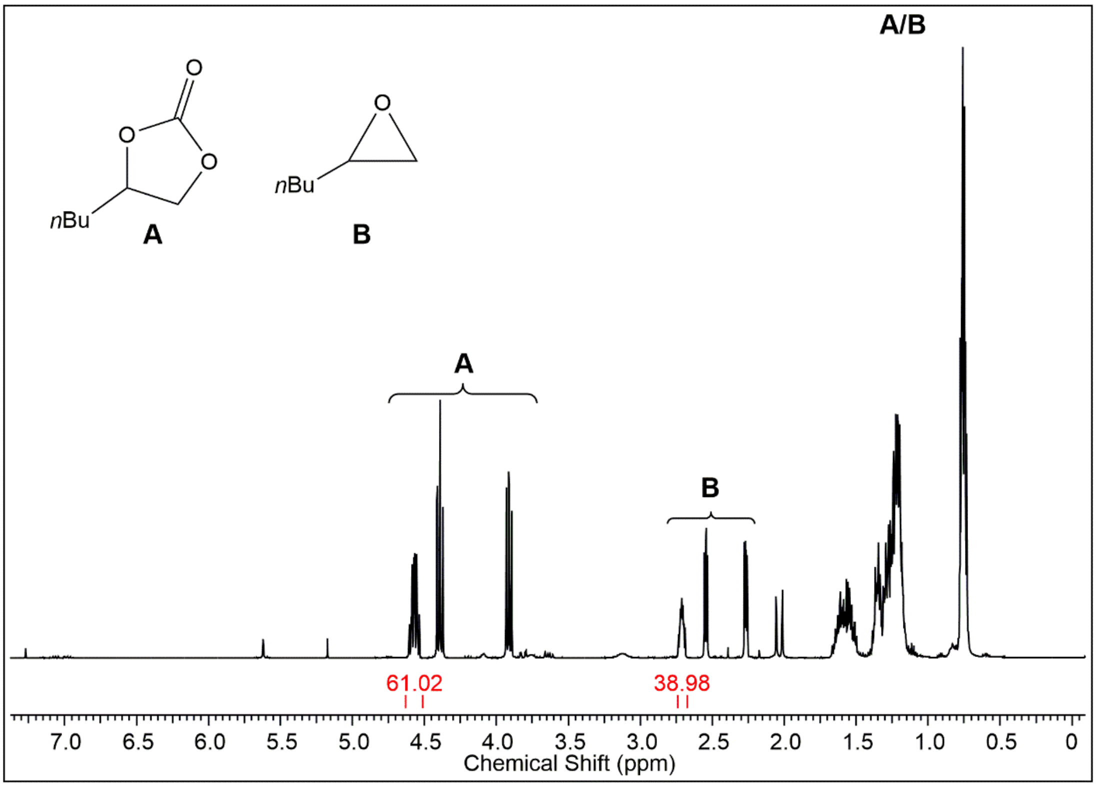

Figure S31. ^{1}H NMR (26 °C, 400.13 MHz, chloroform-*d*) of the product mixture of the catalytic formation of 1,2-*n*-hexylene carbonate using 0.5 mol% [Ce(Me_2pz)$_4$]$_2$ (**1**) as a catalyst. The conversion was determined by the integral ratio of the protons in α-position in 1,2-*n*-hexylene oxide and 1,2-*n*-hexylene carbonate.

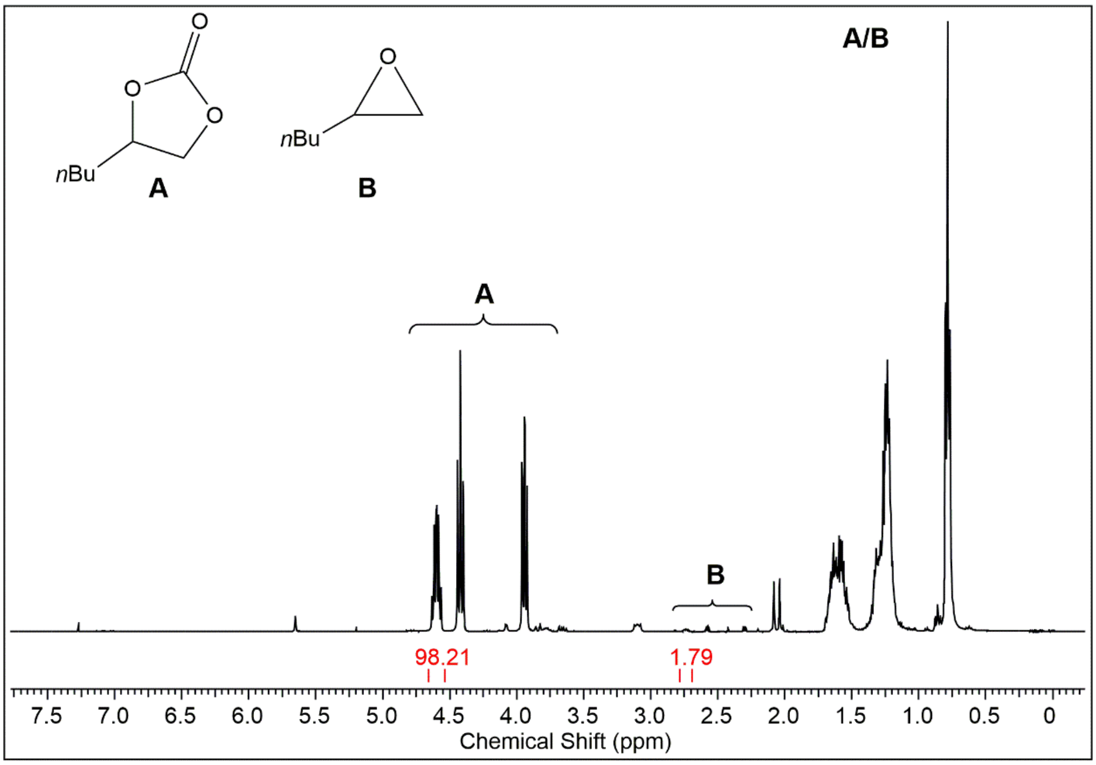

Figure S32. ^{1}H NMR (26 °C, 400.13 MHz, chloroform-*d*) of the product mixture of the catalytic formation of 1,2-*n*-hexylene carbonate using 0.5 mol% [Ce(Me_2pz)$_4$]$_2$ (**1**) as a catalyst at 90 °C. The conversion was determined by the integral ratio of the protons in α-position in 1,2-*n*-hexylene oxide and 1,2-*n*-hexylene carbonate.

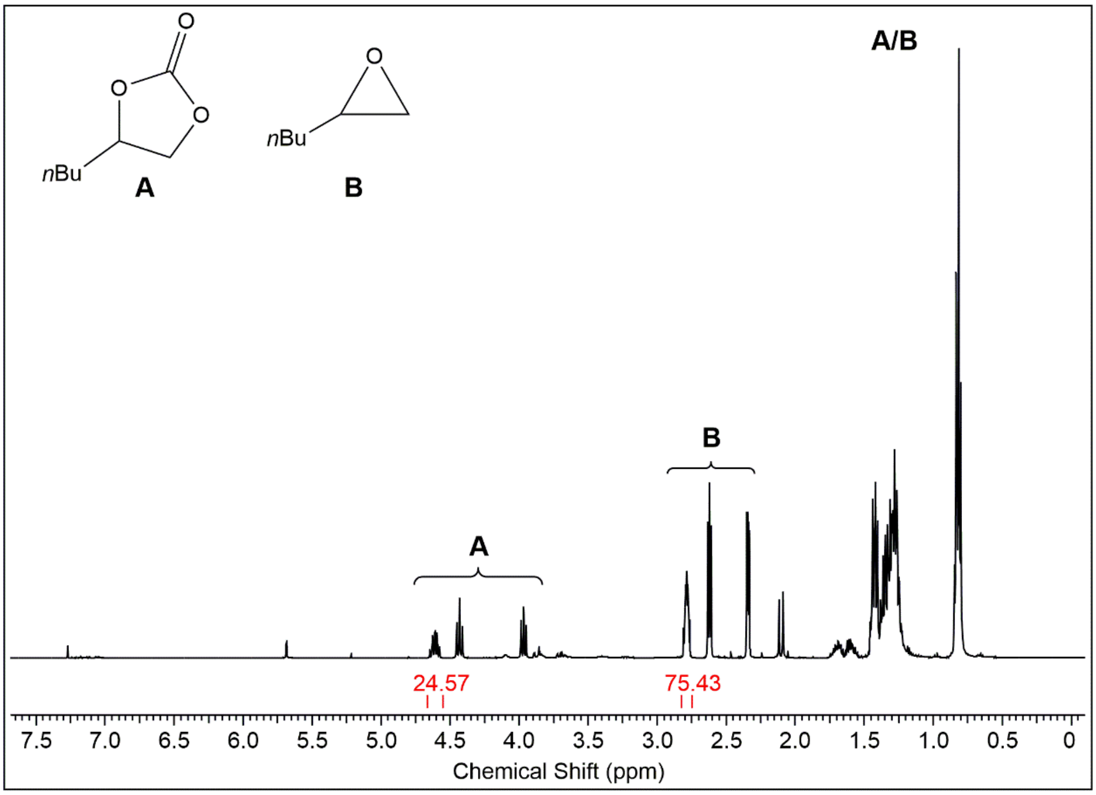

Figure S33. ^{1}H NMR (26 °C, 400.13 MHz, chloroform-*d*) of the product mixture of the catalytic formation of 1,2-*n*-hexylene carbonate using 0.5 mol% $[Ce(Me_2pz)_4]_2$ (**1**) as a catalyst without TBAB as cocatalyst at 90 °C. The conversion was determined by the integral ratio of the protons in α-position in 1,2-*n*-hexylene oxide and 1,2-*n*-hexylene carbonate.

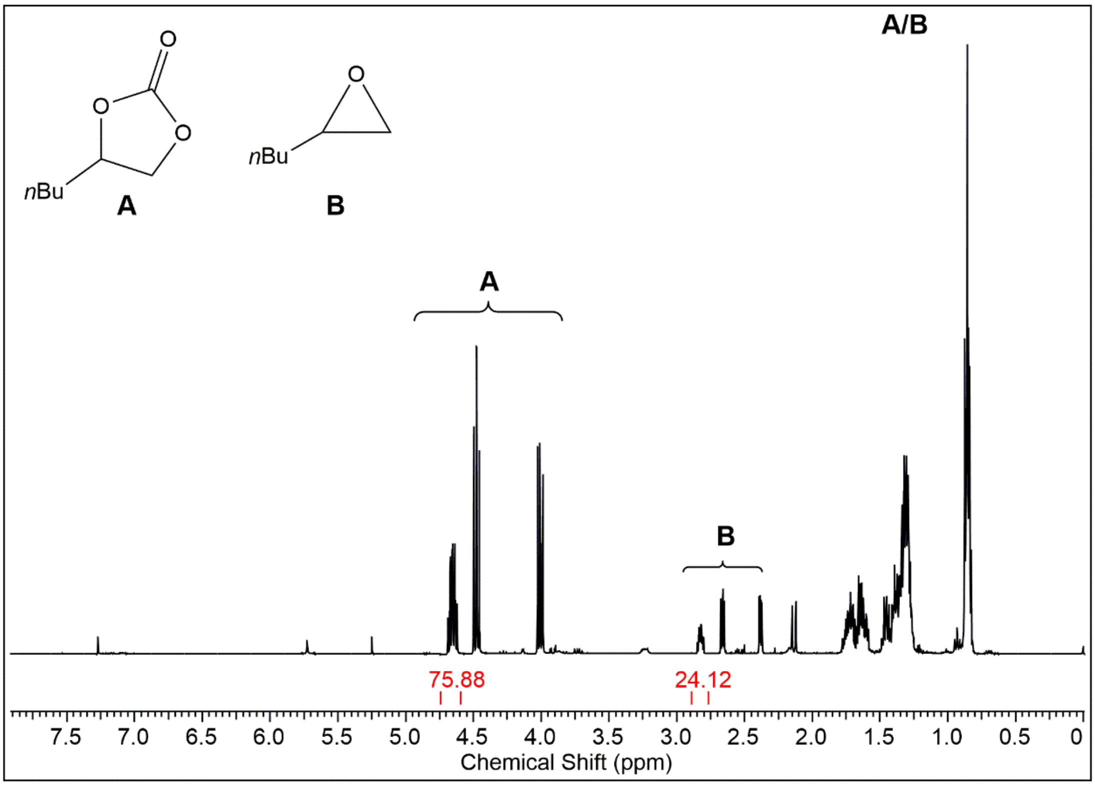

Figure S34. ^{1}H NMR (26 °C, 400.13 MHz, chloroform-*d*) of the product mixture of the catalytic formation of 1,2-*n*-hexylene carbonate using 0.5 mol% $[Ce(Me_2pz)_4]_2$ (**1**) as a catalyst at 10 bar CO_2 pressure. The conversion was determined by the integral ratio of the protons in α-position in 1,2-*n*-hexylene oxide and 1,2-*n*-hexylene carbonate.

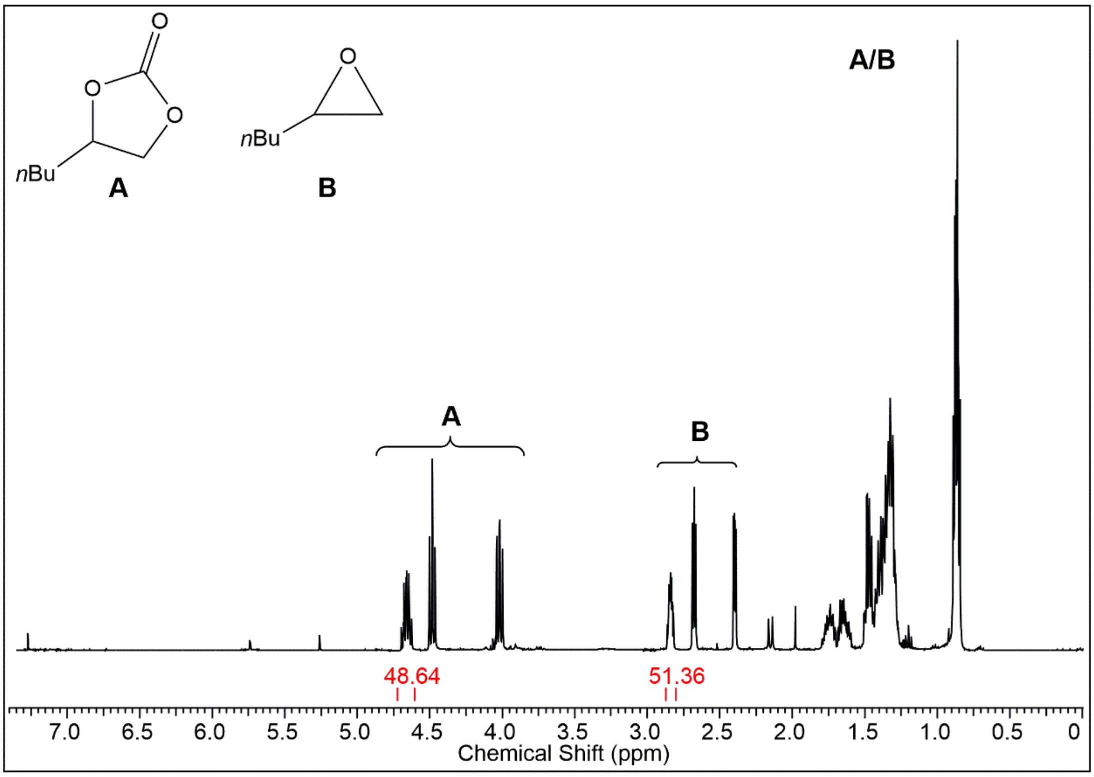

Figure S35. ^{1}H NMR (26 °C, 400.13 MHz, chloroform-*d*) of the product mixture of the catalytic formation of 1,2-*n*-hexylene carbonate using 0.25 mol% [Ce(Me$_2$pz)$_4$]$_2$ (**1**) as a catalyst at 90°C and 10 bar CO_2 pressure for 1 hour. The conversion was determined by the integral ratio of the protons in α-position in 1,2-*n*-hexylene oxide and 1,2-*n*-hexylene carbonate.

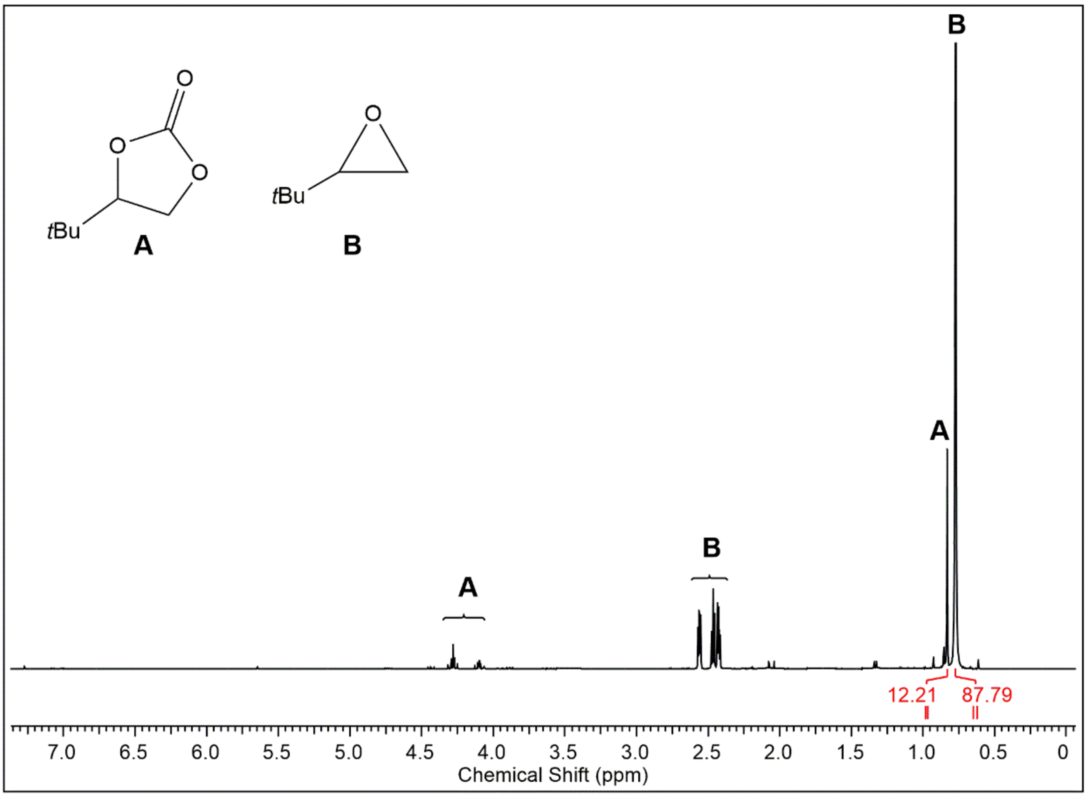

Figure S36. ^{1}H NMR (26 °C, 400.13 MHz, chloroform-*d*) of the product mixture of the catalytic formation of 3,3-dimethyl-1,2-butene carbonate using 0.5 mol% [Ce(Me$_2$pz)$_4$]$_2$ (**1**) as a catalyst. The conversion was determined by the integral ratio of the *tert*-butyl protons in 3,3-dimethyl-1,2-butene oxide and 3,3-dimethyl-1,2-butylene carbonate.

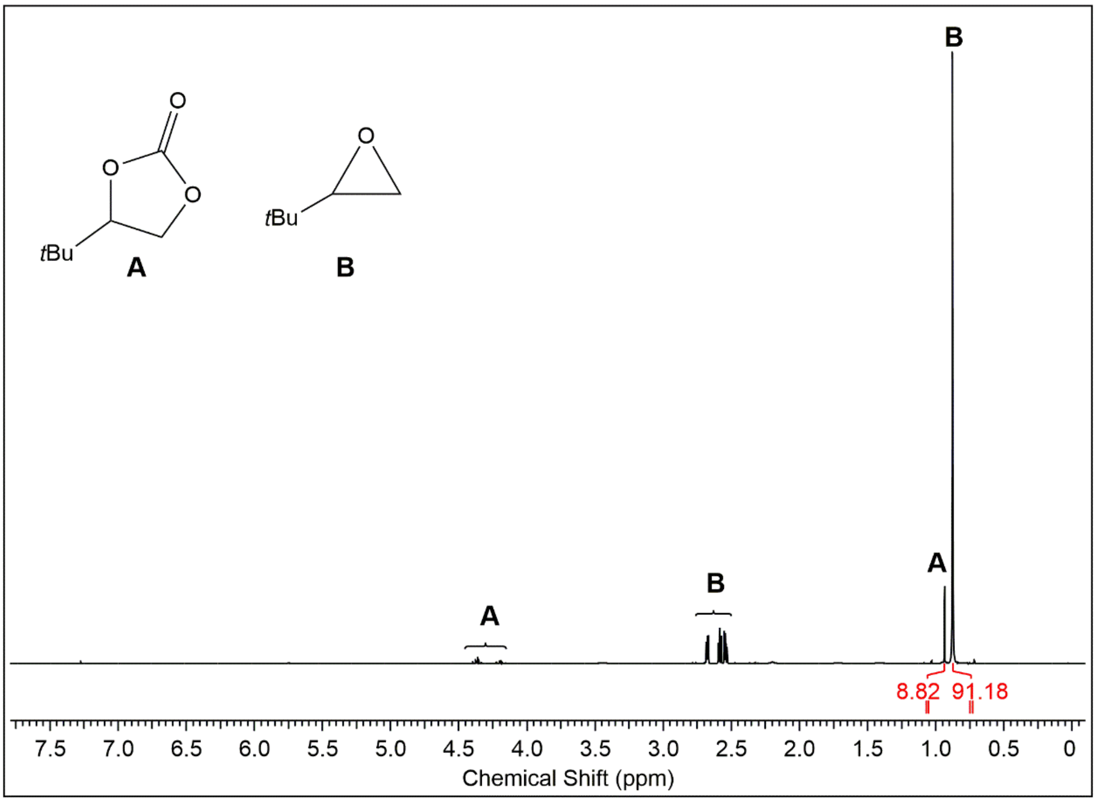

Figure S37. ^{1}H NMR (26 °C, 400.13 MHz, chloroform-*d*) of the product mixture of the catalytic formation of 3,3-dimethyl-1,2-butene carbonate using 0.5 mol% [Ce(Me_2pz)$_4$]$_2$ (**1**) as a catalyst at 10 bar CO_2 pressure. The conversion was determined by the integral ratio of the *tert*-butyl protons in 3,3-dimethyl-1,2-butene oxide and 3,3-dimethyl-1,2-butylene carbonate.

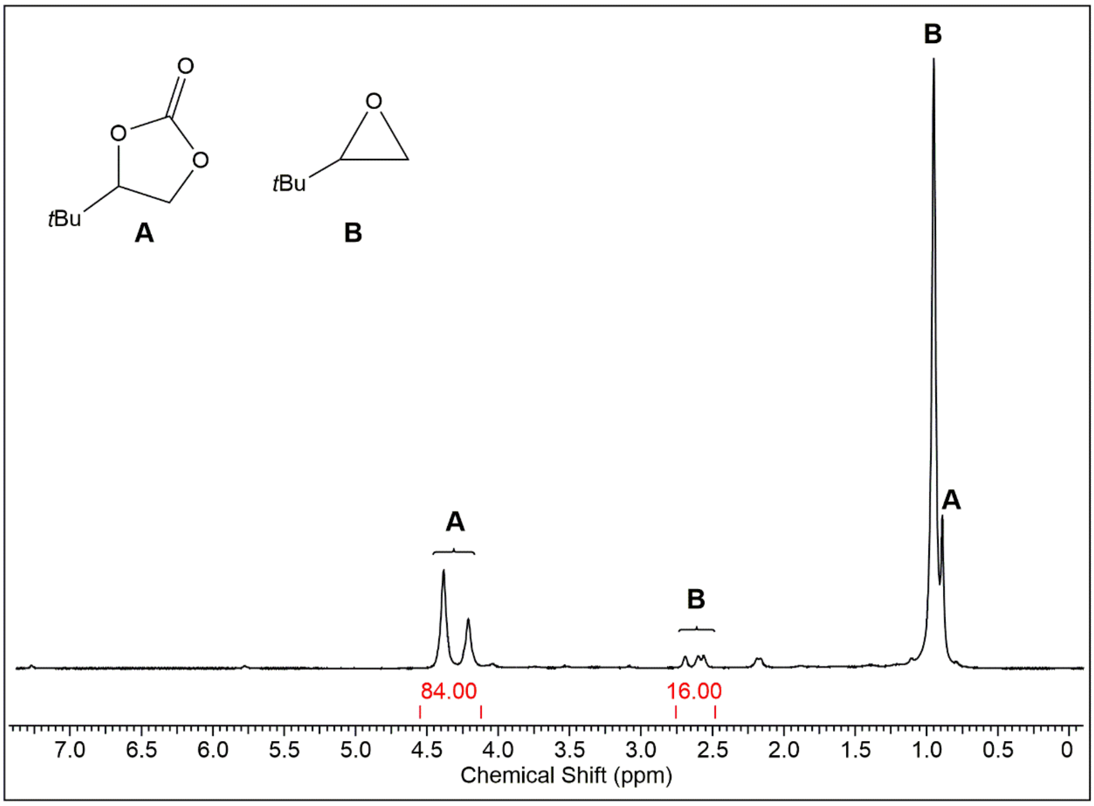

Figure S38. ^{1}H NMR (26 °C, 400.13 MHz, chloroform-*d*) of the product mixture of the catalytic formation of 3,3-dimethyl-1,2-butene carbonate using 0.25 mol% [Ce(Me_2pz)$_4$]$_2$ (**1**) as a catalyst at 90 °C and 10 bar CO_2 pressure. The conversion was determined by the integral ratio of the protons in α- and β-position in 3,3-dimethyl-1,2-butene oxide and 3,3-dimethyl-1,2-butylene carbonate.

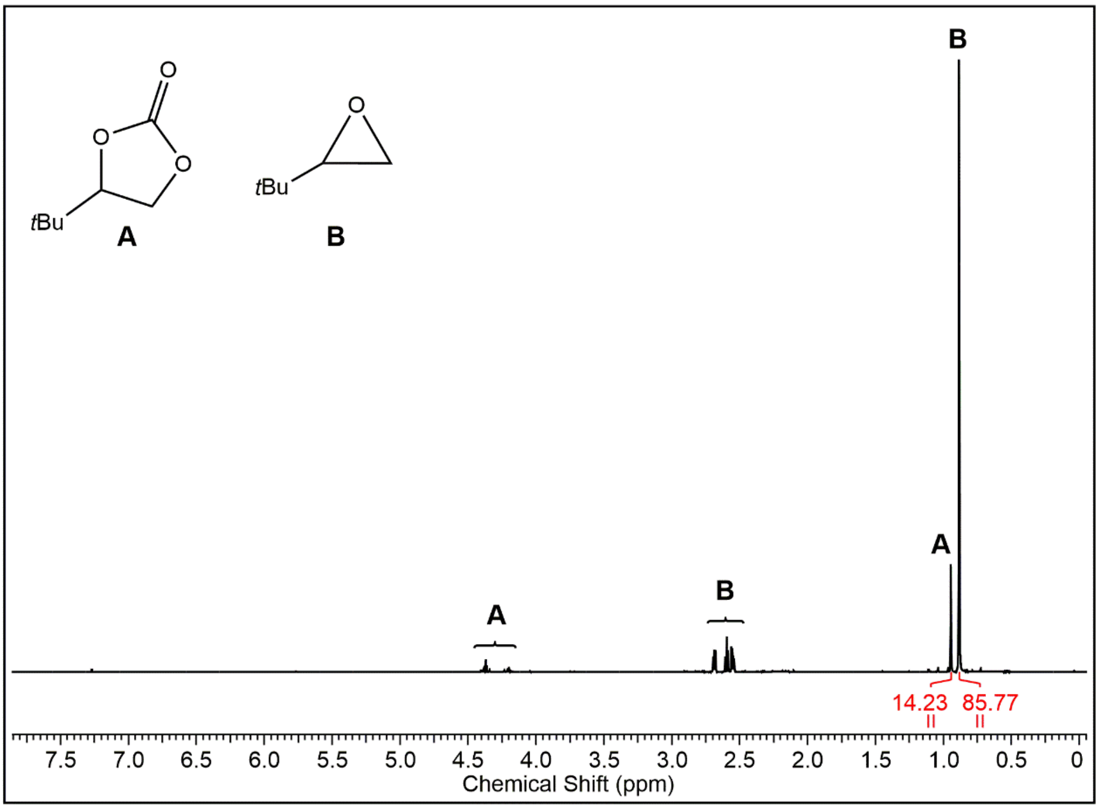

Figure S39. ^{1}H NMR (26 °C, 400.13 MHz, chloroform-*d*) of the product mixture of the catalytic formation of 3,3-dimethyl-1,2-butene carbonate using 0.25 mol% [Ce(Me$_2$pz)$_4$]$_2$ (**1**) as a catalyst at 90 °C and 10 bar CO_2 pressure after 1 hour. The conversion was determined by the integral ratio of the *tert*-butyl protons in 3,3-dimethyl-1,2-butene oxide and 3,3-dimethyl-1,2-butylene carbonate.

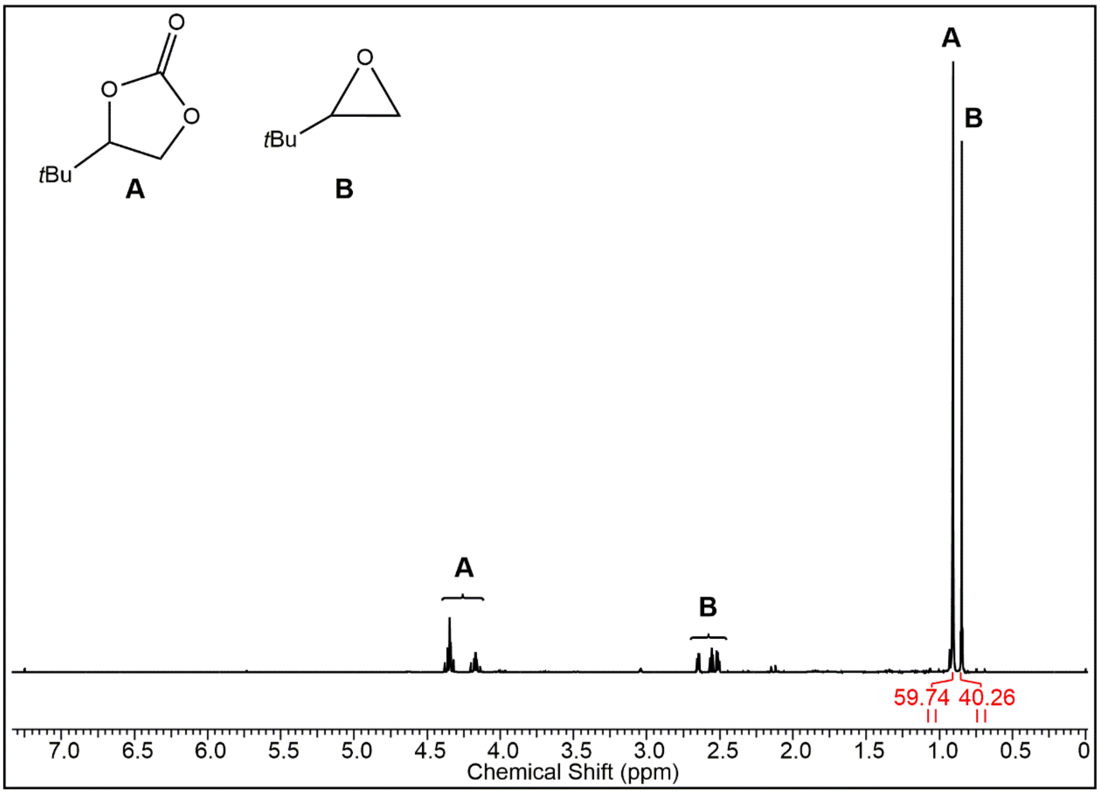

Figure S40. ^{1}H NMR (26 °C, 400.13 MHz, chloroform-*d*) of the product mixture of the catalytic formation of 3,3-dimethyl-1,2-butene carbonate using 0.1 mol% [Ce(Me$_2$pz)$_4$]$_2$ (**1**) as a catalyst at 90 °C and 10 bar CO_2 pressure after 1 hour. The conversion was determined by the integral ratio of the *tert*-butyl protons in 3,3-dimethyl-1,2-butene oxide and 3,3-dimethyl-1,2-butylene carbonate.

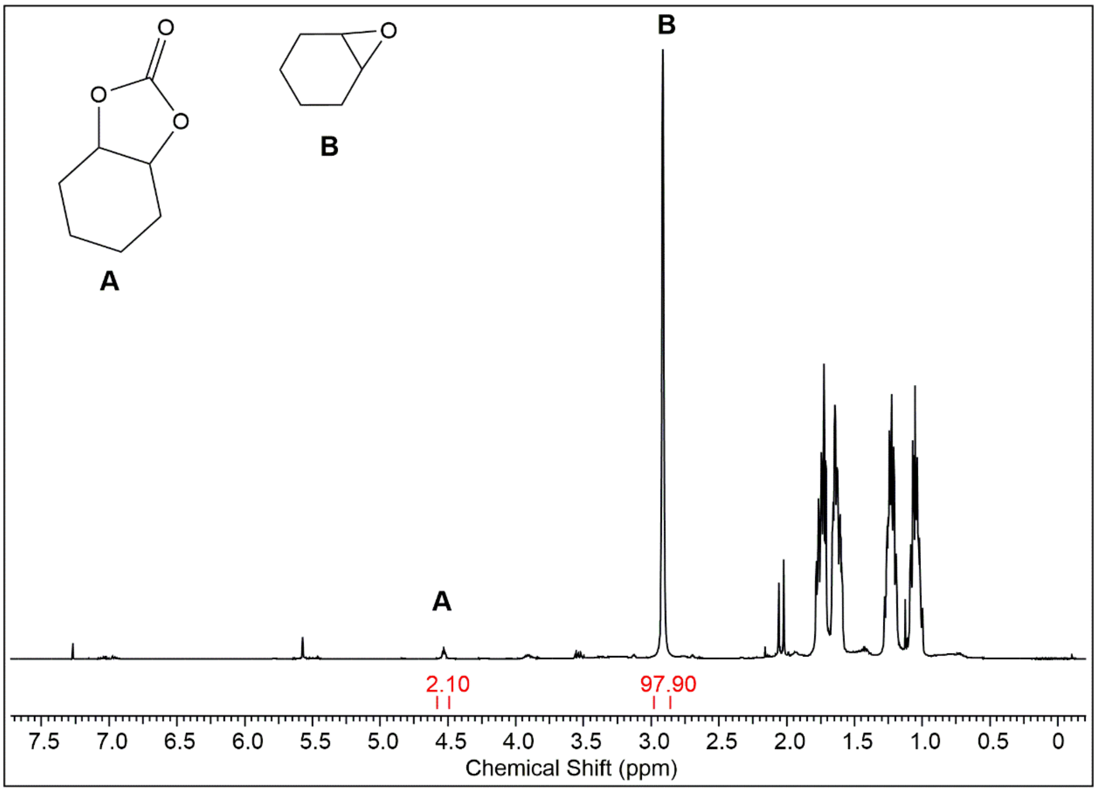

Figure S41. ^{1}H NMR (26 °C, 400.13 MHz, chloroform-*d*) of the product mixture of the catalytic formation of cyclohexene carbonate using 0.5 mol% [Ce(Me$_2$pz)$_4$]$_2$ (**1**) as a catalyst. The conversion was determined by the integral ratio of the protons in α-position in cyclohexene oxide and cyclohexene carbonate.

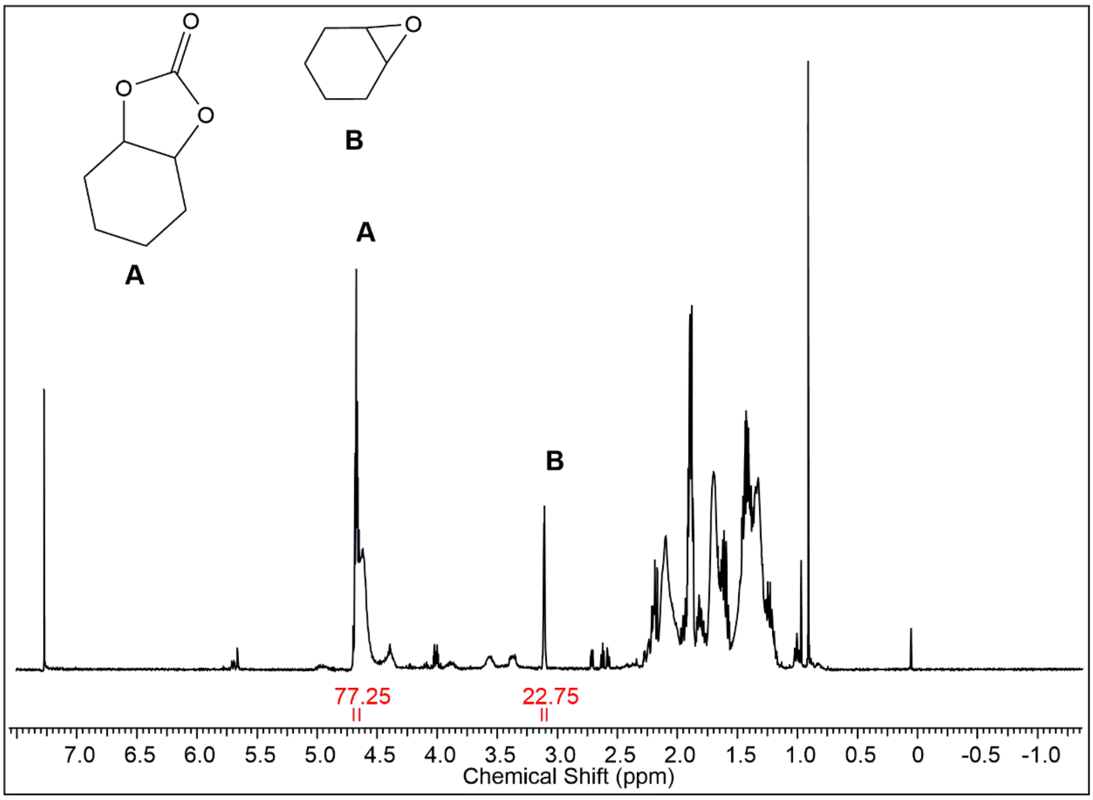

Figure S42. ^{1}H NMR (26 °C, 400.13 MHz, chloroform-*d*) of the product mixture of the catalytic formation of cyclohexene carbonate using 0.25 mol%[Ce(Me$_2$pz)$_4$]$_2$ (**1**) as a catalyst at 90 °C and 10 bar CO_2 pressure. The conversion was determined by the integral ratio of the protons in α-position in cyclohexene oxide and cyclohexene carbonate.

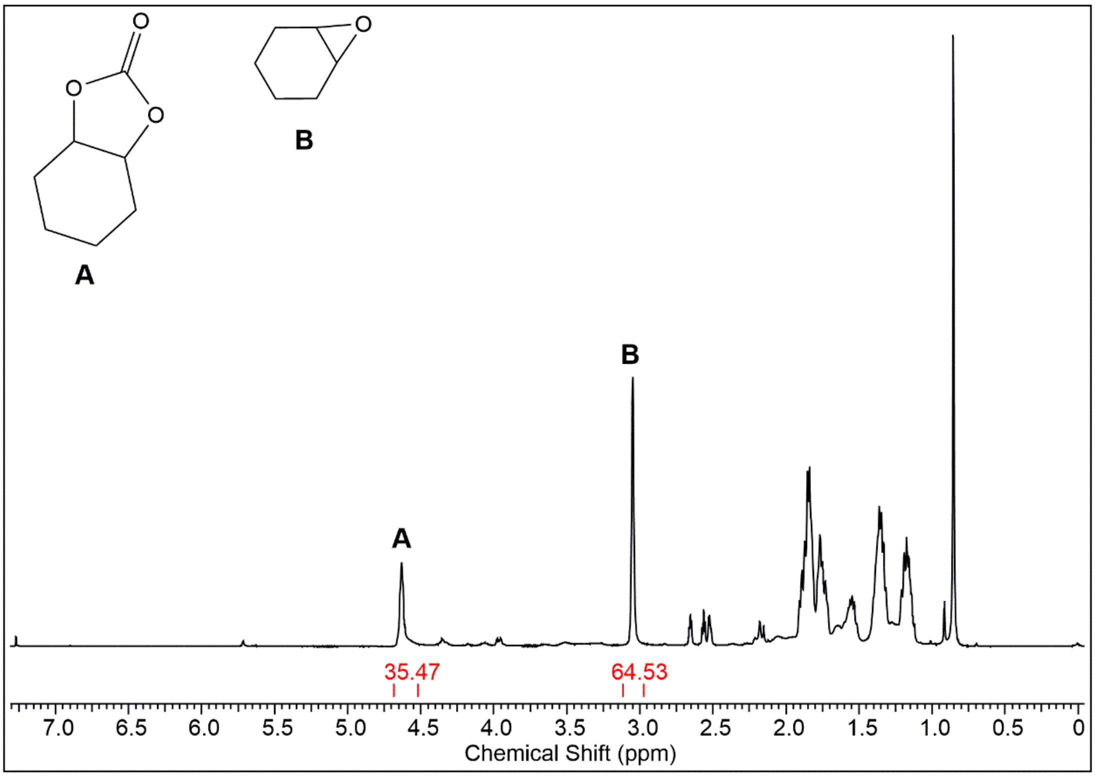

Figure S43. ^{1}H NMR (26 °C, 400.13 MHz, chloroform-*d*) of the product mixture of the catalytic formation of cyclohexene carbonate using 0.1 mol%[Ce(Me$_2$pz)$_4$]$_2$ (**1**) as a catalyst at 90 °C and 10 bar CO_2 pressure. The conversion was determined by the integral ratio of the protons in α-position in cyclohexene oxide and cyclohexene carbonate.

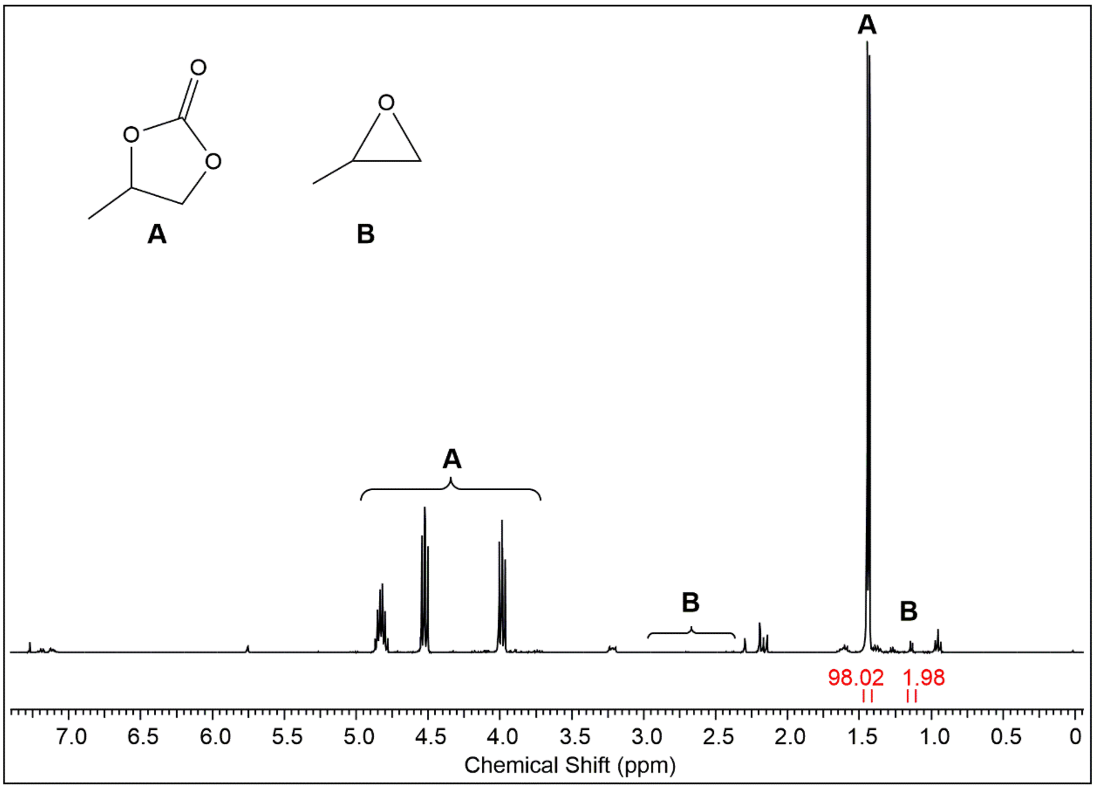

Figure S44. ^{1}H NMR (26 °C, 400.13 MHz, chloroform-*d*) of the product mixture of the catalytic formation of propylene carbonate using 0.25 mol% [Ce$_4$(Me$_2$pz)$_{12}$] (**4**) as a catalyst. The conversion was determined by the integral ratio of the methyl protons in propylene oxide and propylene carbonate.

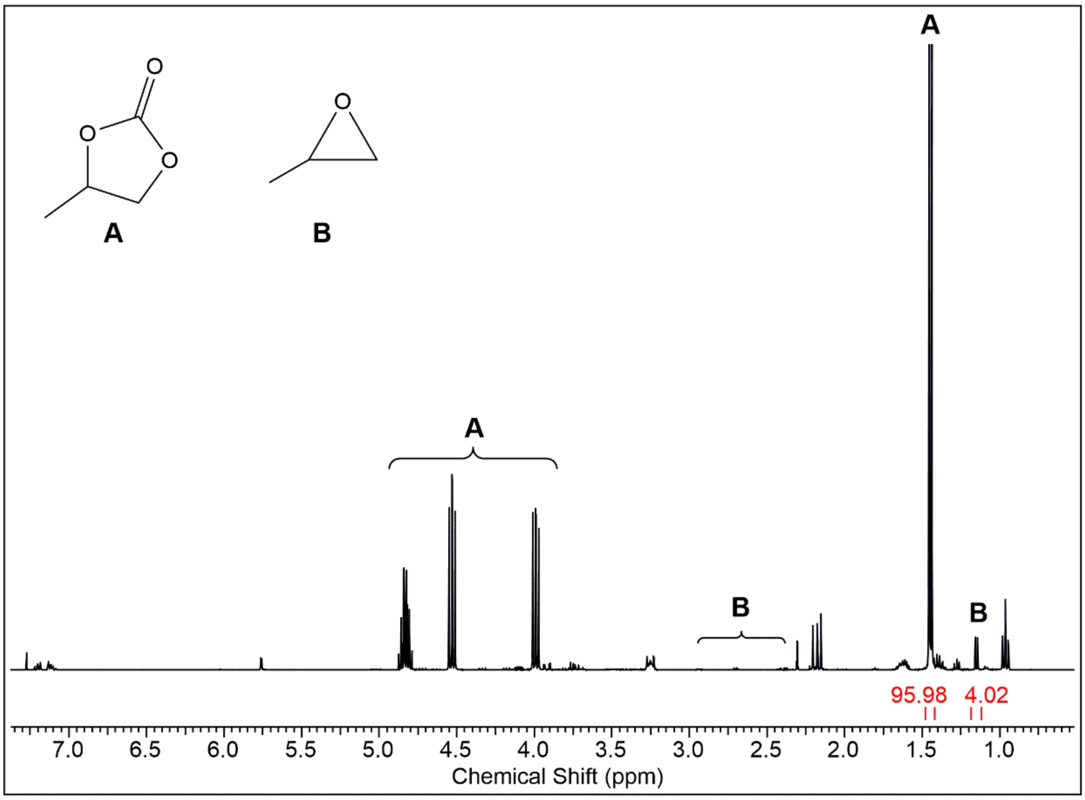

Figure S45. ^{1}H NMR (26 °C, 400.13 MHz, chloroform-*d*) of the product mixture of the catalytic formation of propylene carbonate using 0.25 mol% $[Ce_4(Me_2pz{\cdot}CO_2)_{12}]{\cdot}10$ toluene (**5·toluene**) as a catalyst. The conversion was determined by the integral ratio of the methyl protons in propylene oxide and propylene carbonate.

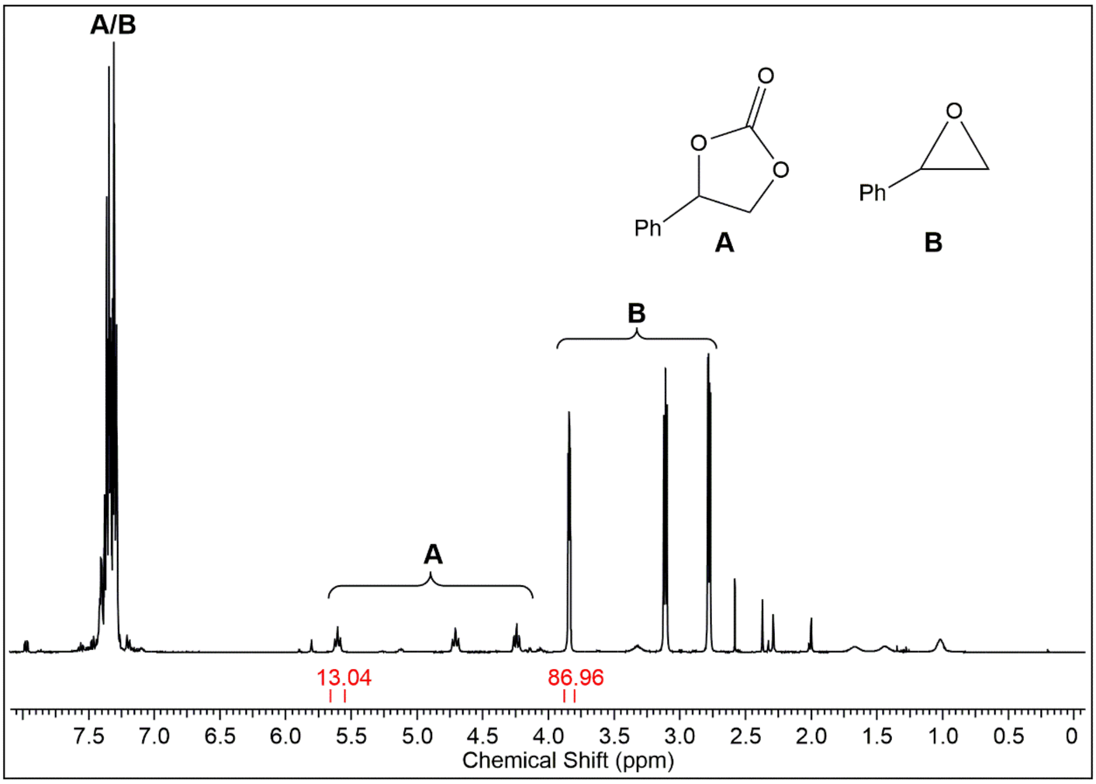

Figure S46. ^{1}H NMR (26 °C, 400.13 MHz, chloroform-*d*) of the product mixture of the catalytic formation of styrene carbonate using 0.25 mol% $[Ce_4(Me_2pz)_{12}]$ (**4**) as a catalyst. The conversion was determined by the integral ratio of the protons in α-position in styrene oxide and styrene carbonate.

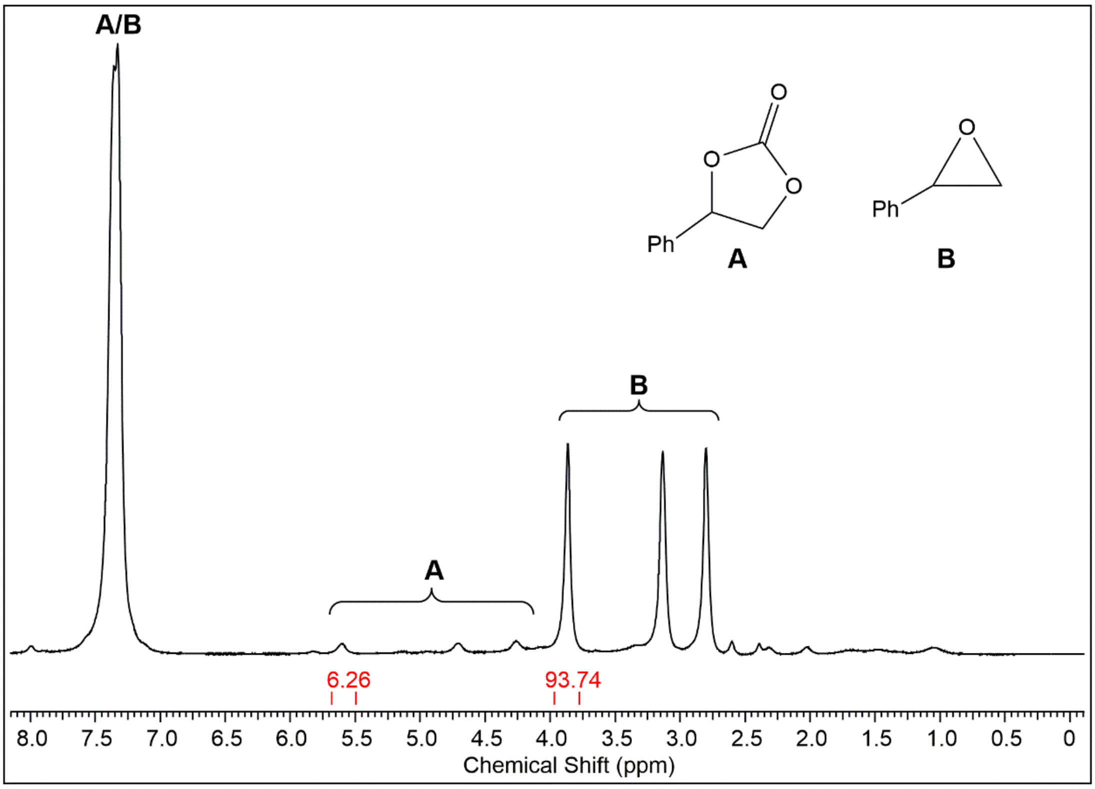

Figure S47. ^{1}H NMR (26 °C, 400.13 MHz, chloroform-*d*) of the product mixture of the catalytic formation of styrene carbonate using 0.125 mol% $[Ce_4(Me_2pz)_{12}]$ (**4**) as a catalyst. The conversion was determined by the integral ratio of the protons in α-position in styrene oxide and styrene carbonate.

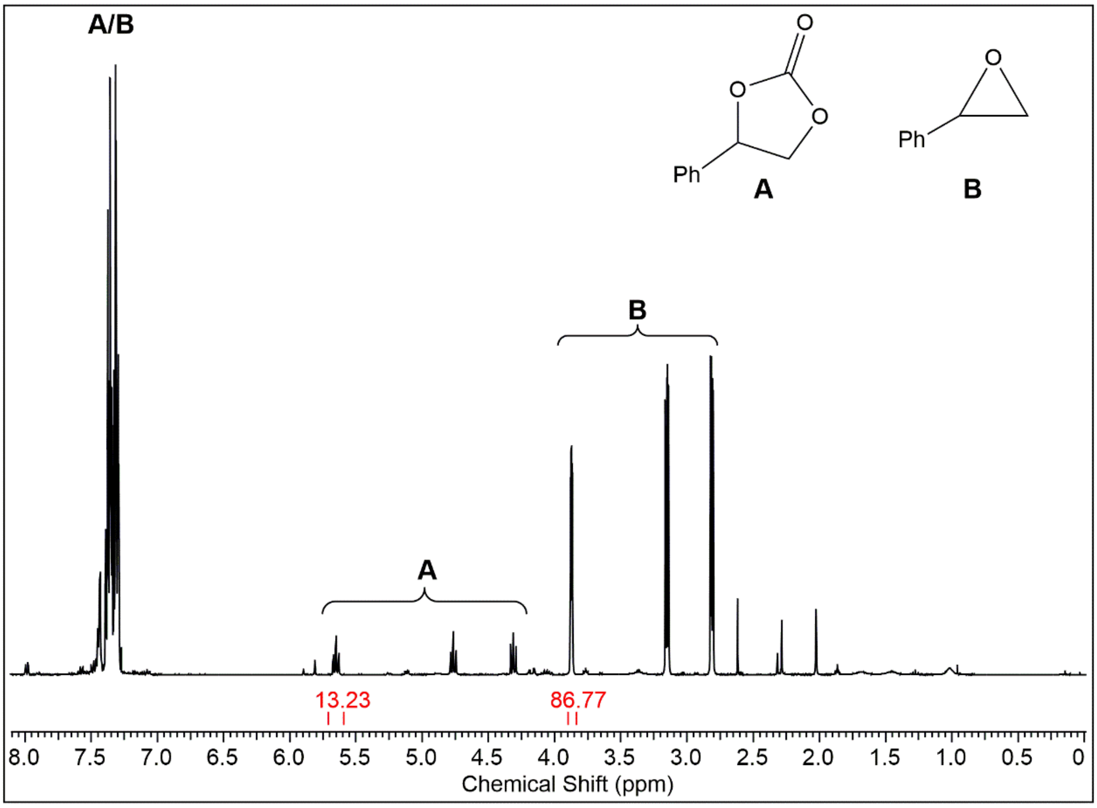

Figure S48. ^{1}H NMR (26 °C, 400.13 MHz, chloroform-*d*) of the product mixture of the catalytic formation of styrene carbonate using 0.5 mol% $[Ce(Me_2pz)_3(thf)]_2$ (**4·thf**) as a catalyst. The conversion was determined by the integral ratio of the protons in α-position in styrene oxide and styrene carbonate.

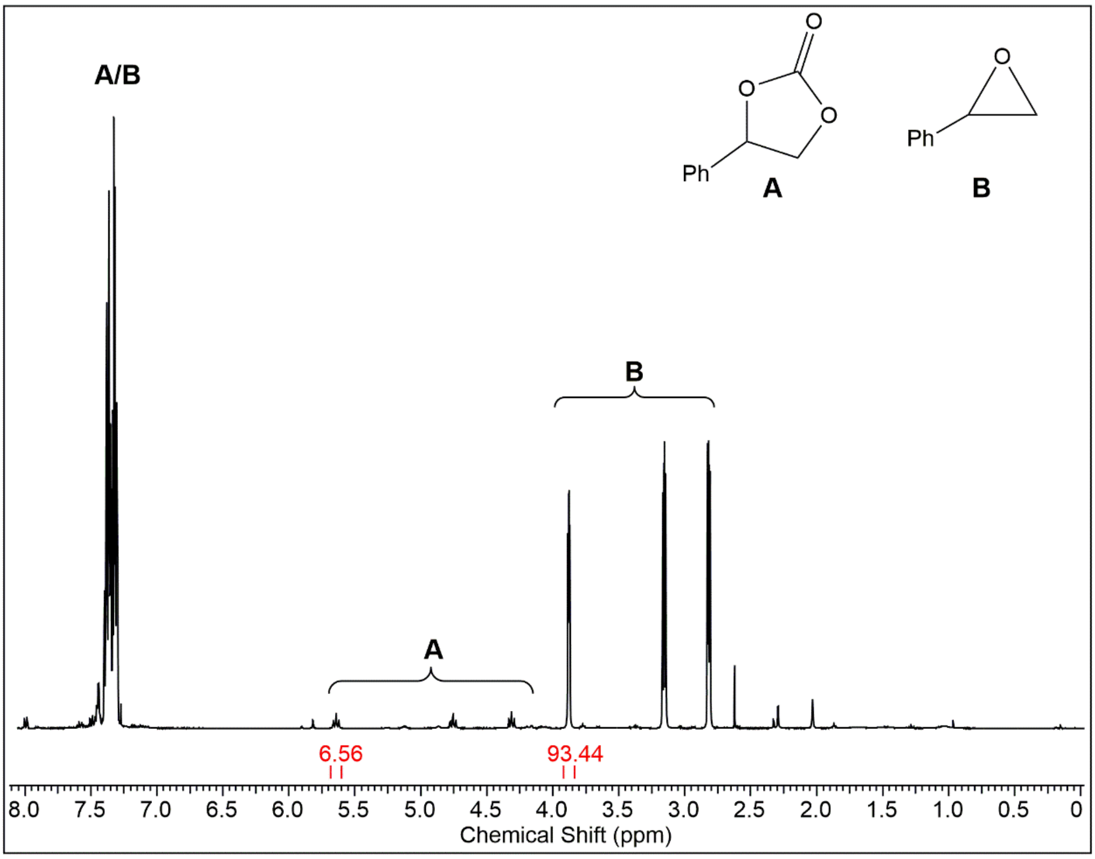

Figure S49. ^{1}H NMR (26 °C, 400.13 MHz, chloroform-*d*) of the product mixture of the catalytic formation of styrene carbonate using 0.25 mol% [Ce(Me$_2$pz)$_3$(thf)]$_2$ (**4·thf**) as a catalyst. The conversion was determined by the integral ratio of the protons in α-position in styrene oxide and styrene carbonate.

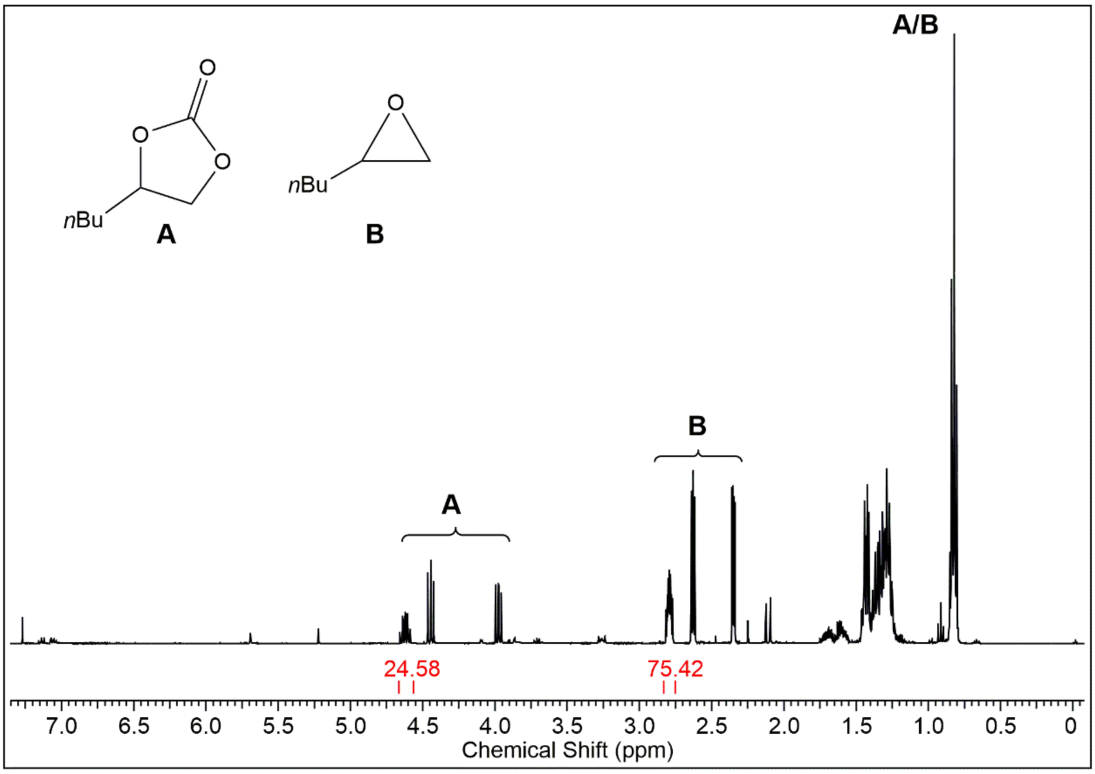

Figure S50. ^{1}H NMR (26 °C, 400.13 MHz, chloroform-*d*) of the product mixture of the catalytic formation of 1,2-*n*-hexylene carbonate using 0.25 mol% [Ce$_4$(Me$_2$pz)$_{12}$] (**4**) as a catalyst. The conversion was determined by the integral ratio of the protons in α-position in 1,2-*n*-hexylene oxide and 1,2-*n*-hexylene carbonate.

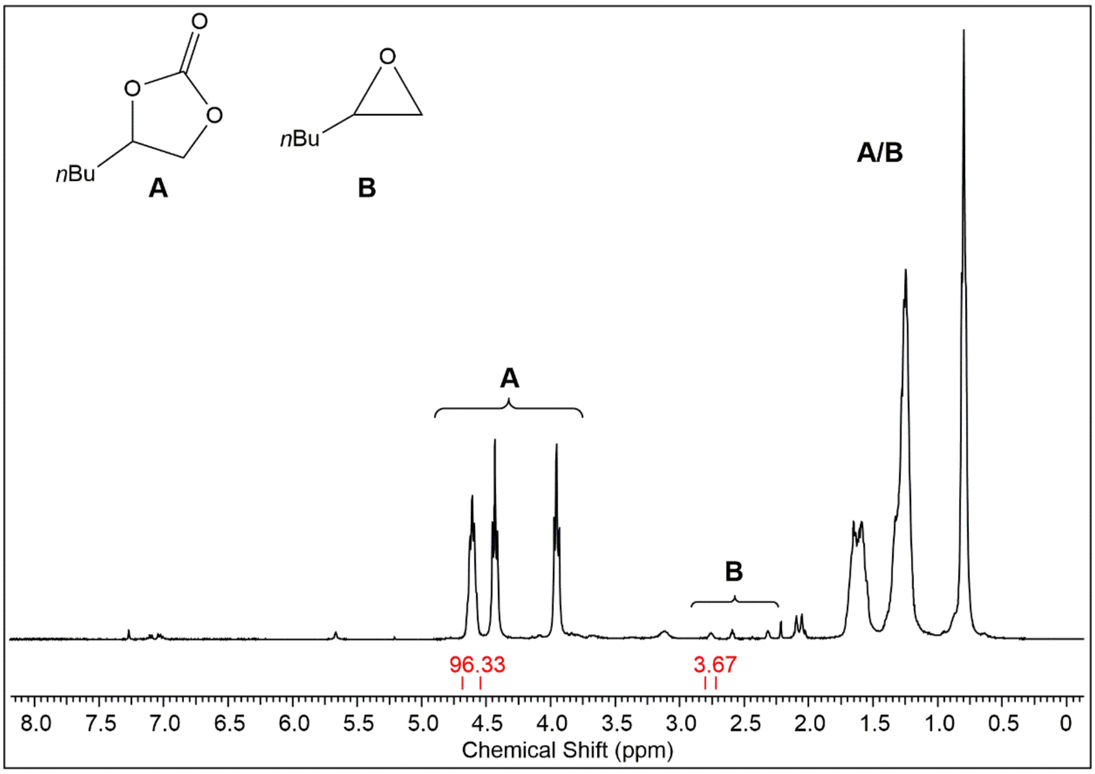

Figure S51. ^{1}H NMR (26 °C, 400.13 MHz, chloroform-*d*) of the product mixture of the catalytic formation of 1,2-*n*-hexylene carbonate using 0.25 mol% [$Ce_4(Me_2pz)_{12}$] (**4**) as a catalyst at 90 °C. The conversion was determined by the integral ratio of the protons in α-position in 1,2-*n*-hexylene oxide and 1,2-*n*-hexylene carbonate.

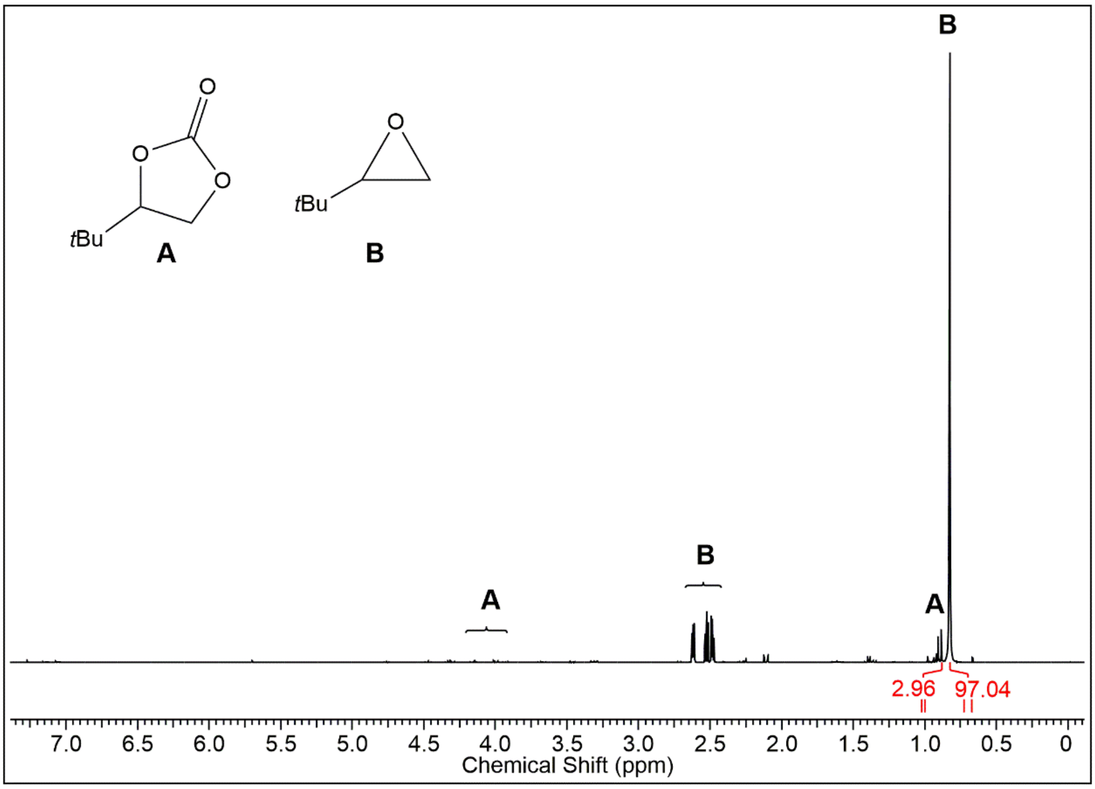

Figure S52. ^{1}H NMR (26 °C, 400.13 MHz, chloroform-*d)* of the product mixture of the catalytic formation of 3,3-dimethyl-1,2-butene carbonate using 0.25 mol% [$Ce_4(Me_2pz)_{12}$] (**4**) as a catalyst. The conversion was determined by the integral ratio of the *tert*-butyl protons in 3,3-dimethyl-1,2-butene oxide and 3,3-dimethyl-1,2-butylene carbonate.

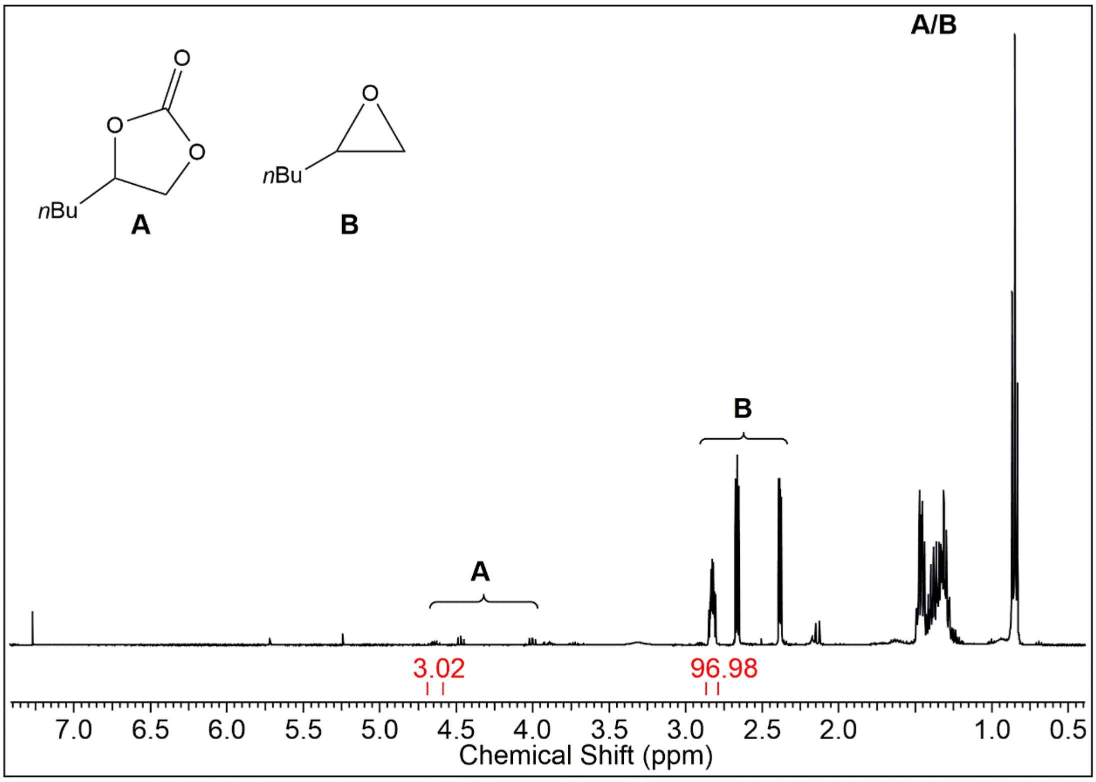

Figure S53. ^{1}H NMR (26 °C, 400.13 MHz, chloroform-*d*) of the product mixture of the catalytic formation of 1,2-*n*-hexylene carbonate using 1 mol% [Ce(Me$_2$pz)$_4$][NBu$_4$] (**6**) as a catalyst. The conversion was determined by the integral ratio of the protons in α-position in 1,2-*n*-hexylene oxide and 1,2-*n*-hexylene carbonate.

IR Spectra

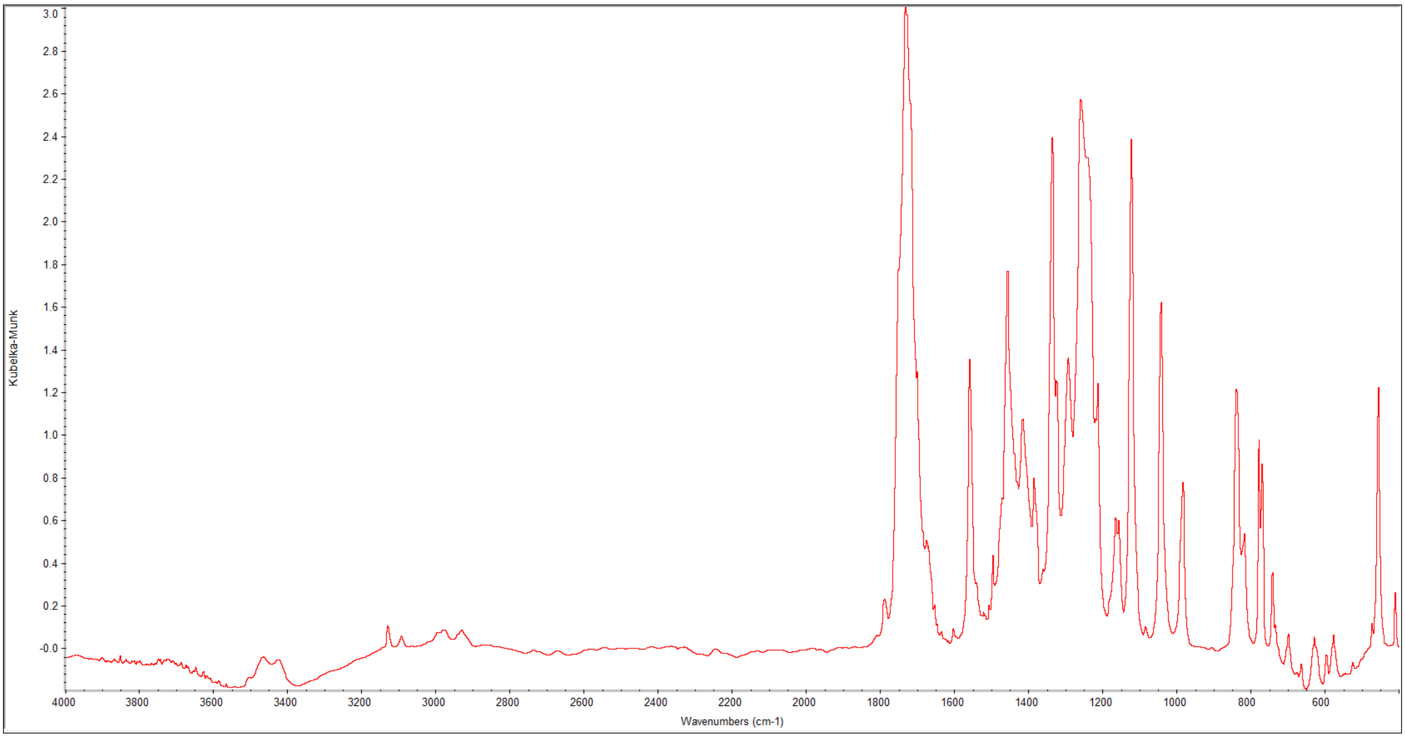

Figure S54. DRIFT spectrum of [Ce(Me_2pz·CO_2)$_4$]·2 tol (**2·toluene**) at 25 °C.

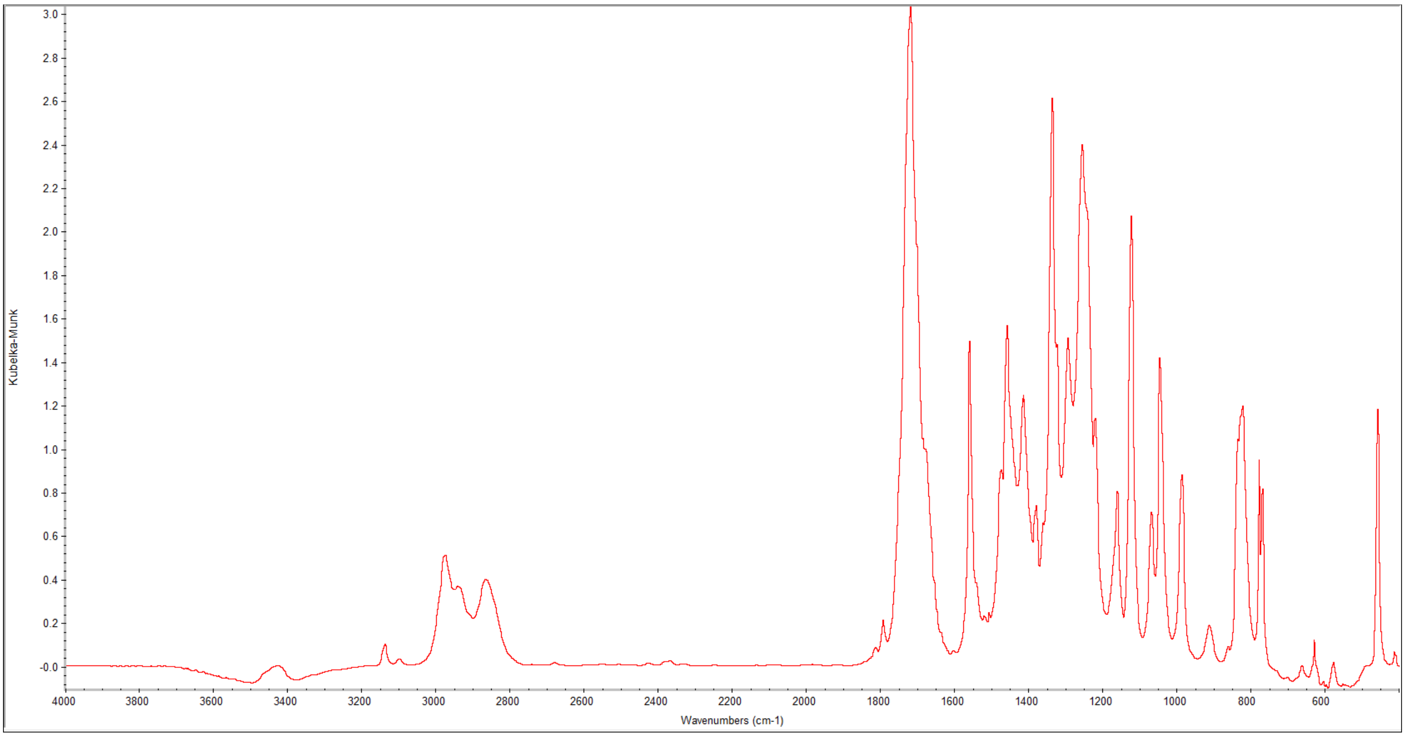

Figure S55. DRIFT spectrum of [Ce(Me_2pz·CO_2)$_4$]·2 thf (**2·thf**) at 25 °C.

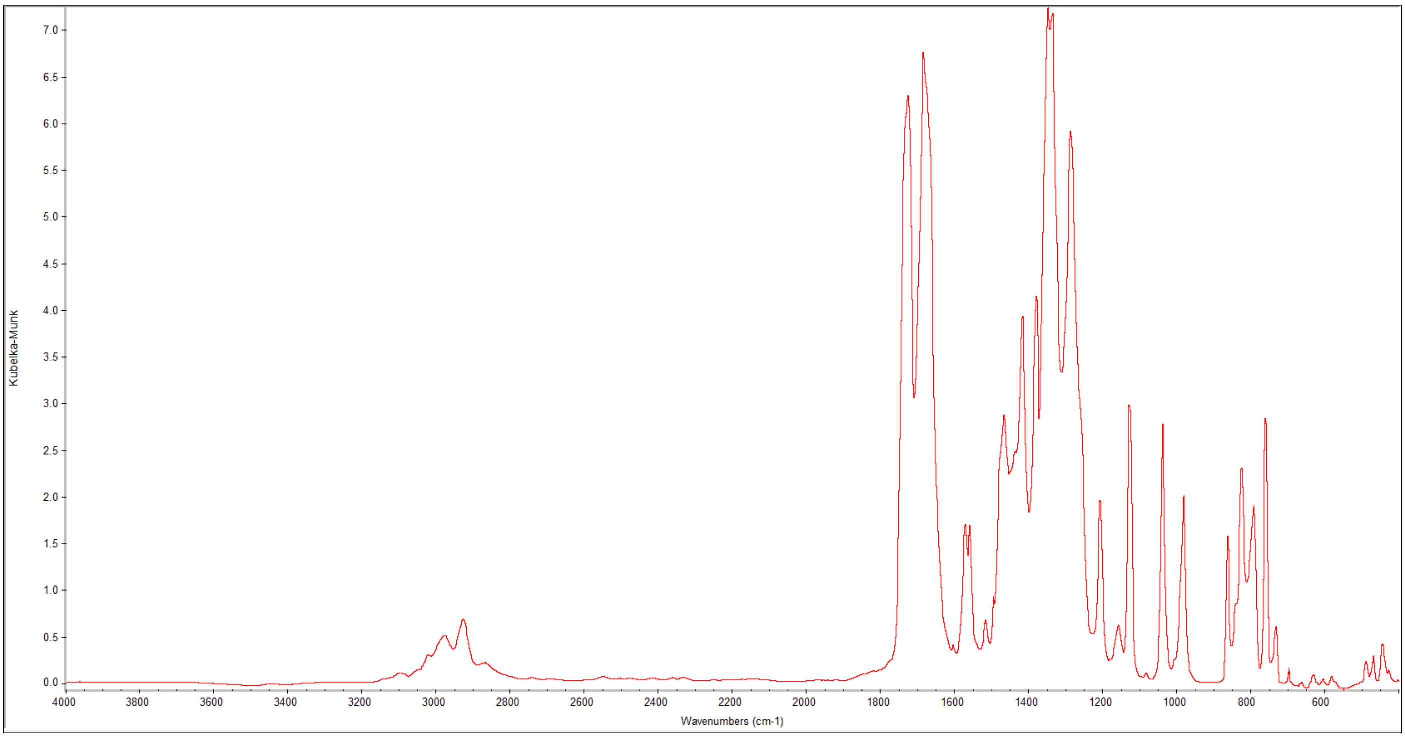

Figure S56. DRIFT spectrum of $[Ce_4(Me_2pz{\cdot}CO_2)_{12}]{\cdot}10$ toluene (**5·toluene**) at 25 °C.

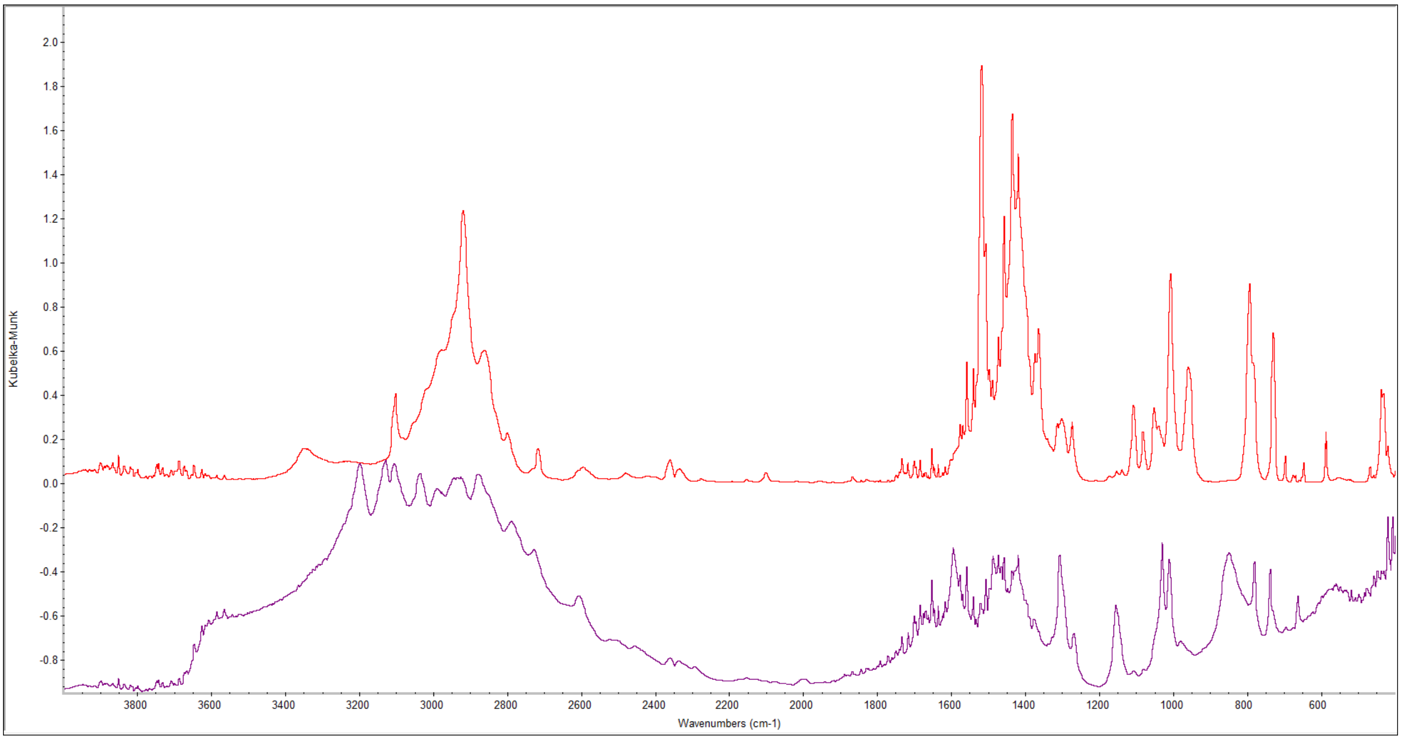

Figure S57. DRIFT spectra of $[Ce(Me_2pz)_4]_2$ stored for 3 days under 1 bar CO2 pressure (red trace) and $[Ce(Me_2pz)_4]_2$ after 30 min at ambient conditions (purple trace) at 25 °C.

In situ IR Measurements

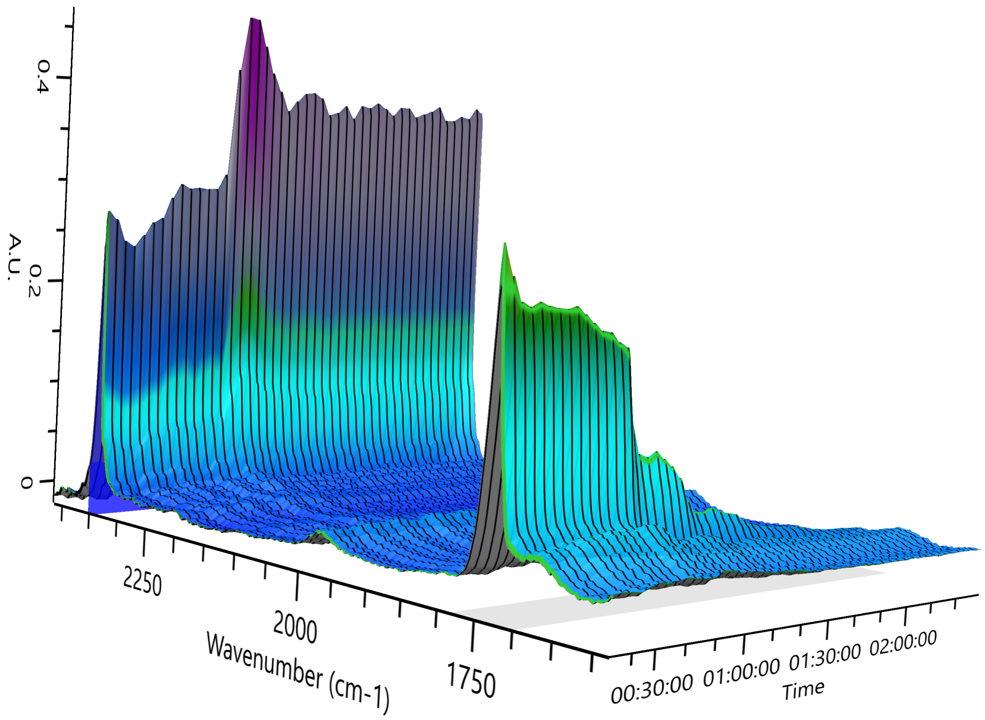

Figure S58. Stacked IR spectra of **2·thf** at 60 °C in the range of 1700 and 2350 cm^{-1}. Spectra were recorded every minute.

Thermogravimetric Analysis (TGA)

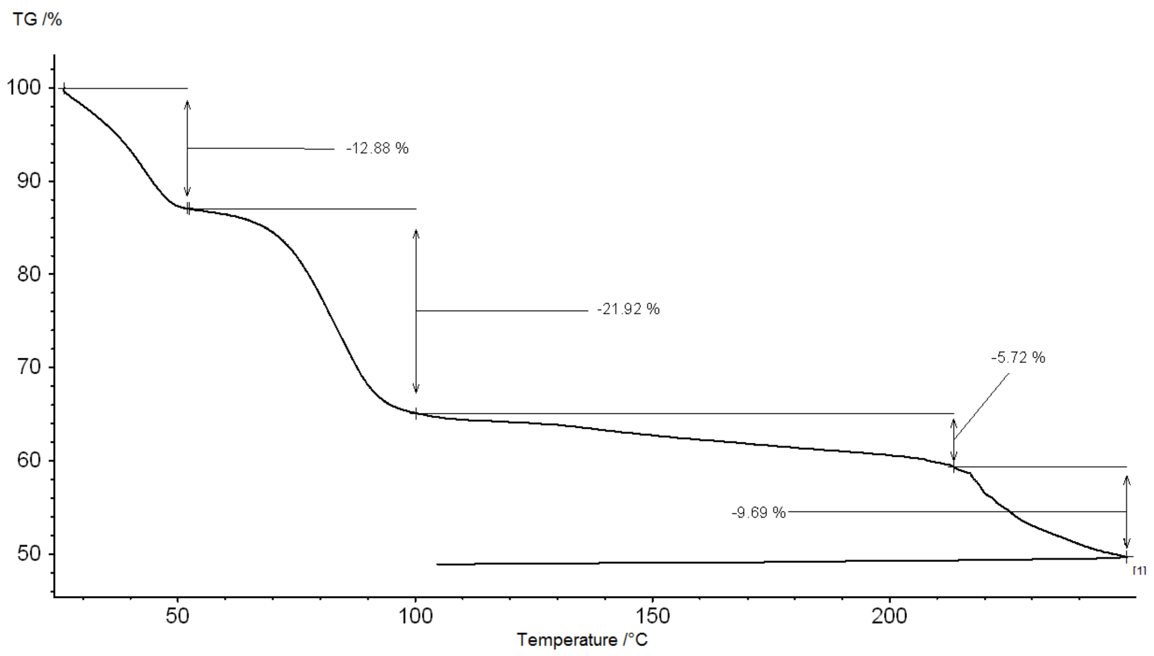

Figure S59. TGA of [Ce($Me_2pz·CO_2$)$_4$]·tol (**2·toluene**). Sample was heated from 28 °C to 250 °C with a heating rate of 0.5 K min^{-1} than cooled to ambient temperature with 10 K min^{-1}.

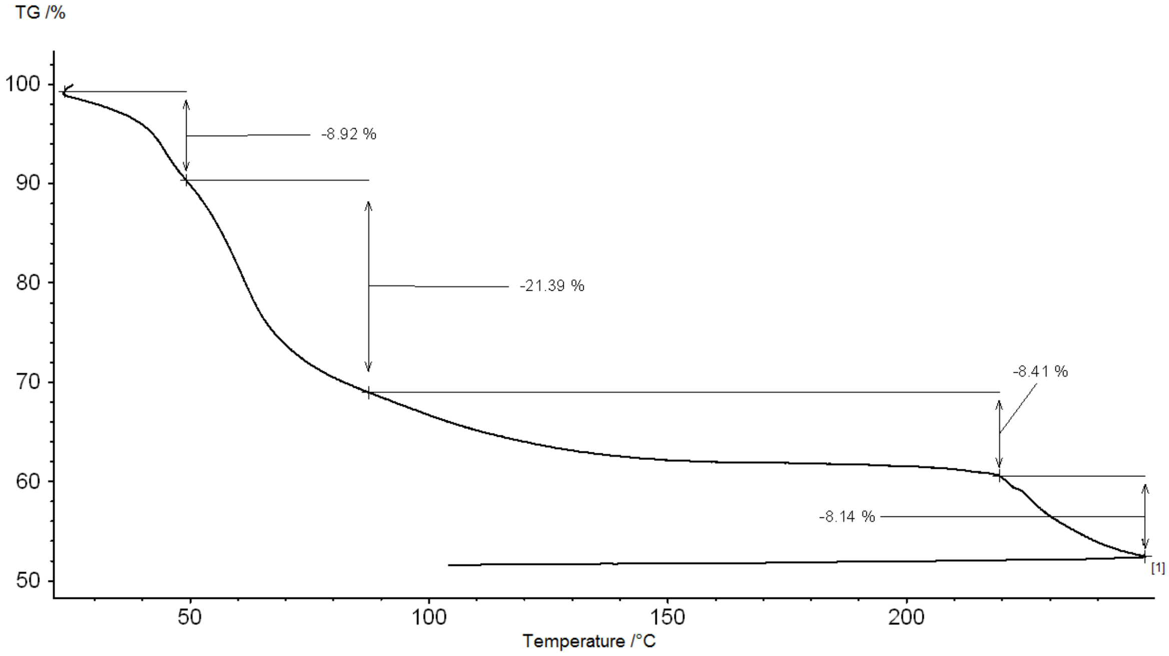

Figure S60. TGA of [Ce($Me_2pz·CO_2$)$_3$]$_4$·10 toluene (**5·toluene**). Sample was heated from 28 °C to 250 °C with a heating rate of 0.5 K min^{-1} than cooled to ambient temperature with 10 K min^{-1}.

Proposed Mechanisms of the Cycloaddition of CO_2 with Epoxides

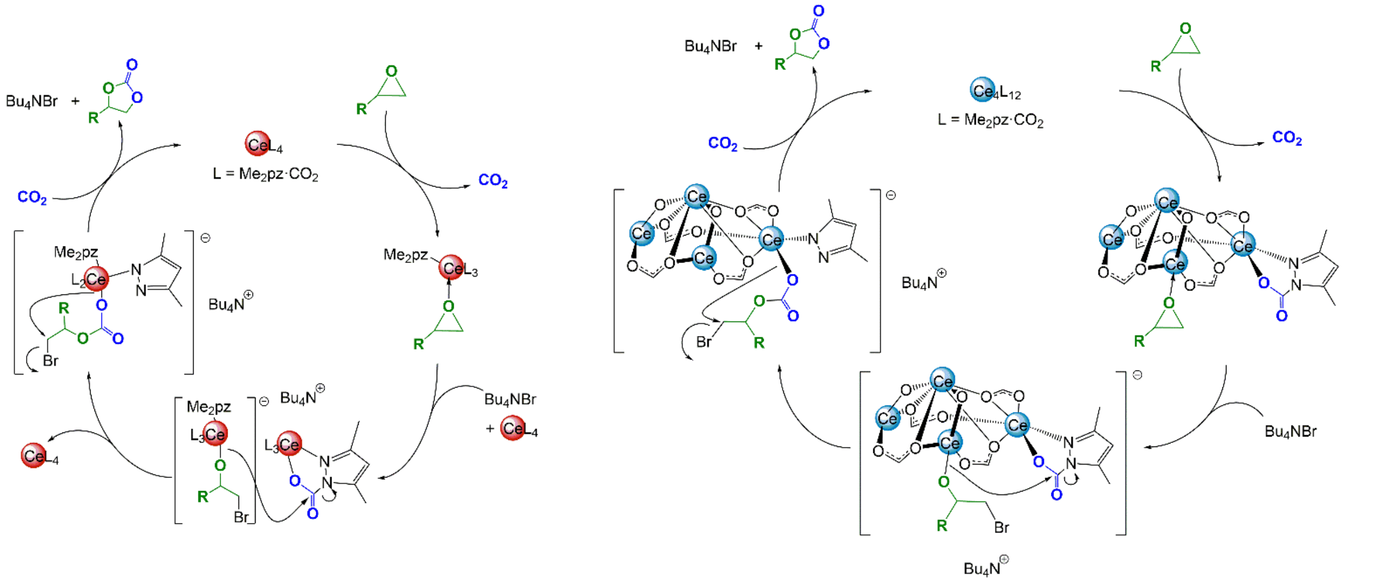

Scheme S1. Proposed mechanism for the cycloaddition of epoxides and carbon dioxide with tetravalent [Ce($Me_2pz{\cdot}CO_2$)$_4$] **2** (left) and trivalent [Ce_4($Me_2pz{\cdot}CO_2$)$_{12}$] **5** (right) as catalysts and TBAB as a cocatalyst.

Crystallography

Crystals for X-ray structure analysis were grown using saturated solutions of toluene (**2·toluene**, **3**), thf (**1-thf**, **2·thf**) or *n*-hexane (**5·toluene**) or a mixture of propylene oxide and propylene carbonate (**6**). Suitable crystals for were handpicked in a glovebox, coated with Parabar 10312 and stored on microscope slides and mounted rapidly outside the box onto a microloop Data collection were done on a *Bruker* APEX II Duo diffractometer by using QUAZAR optics and Mo K_α (λ = 0.71073 Å). The data collection strategy was determined using COSMO[5] employing ω scans. Raw data were processed by APEX 3[6] and SAINT,[7] corrections for absorption effects were applied using SADABS.[7] The structures were solved by direct methods and refined against all data by full-matrix least-squares methods on F^2 using SHELXTL[9] and SHELXLE or OLEX 2.[10,11] All atoms were refined anisotropically. The disorders of the solvent molecules were modelled using DSR,[12] a program to refinement of disordered structures with SHELXL. For compound **5** a global restrain (RIGU) was applied to all atoms to achieve a more chemically reasonable model. There are six disordered solvent toluenes in the asymmetric unit. Twinning was found for complex **6** and refinement in hklf 5 format was done. Plots were generated by using CCDC Mercury 3.19.1.[13] Further details regarding the refinement and crystallographic data are listed in Table S2 and in the CIF files.

Table S2. Crystallographic data for compounds **1·thf**, **2·toluene**, **2·thf**, **3**, **5·toluene**, and **6**

	**1-thf**	**2·toluene**	**2·toluene**	**2·thf**
**CCDC**	1540353	1959858	1959855	1959856
**formula**	$C_{24}H_{36}CeN_8O$	$C_{31}H_{36}CeN_8O_8$	$C_{24}H_{28}CeN_8O_8$	$C_{32}H_{44}CeN_8O_{10}$
**M [g·mol^{-1}]**	592.73	788.80	696.66	840.87
**λ [Å]**	0.71073	0.71073	0.71073	0.71073
**color**	red/ block	red/ block	yellow/ plate	yellow/ plate
**crystal dimensions [mm]**	0.3 × 0.3 × 0.3	0.199 × 0.174 × 0.143	0.218 × 0.146 × 0.089	0.206 × 0.204 × 0.113
**crystal system**	monoclinic	orthorhombic	orthorhombic	orthorhombic
**space group**	$P2_1/m$	$P2_12_12_1$	Aba2	Aba2
**a [Å]**	8.5694(16)	14.4389(3)	12.4084(14)	12.4385(8)
**b [Å]**	17.879(3)	14.8511(3)	12.5664(14)	12.9350(9)
**c [Å]**	8.7521(15)	15.5578(3)	25.508(3)	22.6128(15)
**β [°]**	91.013(3)			
**V [Å^3]**	1340.7(4)	3336.11(12)	3977.4(8)	3638.2(4)
**Z**	2	4	4	4
**F(000)**	604	1600	1400	1720
**T [K]**	100(2)	100(2)	180(2)	150(2)
**ρ_{calcd} [g·cm^{-3}]**	1.468	1.570	1.163	1.535
**μ[mm^{-1}]**	1.729	1.426	1.188	1.317
**Data / restraints / parameters**	2447 / 12 / 182	11095 / 0 / 442	4880 / 1 / 190	5067 / 214 / 282
**Goodness of fit**	1.055	1.042	0.988	1.034
**R_1 (I > 2σ (I))[a]**	0.0311	0.0186	0.0233	0.0273
**$_\omega R_2$ (all data)[b]**	0.0685	0.0439	0.0596	0.0789

[a] $R_1 = \Sigma(||F0|-|Fc||)/\Sigma|F0|, F0> 4s(F0)$. $_\omega R_2 = \{\Sigma[w(F02-Fc2)2/\Sigma[w(F02)^2]\}^{1/2}$

Table S2 continued:

	**3**	**5**	**6**
**CCDC**	1959857	1959860	1959859
**formula**	$C_{73}H_{106}Ce_3N_{24}O_7$	$C_{142}H_{164}Ce_4N_{24}O_{24}$	$C_{40}H_{64}CeN_9O_8$
**M [g·mol^{-1}]**	1852.18	3151.44	939.12
**λ [Å]**	0.71073	0.71073	0.71073
**color**	red/ block	colorless/ block	colorless/ block
**crystal dimensions [mm]**	0.21 × 0.19 × 0.18	0.311 × 0.211 × 0.078	0.063 × 0.047 × 0.038
**crystal system**	triclinic	monoclinic	triclinic
**space group**	$P\bar{1}$	$P2_1/n$	P1
**a [Å]**	14.463(2)	15.5082(12)	9.725(18)
**b [Å]**	14.757(2)	47.275(4)	9.843(18)
**c [Å]**	22.630(3)	19.4079(15)	13.02(2)
**α [°]**	106.365(2)	90	106.19(2)
**β [°]**	95.252(2)	94.6410(10)	108.52(2)
**γ [°]**	110.714(2)	90	90.58(2)
**V [Å^3]**	4235.2(11)	14182.3(19)	1128(4)
**Z**	2	4	1
**F(000)**	1884	6432	489
**T [K]**	100(2)	100(2)	100(2)
**ρ_{calcd} [g·cm^{-3}]**	1.452	1.476	1.383
**μ[mm^{-1}]**	1.648	1.336	1.067
**Data / restraints / parameters**	21905 / 6 / 990	29142 / 9288 / 2151	6581 / 537 / 536
**Goodness of fit**	1.019	1.293	1.034
**R_1 (I > 2σ (I))[a]**	0.0305	0.0852	0.0683
**$_{\omega}R_2$ (all data)[b]**	0.0698	0.1636	0.1792

[a] $R_1 = \Sigma(||F0|-|Fc||)/\Sigma|F0|, F0> 4s(F0)$. $_{\omega}R_2 = \{\Sigma[w(F02-Fc2)2/\Sigma[w(F02)^2]\}^{1/2}$

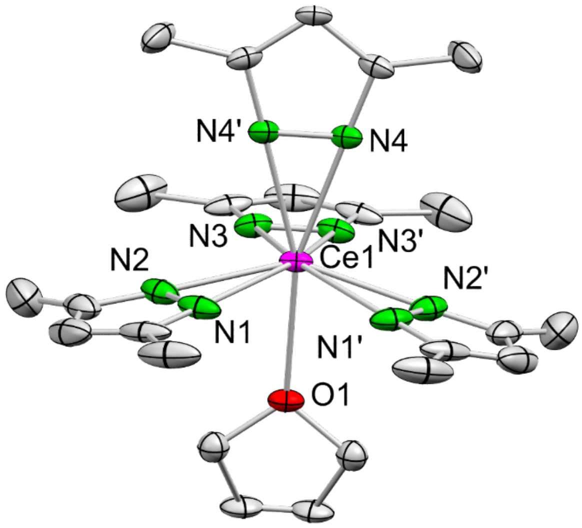

Figure S61. Crystal structure of [Ce(Me_2pz)$_4$(thf)] (**1-thf**). Ellipsoids are shown at the 50 % probability level. Hydrogen atoms and disordered thf are omitted for clarity. Selected bond lengths [Å]: Ce1–N1 2.384(3), Ce1–N2 2.367(3), Ce1–N3 2.364(3), Ce1–N4 2.356(3), Ce1–O1 2.494(4).

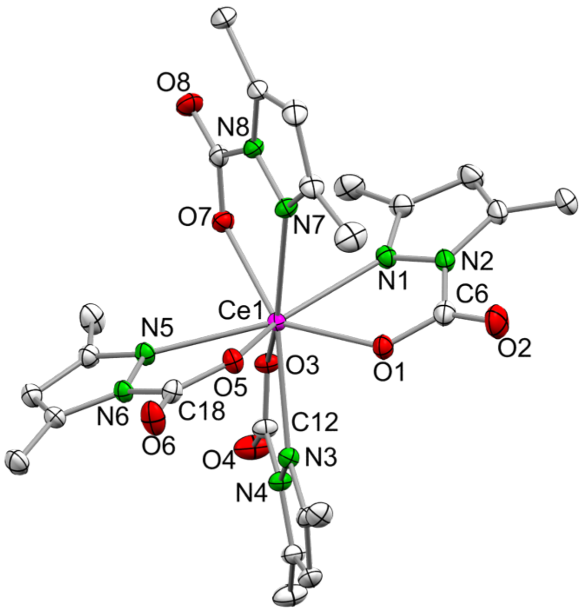

Figure S62. Crystal structure of [Ce(Me_2pz·CO_2)$_4$] (**2·toluene**). Ellipsoids are shown at the 50 % probability level. Hydrogen atoms and toluene are omitted for clarity. Selected bond lengths [Å]: Ce1–N1 2.530(2), Ce1–N3 2.539(2), Ce1–N5 2.504(2), Ce1–N7 2.538(2), Ce1–O1 2.247(1), Ce1–O3 2.263(1), Ce1–O5 2.244(2), Ce1–O7 2.265(1), C6–O1 1.293(2), C6–O2 1.201(3), C12–O3 1.293(2), C12–O4 1.209(3), C18–O5 1.289(2), C18–O6 1.204(3), C24–O7 1.289(3), C24–O8 1.212(3). Selected angles [°]: O-Ce-N (bite) 64.06(5)-64.68(5).

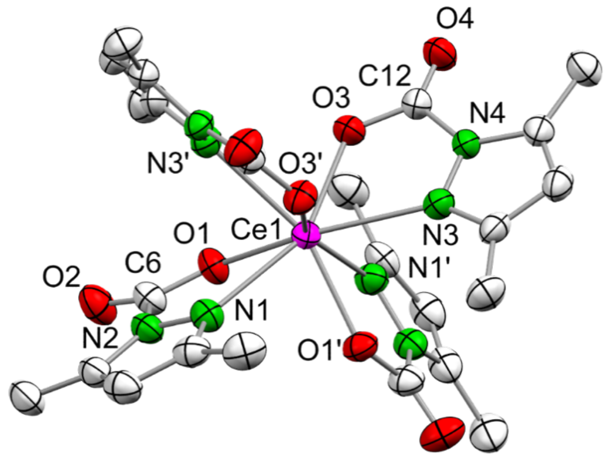

Figure S63. Crystal structure of [Ce(Me_2pz·CO_2)$_4$]·2 thf (**2·thf**). Ellipsoids are shown at the 50 % probability level. Hydrogen atoms and toluene are removed for clarity. Selected bond lengths [Å]: Ce1–N1 2.516(4), Ce1–N3 2.521(4), Ce1–O1 2.256(4), Ce1–O3 2.246(4), C6−O1 1.312(6), C6–O2 1.188(6), C12–O3 1.308(6), C12–O4 1.190(6). Selected angles [°]: O-Ce-N (bite) 64.95(13)-64.98(13).

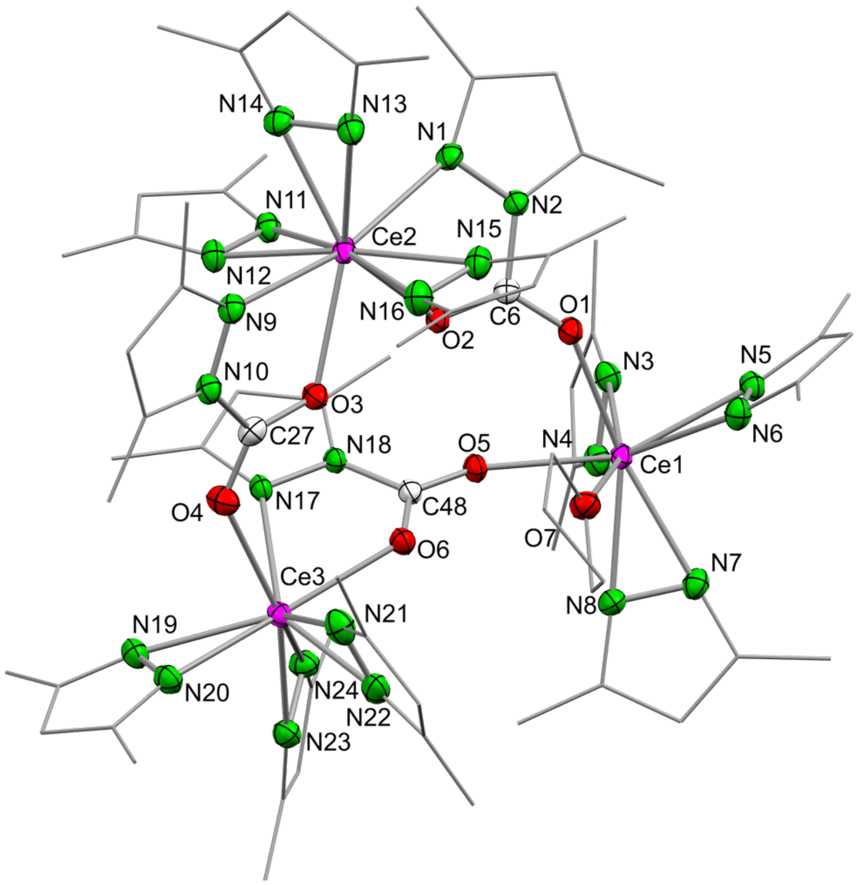

Figure S64. Crystal structure of [Ce_3(Me_2pz)$_9$(Me_2pz·CO_2)$_3$(thf)] (**3**). Ellipsoids are shown at the 50 % probability level. Hydrogen atoms and *n*-hexane are omitted for clarity. Selected bond lengths [Å]: Ce1–O1 2.374(2), Ce1–O5 2.360(2), Ce1–N3-8 2.356(2)–2.399(2), Ce2–O2 2.436(2), Ce2–O3 2.394(2), Ce2–N1 2.659(2), Ce2–N9 2.645(2), Ce2–N11-16 2.355(2)–2.442(2), Ce3–O4 2.381(2), Ce3–O6 2.372(2), Ce3–N17 2.588(2), Ce3–N19-24 2.327(2)–2.411(2), C6–O1 1.252(3), C6–O2 1.244(3), C27–O3 1.243(3), C27–O4 1.247(3), C48–O5 1.244(3), C48–O6 1.252(3). Selected angles [°]: O-Ce-N bite 61.72(6)-63.69(6).

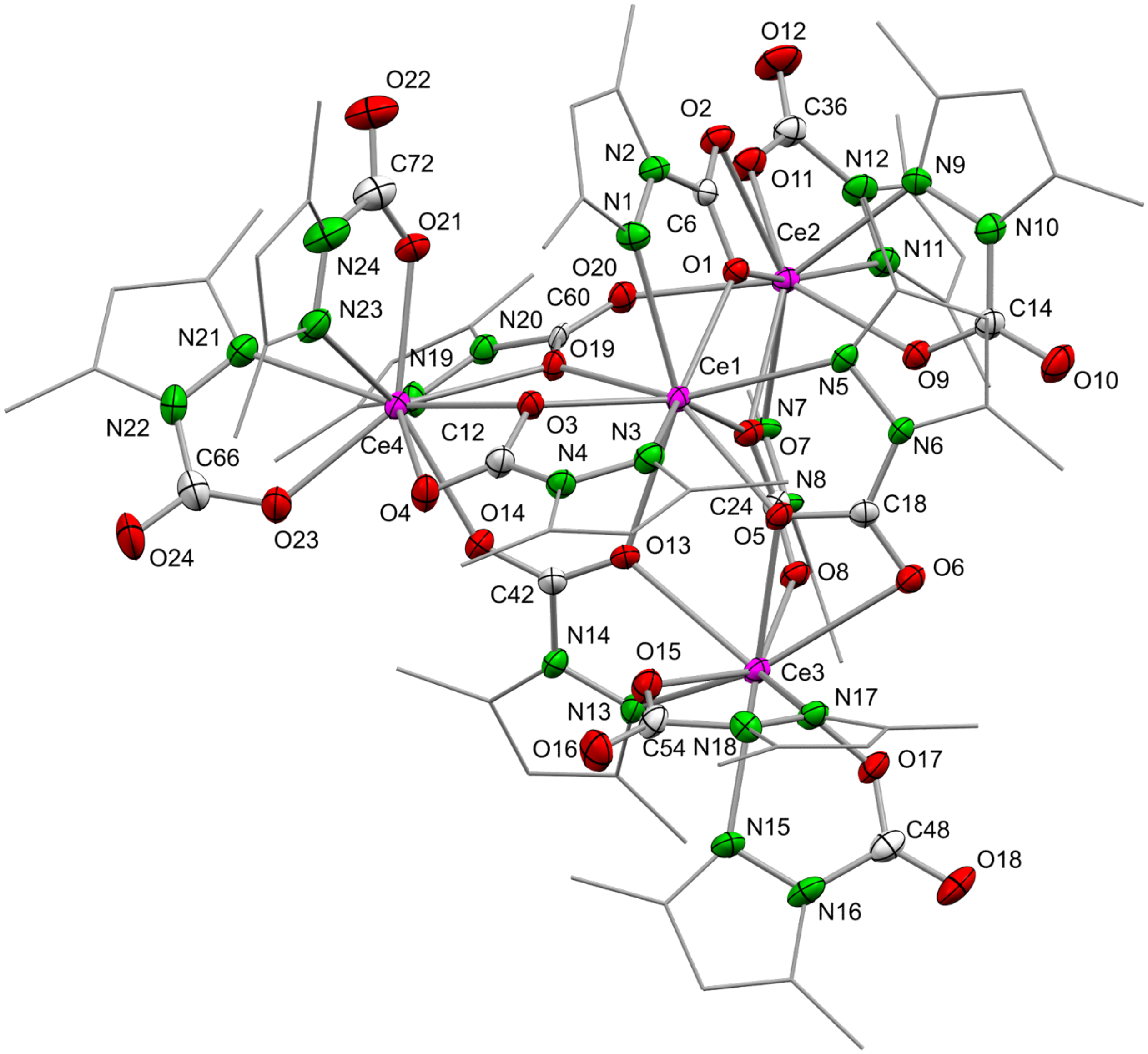

Figure S65. Crystal structure of [$Ce_4(Me_2pz\cdot CO_2)_{12}$] (**5**). Ellipsoids are shown at the 50 % probability level. Hydrogen atoms and toluene are omitted for clarity. Selected bond lengths [Å]: Ce1–O1/O3/O5 2.386(5)–2.399(5), Ce1–O7/O13/O19 2.579(6)–2.625(5), Ce1–N1/N3/N5 2.688(7)–2.724(7), Ce2–O1/O20 2.473(6)–2.515(5), Ce2–O2/O7 2.675(5) - 2.771(6), Ce2–O9/O11 2.373(5)–2.387(5), Ce2–N7/N9/N11 2.617(7)–2.673(7), Ce3–O5/O8 2.495(6)–2.503(5), Ce3–O6/O13 2.661(5)–2.750(6), Ce3–O15/O17 2.366(5)–2.386(6), Ce3–N13/N15/N17 2.636(7)–2.671(7), Ce4–O3/O14 2.474(6)–2.524(5), Ce4–O4/O19 2.665(5)–2.743(6), Ce4–O21/O23 2.364(6)–2.389(6), Ce4–N19/N21/N23 2.614(8)–2.658(7). Selected angles [°]: O-Ce-N bite 59.43(19)-63.1(2).

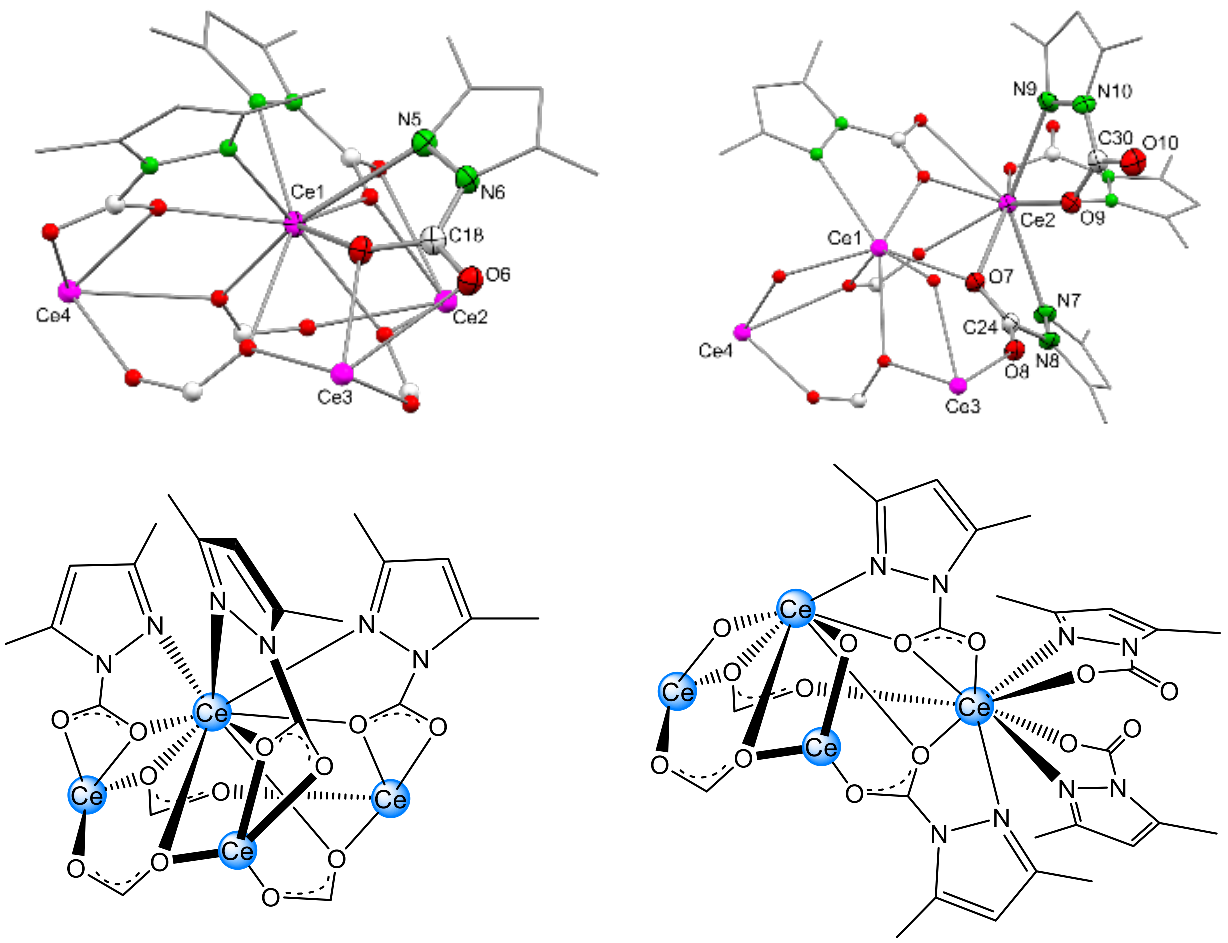

Figure S66. Top: Cutouts of the crystal structure of $[Ce_4(Me_2pz{\cdot}CO_2)_{12}]_4$ (**5**), showing the surrounding of Ce1 and Ce2. Bottom: Schematic view of different $Me_2Pz{\cdot}CO_2$ binding modes in complex **5**.

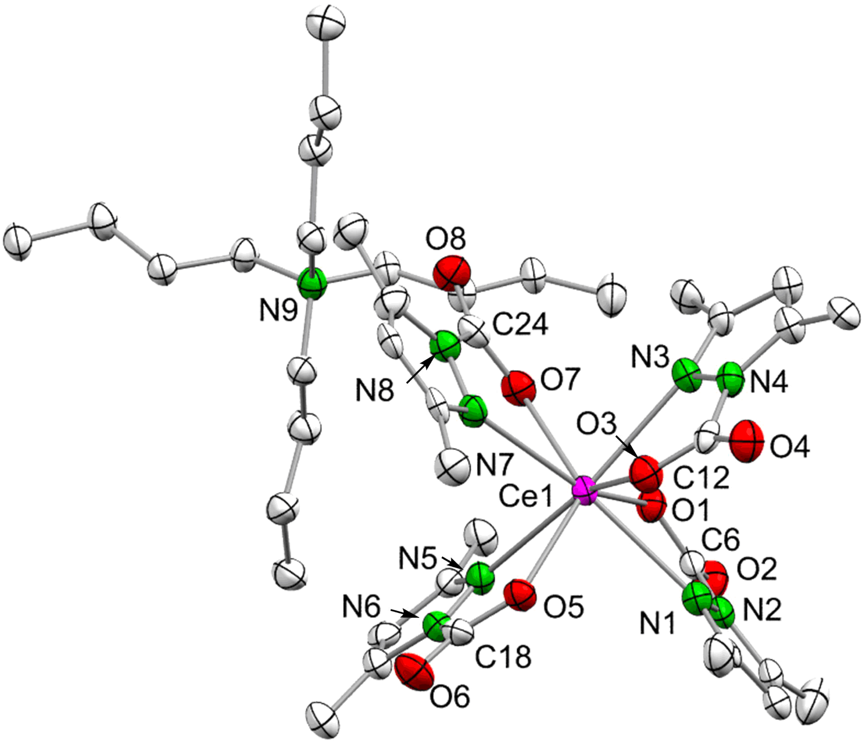

Figure S67. Crystal structure of [Ce(Me_2pz·CO_2)$_4$][NBu_4] (**6**). Ellipsoids are shown at the 50 % probability level. Hydrogen atoms and toluene are omitted for clarity. Selected bond lengths [Å]: Ce1–N1 2.64(2), Ce1–N3 2.62(2), Ce1–N5 2.65(2), Ce1–N7 2.61(2), Ce1–O1 2.40(1), Ce1–O3 2.41(2), Ce1–O5 2.40(1), Ce1–O7 2.40(1), C6–O1 1.27(2), C6–O2 1.21(2), C12–O3 1.26(2), C12–O4 1.20(2), C18–O5 1.26(2), C18–O6 1.22(2), C24–O7 1.29(3), C24–O8 1.20(2). Selected angles [°]: O-Ce-N bite 61.6(5)-62.3(5).

References

[1] D. Werner, G. B. Deacon, P. C. Junk, R. Anwander, *Dalton Trans.* **2017**, *46*, 6265–6277.

[2] G.B. Deacon, R. Harika, P.C. Junk, B.W. Skelton, D. Werner, A.H. White, *Eur. J. Inorg. Chem.* **2014**, 2412-2419.

[3] D. Werner, U. Bayer, N. E. Rad, P. C. Junk, G. B. Deacon, R. Anwander, *Dalton Trans.* **2018**, *47*, 5952–5955.

[4] G. R. Fulmer, A. J. M. Miller, N. H. Sherden, H. E. Gottlieb, A. Nudelman, B. M. Stoltz, J. E. Bercaw, K. I. Goldberg, *Organometallics* **2010**, *29*, 2176–2179.

[5] COSMO, v. 1.61; Bruker AXS Inc., Madison, WI, 2012.

[6] APEX 3, v. 2016.5-0; Bruker AXS Inc., Madison, WI, 2012.

[7] SAINT, v. 8.34A; Bruker AXS Inc., Madison, WI, 2010.

[8] L. Krause, R. Herbst-Irmer, G. M. Sheldrick, D. Stalke, *J. Appl. Cryst.* **2015**, *48*, 3-10.

[9] a) G. M. Sheldrick, *SHELXS: Acta Cryst.* **2008**, *A64*, 112-122; b) G. M. Sheldrick, *Acta Crystallogr.*, Sect. A **2015**, *71*, 3-8.

[10] C. B. Hübschle, G. M. Sheldrick, B. J. Dittrich, *J. Appl. Cryst.* **2011**, *44*, 1281-1284.

[11] O. V. Dolomanov, L. J. Bourhis, R. J. Gildea, J. A. K. Howard, H. Puschmann, *J. Appl. Cryst.* **2009**, *42*, 339-341.

[12] D. Kratzert, J. J. Holstein, I. Krossing, DSR: enhanced modelling and refinement of disordered structures with SHELXL. *J. Appl. Cryst.* **2015,** *48*, 933-938.

[13] C. F. Macrae, I. J. Bruno, J. A. Chisholm, P. R. Edgington, P. McCabe, E. Pidcock, L. Rodriguez-Monge, R. Taylor, J. van de Streek, P. A. Wood, *J. Appl. Cryst.* **2008**, *41*, 466-470.
